# Transcriptome Profiling of the Liver in Nellore Cattle Phenotypically Divergent for RFI in Two Genetic Groups

**DOI:** 10.3390/ani13030359

**Published:** 2023-01-20

**Authors:** Marta Serna-García, Larissa Fernanda Simielli Fonseca, Joaquin Javier Panadero Romero, Julian Carretero Asuncion, Danielly Beraldo dos Santos Silva, Bruna Maria Salatta, Gabriela Bonfá Frezarim, Maria Eugênia Zerlotti Mercadante, Sarah Figueiredo Martins Bonilha, Jesus Aparecido Ferro, Lucia Galvão De Albuquerque

**Affiliations:** 1 School of Agricultural and Veterinary Sciences, Sao Paulo State University (UNESP), Jaboticabal 14884-900, São Paulo, Brazil; 2 Igenomix S.L., 46021 Valencia, Spain; 3 Department of Physiology, Faculty of Medicine and Odontology, University of Valencia, 46100 Valencia, Spain; 4 National Council for Scientific and Technological Development (CNPq), Brasília 71605-170, Distrito Federal, Brazil; 5 Institute of Animal Science, Sertãozinho 14160-970, Sao Paulo, Brazil

**Keywords:** bovine, biomarkers, feed efficiency, liver tissue, non-coding RNAs, RNA-Seq

## Abstract

**Simple Summary:**

The selection of genetically superior animals for residual feed intake (RFI) does not affect animal growth, reproduction or the meat quality but it does lead to animals with lower consumption and lower maintenance requirements. This trait is difficult and costly to measure and is influenced by different biological processes and multiple genes. We used the RNA seq methodology of the liver in Nellore cattle with divergent RFI in two genetic groups. Eighty-eight common, differentially-expressed genes were identified in the two genetic groups. We highlight the B2M gene participating in most cellular processes that differentiate groups with a greater or lesser food efficiency. The biological pathways associated with RFI in the two genetic groups were for energy metabolism, protein turnover, redox homeostasis and the immune response. Additionally, we found non-coding RNAs, highlighting microRNA 25, which can act by blocking cytotoxicity and oxidative stress, and RNase_MRP, as a blocker of mitochondrial damage. This work could help future approaches in quantitative methods of animal husbandry and management and allow the use of possible markers for selecting more efficient animals, thus helping meat production costs and environmental impacts.

**Abstract:**

The identification and selection of genetically superior animals for residual feed intake (RFI) could enhance productivity and minimize environmental impacts. The aim of this study was to use RNA-seq data to identify the differentially expressed genes (DEGs), known non-coding RNAs (ncRNAs), specific biomarkers and enriched biological processes associated with RFI of the liver in Nellore cattle in two genetic groups. In genetic group 1 (G1), 24 extreme RFI animals (12 low RFI (LRFI) versus 12 high RFI (HRFI)) were selected from a population of 60 Nellore bulls. The RNA-seq of the samples from their liver tissues was performed using an Illumina HiSeq 2000. In genetic group 2 (G2), 20 samples of liver tissue of Nellore bulls divergent for RFI (LRFI, *n* = 10 versus HRFI, *n* = 10) were selected from 83 animals. The raw data of the G2 were chosen from the ENA repository. A total of 1811 DEGs were found for the G1 and 2054 for the G2 (*p*-value ≤ 0.05). We detected 88 common genes in both genetic groups, of which 33 were involved in the immune response and in blocking oxidative stress. In addition, seven (*B2M*, *ADSS*, *SNX2*, *TUBA4A*, *ARHGAP18*, *MECR*, and *ABCF3*) possible gene biomarkers were identified through a receiver operating characteristic analysis (ROC) considering an AUC > 0.70. The *B2M* gene was overexpressed in the LRFI group. This gene regulates the lipid metabolism protein turnover and inhibits cell death. We also found non-coding RNAs in both groups. MIR25 was up-regulated and SNORD16 was down-regulated in the LRFI for G1. For G2, up-regulated RNase_MRP and SCARNA10 were found. We highlight MIR25 as being able to act by blocking cytotoxicity and oxidative stress and RMRP as a blocker of mitochondrial damage. The biological pathways associated with RFI of the liver in Nellore cattle in the two genetic groups were for energy metabolism, protein turnover, redox homeostasis and the immune response. The common transcripts, biomarkers and metabolic pathways found in the two genetic groups make this unprecedented work even more relevant, since the results are valid for different herds raised in different ways. The results reinforce the biological importance of these known processes but also reveal new insights into the complexity of the liver tissue transcriptome of Nellore cattle.

## 1. Introduction

The world population is concerned about the environment and fulfilling the Sustainable Development Goals (SDGs), such as SDG 13 (Climate Action); moreover, growing pressure from the media is putting the focus, in recent times, on livestock. Thirty-seven percent of greenhouse gas (GHG) emissions are partly brought about by the global food supply even though the world population, particularly the high-income countries, has been recommended to reduce the global meat consumption [1,2], despite the recommendation of the World Health Organization (WHO) to consume 500 g per week [3]. Brazil has the advantage of mostly having an extensive production system, which is more sustainable. It produces and exports most of the meat consumed by European countries, but it could benefit from increasing its market, as long as it seeks sustainable production [4]. Furthermore, Brazil is the second highest meat consumer per capita (42.12 kg/year) [5], 80% of which is beef consumption. In the context of climate change and more restrictive environmental legislation, bovine meat production is under close observation. The European market is particularly demanding with regard to increasing sustainability.

Feed intake on farms is approximately 60% of the total cost of a livestock farm, therefore, a selection of more efficient animals would reduce the production costs and increase the profitability of the agricultural system [6,7]. Residual feed intake (RFI) has been used to estimate the feed efficiency (FE) in beef cattle [8]. The RFI is defined as the difference between the observed and predicted dry matter intake with an average daily gain and metabolic body weight of a given feeding period [9]. A low or negative RFI value stands for a high feed efficiency, while a high or positive RFI value indicates a low efficiency. As a linear trait, RFI has been reported to be independent of growth, both phenotypically and genetically [10,11,12]. The more efficient animals ingest less feed than is estimated for the same weight gain compared with less efficient animals. Heritability estimates for feed efficiency-related traits are moderate in the Nellore breed [13]; however, it has a high cost and technical difficulty in measuring the trait [14].

The identification and selection of genetically superior animals for their RFI would reduce feed costs, thereby increasing profits and minimizing the environmental impact [15,16,17]. There are various studies on the RFI trait in bovines from different breeds, sexes, and management systems [18,19,20,21,22,23], but little experimental information has been published that is thorough enough to unravel the biological regulation of the trait. The expression of the potential for feed efficiency is complex and depends on the interaction of numerous biochemical pathways through a multitude of tissues [24]. The liver constitutes the main location of energy and substance metabolism [15]. It can maintain glucose homeostasis in the blood and participates in protein biosynthesis as well as immune and detoxification functions [25]. According to Ferrell and Jenkins [26], in beef cattle, 70% of the total energy is used for maintenance, which illustrates the need for understanding the biological mechanisms to optimize the regulation of maintenance. The non-coding RNAs have been shown to regulate gene expression [27]. Non-coding RNAs (ncRNAs) are generated from the larger part of the genome that does not encode proteins but produces transcripts that regulate gene expression and protein function [28]. NcRNAs also have the potential to be superior to established protein-based biomarkers [29]; however, the use of these is still limited by a wide range of concentrations and difficult detection when there is low abundance [30].

In bovines, ncRNAs were reported as being regulators of adipose tissue deposition [31] and fertility traits [32,33]. In Nellore cattle, ncRNAs were associated with an intramuscular fat trait [34]. For the RFI trait, there are studies on the *Bos Indicus* Cattle [35] and *Bos taurus* breeds [36,37]. Furthermore, studies to detect non-coding RNAs for Nellore cattle are still limited.

Studying the transcriptome in liver tissue associated with the RFI trait allows us to observe the influence and complexity of gene network interactions. Furthermore, it allows us to understand the molecular mechanisms involved in more efficient animals. The aim of this study was to use RNA-seq data to identify the DEGs, specific biomarkers, non-coding RNAs detection and enriched biological processes associated with RFI of the liver in Nellore cattle in two genetic groups.

## 2. Materials and Methods

### 2.1. Sampling Method

In this study, the authors describe and compare the transcriptome from two different Nellore cattle genetic groups. Genetic Group 1 (G1) was from the Zebu Breeding Program, created at the Instituto de Zootecnia in 1976, with the aim of increasing the post-weaning weight of animals based on individual performances in weight gain tests. At the beginning of the program, the Nellore herd was divided into three selection lines: Control Nellore (NeC), Selection Nellore (NeS) and Traditional Nellore (NeT). We used animals from the NeT line, in which the animals had been chosen based on maximum selection differentials for the weight gain tests. For the evaluation of RFI, the animals were kept in individual pens for a minimum period of 70 days, following 28 days of adaptation, during weight gain tests in 2009. The RFI was calculated as the difference between the individual dry matter intake observed and that predicted by the regression model according to Koch et al. [9]. Briefly, the G1 were fed a diet with a roughage: concentrate ratio of 19:81, with 82% total digestible nutrients and 14% crude protein, which consisted of hay, corn, cottonseed, cottonseed meal, citrus pulp, and a mineral mixture. Details about the animal experiment can be found in Zorzi et al. [38]. From the animals with an initial mean age of 19 months and mean live weight of 369 kg that were submitted to the RFI test (*n* = 60), 24 extreme animals were selected based in the RFI phenotype, 12 more efficient animals (i.e., a low RFI (LRFI)) where the mean was −0.28 kg/day and 12 less efficient animals (i.e., a high RFI (HRFI)) where the mean was 0.45 kg/day. The LRFI and HRFI groups were significantly different (*t*-test < 0.05). The sires were slaughtered and samples from their liver tissue were collected immediately and stored in an RNA holder (BioAgency, São Paulo, Brazil) at −80 °C until an RNA extraction. The liver tissue samples (50–80 mg) were collected immediately after slaughter and stored in a 15 mL Falcon tube containing 5 mL of RNA holder (BioAgency, São Paulo, SP, Brazil). The samples were frozen at −80 °C for a subsequent total RNA extraction. For more details, see Fonseca et al. [18] and Zorzi et al. [38].

Genetic Group 2 (G2) was from the public RNA-Seq data generated by the Brazilian Agricultural Research Corporation—EMBRAPA, Brazil. The animals used in the EMBRAPA study were from the artificial insemination of commercial and purebred Nellore dams, derived from 18 sires chosen to represent the main genealogies based on the information of the principal summaries of Brazilian Associations and to represent the average price of semen in use by Brazilian beef cattle farmers. The RFI was estimated according to Koch et al. [9] from a population of 83 sires with an average weight of 382.5 kg and an initial mean age of 21 months. The adaptation period was approximately 28 days and the individual dry matter intake was measured for at least 70 days. Briefly, the G2 were fed with diets formulated to contain 40% dry matter in the form of corn silage: crude protein at 13.5% and with energy densities of 2.8. Details about the animal experiment can be found in Oliveira et al. [13]. After the RFI evaluation, 20 extreme animals were selected based on the BLUP estimates (*n* = 585) of their additive genetic merits, 10 more efficient animals (a low RFI (LRFI)), in which the mean was −0.68 kg/day, and 10 less efficient animals (a high RFI (HRFI)), in which the mean was 0.53 kg/day (*t*-test < 0.05). For more details, see Tizioto et al. [19].

The RNA-Seq data from the G1 were generated by the authors in partnership with the Instituto de Zootecnia. The total RNA was extracted using the RNeasy Lipid Tissue Mini Kit (Qiagen, Valencia, CA, USA) according to the manufacturer’s protocol. The purity of the extracted RNA was determined by evaluating the absorbance at 260, 280 and 230 nm using a NanoDrop 1000 spectrophotometer (Thermo Fisher Scientific, Santa Clara, CA, USA, 2007). The reference values were ratios of 1.8 to 2 and ≥2, respectively. The total RNA quality was tested in an Agilent 2100 Bioanalyzer (Agilent, Santa Clara, CA, USA, 2009) using the Agilent RNA 6000 Nano Kit (Agilent). Values of the RNA Integrity Number (RIN) equal or higher than 7.0 were classified as satisfactory. In addition, the absence of contamination of the samples with genomic DNA was confirmed in a Qubit^®^ 2.0 Fluorometer (Invitrogen, Carlsbad, CA, USA, 2010). The mRNA libraries for sequencing were prepared using the TruSeq RNA Sample Preparation Kit^®^ (Illumina, San Diego, CA, USA) according to the manufacturer’s protocol. Libraries were pooled to enable multiplexed sequencing and, on average, generated 25 million reads per sample. The RNA sequencing (RNA-Seq) was performed using a HiSeq 2500 System (Illumina^®^) that generated 100 bp paired-end reads.

The raw data of the G2 were chosen from the ENA repository (EMBL-EBI) under accession PRJEB7696. The total RNA from the liver tissue samples of 20 Nellore animals divergent for RFI were selected. The RNA-Seq was performed using the HiSeq 2000 System (Illumina^®^), and paired-end reads (2 × 100 bp) were produced. All the information about the methodology used can be found in Tizioto et al. [19].

### 2.2. Transcriptome Analyses

Analyses were performed separately for each genetic group, using the same pipeline. All the samples from G1 and G2 were compared using two groups: LRFI and HRFI groups.

The quality of the RNA-Seq reads (i.e., the quality indices, GC content, N content, length distributions, duplication, overrepresented sequences and K-mer content) were verified using the Fastqc software (v.0.11.4) [39]. The low quality reads (i.e., adapter sequence and reads containing poly-N) were taken from the raw data using Trimmomatic v.0.36 [40] with the following parameters: java -jar /usr/local/bin/trimmomatic-0.36.jar PE -phred33 <IN_R1.fastq.gz> <IN_R2.fastq.gz> <OUT_R1.fastq.gz> <OUT_R1_UN.fastq.gz> <OUT_R2.fastq.gz> <OUT_R2_UN.fastq.gz> LEADING:3 TRAILING:3 SLIDINGWINDOW:4:15 MINLEN:50.

All the downstream analysis was based on the trimmed data with high quality reads. HISAT2 v.2.0.5 [41] was used to align the paired-end trimmed reads to the bovine reference genome (ARS-UCD1.2; *Bos taurus*) deposited in the National Center for Biotechnology Information (NCBI) (https://www.ncbi.nlm.nih.gov/ (accessed on 10 February 2020). These read counts were carried out using the in-house script (Supplementary Figure S1) and Ensembl (Index of /pub/release-105/gtf/bos_taurus/ (ensembl.org (accessed on 10 February 2020) gene annotations.

The EdgeR and Limma packages available from the Bioconductor project were used for the estimation of normalized CPM reads (counts per million) and to identify differentially-expressed genes (DEGs) between the RFI groups. EdgeR was used to normalize the data and Limma software was used for statistical methods (i.e., lineal modeled and empirical Bayes), following the methodology by Law et al. [42]. We considered a gene to be differentially-expressed when the *p*-value ≤ 0.05. The *p*-value correction (via a FDR calculation) by multiple tests was not applied in this study since we used a less stringent approach on a gene by gene basis, and this allowed us to import much more informative lists and assess the genome-wide data used for functional enrichment analyses and pathway analyses.

### 2.3. Gene Set Enrichment Analysis

To investigate the biological processes and differentially-expressed molecular functions (i.e., an enrichment analysis), we used a functional annotation gene set enrichment analysis, with the GSEA software [43] using the MSigDB database v7.5. The Pathway Studio software v.10 (https://www.elsevier.com/es-es/solutions/pathway-studio-biological-research (accessed on 12 May 2020), Elsevier Inc., Rockville, MD, USA) was used to identify the main biological processes and pathways with database ResNet v11.

### 2.4. Non-Coding RNAs Analysis

An alignment of the paired-end trimmed reads were performed with the *Bos taurus* bovine reference genome ncRNAs file, downloaded from the Ensembl database: https://www.ensembl.org/Bos_taurus/Info/Index (accessed on 02 March 2021) through HISAT2 v.2.0.5 [41]. Only reads showing a MAPQ equal to 0 were filtered out in order to remove the noise related to multiple mapping sites. Counts for each non-coding RNA detected were obtained by using an in-house script (Supplementary Figure S1), keeping only those non-coding RNAs with a mean count value higher or equal to 25 mean reads per sample. The EdgeR and Limma packages available from the Bioconductor project were used for the estimation of normalized CPM reads and to identify the differentially-expressed genes (DEGs) between the RFI groups. EdgeR was used to normalize the data and the Limma software was used for the statistical methods (i.e., lineal modeled and empirical Bayes), following the methodology by Law et al. [42]. We considered a gene to be differentially-expressed when the *p*-value ≤ 0.05. The Pathway Studio software (Elsevier) was used on the ncRNAs detected, and a pathway analysis was applied on the target genes predicted by the software.

### 2.5. Prediction of Footprint Gene Profile

To obtain the RFI predictors or biomarkers, receiver operating characteristic curves (ROC) were generated. For the analysis, the genefilters package (Bioconductor) was used with the used function of “rowpAUCs”. The function has applied on the normalized values (CPMs) in the two genetic groups (independent). A statistical correction (FDR) was not applied. An area under the ROC curve of 0.5 represents a test with no discriminating ability (i.e., no better than chance), while an AUC of 1.0 represents a test with a perfect discrimination [44]. The AUC of the proposed test was estimated to be >0.70.

## 3. Results

### 3.1. Alignment Statistics

The alignment parameters pipelines were the same to genetic group 1 and genetic group 2. The mean number of paired-end reads per sample after filtering was approximately 19.4 million reads for the G1 and for the G2 they were 20 million reads. The trimmed reads were around 86% for the G1 and around 88% for the G2 mapped with a reference genome (Supplementary Table S1). The box plot containing the CPMs for each group, and the plot of principal component analysis (PCA) indicating differences in the expression of genes between the LRFI and HRFI groups are shown in Supplementary Figure S2. The distribution of quartiles on the box plot was consistent between the groups, indicating a high quality of the data. In addition, the medians were similar in the two groups, indicating that the level of the sequencing coverage permitted the identification of low-expressed genes.

### 3.2. Differential Expression Analysis of RNA Seq Data

**Figure 1:**
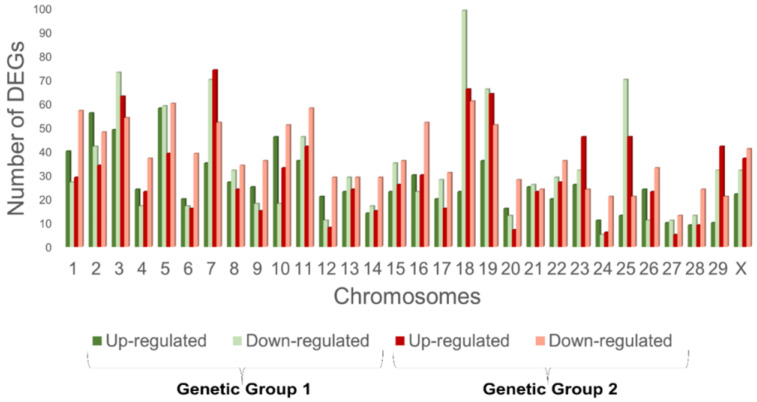
Distribution of the differentially-expressed genes across the chromosomes in G1 and G2.

Nellore animals from two genetic groups (i.e., G1 and G2), with each divided into two groups and divergent for RFI, were compared. The results from the analysis of the differential expression between the LRFI and HRFI (*p* ≤ 0.05) for G1 showed 1811 DEGs, of which 1007 were under-expressed, with a negative logFC value in the LRFI group (55.6%) and 804 were over-expressed, with a positive logFC value in the LRFI group (44.4%). Genetic group 2 showed 2054 DEGs, of which 1140 were under-expressed, with a negative logFC value in the LRFI group (55.5%) and 914 were over-expressed, with a positive logFC value in the LRFI group (44.5%). There were 88 common DEGs obtained for both the genetic groups with the same biological orientation (i.e., fold-change) (shown in Figure 1 and Supplementary Table S2).

### 3.3. Biomarkers

**Figure 2:**
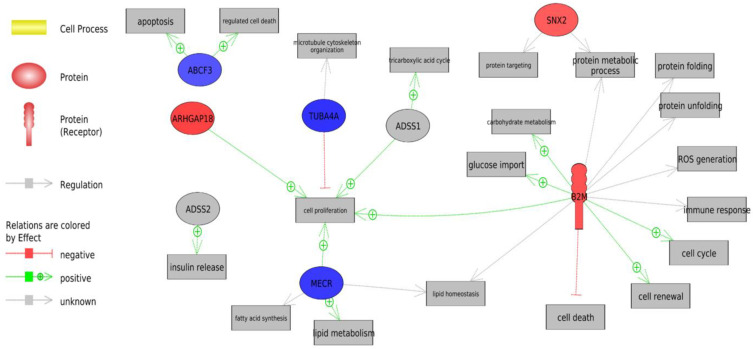
Biological processes associated with biomarkers related to the residual feed intake trait (RFI). The genes highlighted in red are up-regulated in low residual feed intake (LRFI) group, the blue circles present the down-regulated genes in the LRFI group compared with the high residual feed intake (HRFI) group; the green arrows indicate a positive regulation effect, while the red arrows denote a negative regulation effect.

To find the most important biomarkers that describe our trait, we performed ROC curves using an AUC > 0.70. We found seven genes: B2M, ADSS, SNX2, TUBA4A, ARHGAP18, MECR, and ABCF3 that were on our list of the 88 DEGs, and the changes in the expression and significance can be observed in Supplementary Table S2. These biomarkers were associated with important biological processes, such as cell proliferation, insulin release, apoptosis, regulated cell death, lipid metabolism, fatty acid synthesis, lipid homeostasis, protein targeting, protein metabolic process, protein folding, glucose import, carbohydrate metabolism, reactive oxygen species generation, immune response, cell cycle, cell renewal and cell death (Figure 2).

### 3.4. Gene Set Enrichment Analysis

We followed two strategies to address the biological analysis, one using all transcripts with a significant fold-change for each genetic group and the other filtering the 88 DEGs common in the two genetic groups.

#### 3.4.1. Biological Analysis of the Whole Transcriptome Data

The gene enrichment analysis (adjusted *p*-value < 0.05) showed 1275 biological processes in G1 (1163 with an up-regulation and 112 with a down-regulation), 124 molecular functions (95 up-regulated and 29 down-regulated), and 200 cellular components (148 up-regulated and 52 down-regulated), when comparing the LRFI and HRFI. For G2, 375 biological processes were detected (214 with an up-regulation and 161 with a down-regulation), 65 molecular functions (38 with a down-regulation and 27 with an up-regulation) and 90 cellular components (38 with a down-regulation and 52 with an up-regulation) when comparing the LRFI and HRFI (Supplementary Table S3).

Out of all the biological processes we highlighted, the ones related to energy metabolism, protein turnover, redox homeostasis and the immune system were all common to G1 (Supplementary Table S4) and G2 (Supplementary Table S5). These biological processes were described previously in the analysis of biomarkers (Figure 2).

The following describes each general biological process found and studied for each genetic group:

##### Energy Metabolism

Biological processes were identified for G1 related to a negative energy balance in more efficient cattle such as a negative regulation of energy derivation by the oxidation of organic compounds (GO:0015980) through body use to produce energy and the adenosine triphosphate (ATP) metabolic process (GO:0046034), and furthermore, in the liver synthesis of lipids, lipoproteins or phospholipids which are then used in the rest of the tissues of the organism. However, in the LRFI group, an inhibited lipoprotein biosynthetic process was found (GO:0042158), as was the lipoprotein metabolic process (GO:0042157), namely, the oligosaccharide-lipid intermediate biosynthetic process (GO:0006490), and there may have been less lipid accumulation in the more efficient group (LRFI). On the other hand, in the LRFI group, we saw a regulation of phospholipase activity (GO:0010517), a regulation of lipid kinase activity (GO:0043550) and a positive regulation of lipid kinase activity (GO:0090218).

This tendency was also observed for G2 where we found, in the LRFI animals, an inactive regulation of the generation of precursor metabolites and energy (GO:0043467), even though we observed that a fatty acid derivative biosynthetic process was activated (GO:1901570), and the lipopolysaccharide-mediated signaling pathway (GO:0031663).

The liver regulates glucose in the blood, converting glucose to glycogen or triacylglycerides or converting glycogen to glucose to elevate its level. When there is a lack of glucose and glycogen, the liver can convert amino acids and lipids to glucose. In the more efficient group (LRFI), an inactive glycogen biosynthetic process (GO:0005978), a glucan biosynthetic process (GO:0009250), a regulation of the glycolytic process (GO:0006110), and the cellular response to glucose starvation (GO:0042149) could be observed.

##### Protein Turnover

The renewal of proteins (protein turnover) is related to both the synthesis of new proteins and the degradation of already existing proteins that provide essential components to synthesize other proteins.

The LRFI group of the G1 displayed tyrosine phosphorylation of STAT (GO:0007260) protein processes, a regulation of protein serine/threonine kinase activity (GO:0071900), protein autophosphorylation (GO:0046777), an activation of protein kinase activity (GO:0032147), a positive regulation of protein-containing complex assembly (GO:0031334), a positive regulation of cellular protein localization (GO:1903829), a negative regulation of protein phosphorylation (GO:0001933), and the regulation of protein binding (GO:0043393).

The LRFI group of G2 exhibited processes such as a positive regulation of peptidase activity (GO:0010952), and SRP-dependent co-translational protein targeting to a membrane (GO:0006614) in conjunction with essential proteins to carry out the translation in the ribosome, while we also found an inactivated amino-acid betaine metabolic process (GO:0006577) in the LRFI.

##### Redox Homeostasis

Various biological processes stood out in both genetic groups that were related to an increase in the oxidative processes in less efficient bovine. Several biological processes related to the mitochondrial function were observed in G1: a mitochondrial respiratory chain complex I assembly (GO:0032981), a mitochondrial respiratory chain complex assembly (GO:0033108), mitochondrial ATP synthesis coupled with proton transport (GO:0042776), a respiratory chain complex IV assembly (GO:0008535), and a respiratory electron transport chain (GO:0022904) in the LRFI group. In addition, we found that the Wnt signaling pathway (GO:0016055) was activated in the LRFI group. The Wnt controls multiple processes of development, cell proliferation and apoptosis [45].

We highlight several antioxidant processes in G2 in the LRFI group, such as mitochondrial electron transport, NADH to ubiquinone (GO:0006120), ATP synthesis-coupled electron transport (GO:0042773), the glutathione derivative metabolic process (GO:1901685), the glutathione derivative biosynthetic process (GO:1901687), cellular detoxification (GO:1990748) and a cellular response to toxic substances (GO:0097237). It is of utmost importance to maintain enough liver antioxidants given the large number of oxidation compounds in the liver that can trigger inflammation. In the LRFI group, there was a positive regulation of cysteine-type endopeptidase activity (GO:2001056), which was involved in the apoptotic process of the superoxide metabolic process.

##### Immune System

Additionally, hepatocytes are responsible for the synthesis of various enzymes and proteins with relevant functions in the immune system. Their main tasks are to ensure the peripheral immunotolerance of the organism with the help of hematopoietic cells and transforming growth factor-β. The liver participates in determining the shape of the immune response [46]. We observed the activation of an immune response (GO:0002253) and an adaptive immune response (GO:0002250) in both G1 and G2.

#### 3.4.2. Biological Analysis of Differentially-Expressed Genes

Here we present the biological analysis aimed at detecting the signaling pathways involving the 88 genes detected between both genetic groups showing the relevant biological processes. The results from the biological analysis showed processes that had already been observed in the analysis of the entire transcriptome, such as an immune response (*p*-value 3.4 × 10^−2^) and an actin cytoskeleton reorganization (*p*-value 1.7 × 10^−2^) (Table 1).

**Figure 3:**
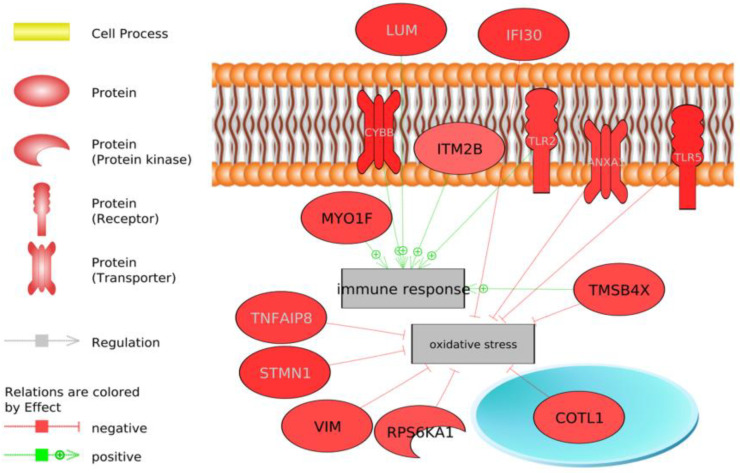
DEGs involved in the immune response and oxidative stress identified using Pathway Studio software. Highlighted in red are genes over-expressed in the LRFI group and in blue are under-expressed genes in the LRFI group. Green arrows indicate a positive regulation effect, while red arrows indicate a negative regulation effect.

**Figure 4:**
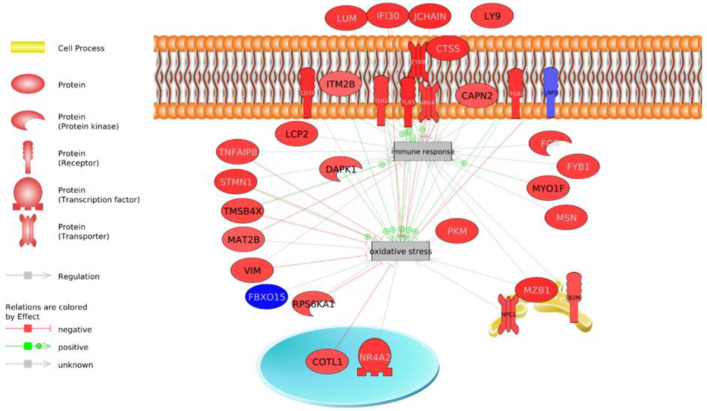
DEGs involved in the immune response and oxidative stress with an effect known using Pathway Studio software. Highlighted in red are genes over-expressed in the LRFI group and in blue are under-expressed genes in the LRFI group. Green arrows indicate a known positive regulation effect, red arrows indicate a known negative regulation effect and gray arrows indicate an unknown regulation effect.

**Figure 5:**
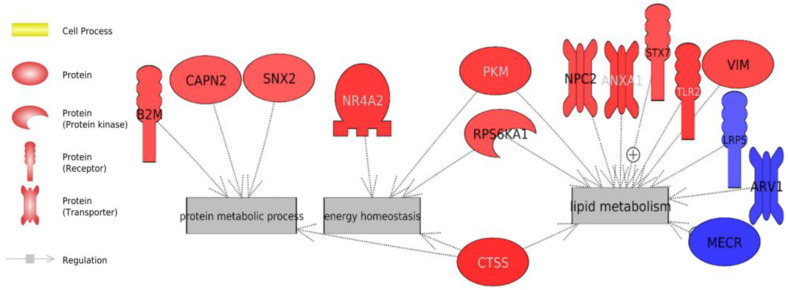
DEGs involved in protein metabolism, energy homeostasis and lipid metabolism using Pathway Studio software. Highlighted in red are genes over-expressed in the LRFI group and in blue are under-expressed genes in the LRFI group. Gray arrows indicate an unknown regulation effect. Previously, in Figure 3, we showed 33 genes involved in the immune response and oxidative stress, i.e., almost 38% of the total 88 DEGs.

In addition, the analysis delved into the genes that were integrated into the general processes highlighted in the analysis of all transcriptomes: energy homeostasis and lipid metabolism, the protein metabolic process, oxidative stress and the immune system (Figure 3, Figure 4 and Figure 5).

We have potentially identified genes involved in antioxidant mechanisms that play a key role in hepatic metabolic adaptation by inhibiting oxidative stress, such as *TMSB4X*, *COTL1*, *RPS6KA1*, *VIM*, *STMN1*, *TNFAIP3*, *IFI30*, *ANXA1*, *TLR5* and others by activating the immune system, such as *MYO1F*, *ITM2B*, *CYBB*, *TLR2* and *LUM* (Figure 4).

### 3.5. Analysis of Non-Coding RNAs

A transcriptome analysis was performed to investigate ncRNAs. For G1, there were 226 non-coding RNAs and 49 non-coding RNAs for G2. Applying the filter (i.e., 25 mean count/samples), there were 14 for G1 and 4 for G2. A differential analysis without a filter found two significant non-coding RNAs in the LRFI group for G1: small nucleolar RNA, namely, C/D box 16 (SNORD16) and microRNA 25 (MIR25). For G2, we found two significant non-coding RNAs in the LRFI group; small Cajal body-specific RNA 10 (SCARNA10), a nuclear-retained long non-coding RNA, and RMRP (RNase_MRP), a long non-coding RNA (Table 2).

**Figure 6:**
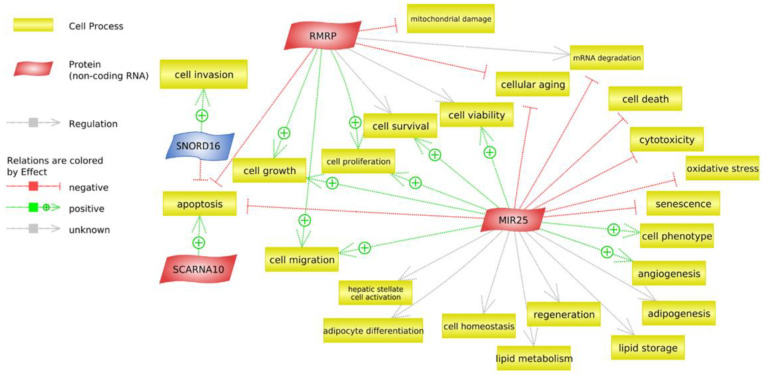
The main cellular processes for non-coding differentially-expressed RNAs associated with the RFI trait using the Pathway Studio software. The cellular processes are highlighted in yellow rectangles. The non-coding RNAs highlighted in red are up-regulated in the LRFI, the blue rectangles present the down-regulated non-coding RNA in the LRFI group compared with the HRFI; green arrows indicate a positive regulation effect, while red arrows indicate a negative regulation effect and gray arrows indicate an unknown regulation effect.

We now show the cellular processes for all the differentially-expressed non-coding RNAs found in the G1 and G2 in the LRFI (Figure 6). For G1, the MIR25 acts by blocking oxidative stress, cell death, and toxicity and by activating cell survival, angiogenesis and cell proliferation, among others. The SNORD16 acts by blocking cell invasion, although it also activates apoptosis. For G2, RMRP was associated with important cellular processes such as the inactivation of mitochondrial damage, apoptosis, cellular aging and the activation of cell growth and cell proliferation. The SCARNA10 acts by activating apoptosis.

## 4. Discussion

There is a clear lack of similarity of the key genes that contribute to RFI in the literature. Despite this, our work stands out for being the first in the literature to compare the gene expression of two divergent genetic groups for RFI, in the male Nellore breed with a similar age and weight, and the first to find common genes. The lines compared (i.e., G1 and G2) have different genetic origins and are from two important and independent researcher institutes in Brazil, which aim to enable sustainable technological solutions for the cattle production chain for the benefit of Brazilian society.

Despite the difference between the lines studied, common genes (*n* = 88) for RFI were found, indicating the discovery of new biomarkers that can be used for improving feed efficiency in the Brazilian herd. In addition, the observation of the biological function of all genes that exhibited changes in expression can give a more realistic view of the influence of the RFI trait in Nellore cattle.

Although undoubtedly due to the quantitative nature of the RFI, the environmental factors, such as the farm, production management and feeding, thus contributed to differences in the transcriptome between the genetic groups. We believe that the few genes found to be common between the groups further enrich the results found in our work, since they appear to be even more relevant for the RFI trait, as they influence its expression in Nellore animals, despite differences in management and genetic lines.

RNA-seq analysis is an exploratory approach that provides new hypotheses to be investigated by other complementary approaches. This helps to contribute to genomic selection programs to improve feed efficiency in beef cattle. Therefore, even though there are other studies that have provided information on RFI regulation through the liver physiology, the biological processes that govern differences in feed efficiency are not fully elucidated and more research is needed.

The results of this study may help to clarify the biological processes involved in the liver of Nellore cattle. Steers with a low RFI exhibited a greater feed efficiency as they use energy to increase protein synthesis and turnover to the detriment of glucose and lipid utilization. In addition, a decrease in oxidative stress was observed in the LRFI groups, which confers a greater liver protection against infections and pathologies and a better immune response. Moreover, an increase in antioxidant capacity and strength, a greater cell proliferation capacity and protection against cell death were observed in the efficient group. Important biological processes were observed that explain the differences between groups with divergent RFI phenotypes.

### 4.1. Energy Metabolism

We observed the participation of several biological processes related to mitochondrial and lipid metabolism in G1 and G2 (Supplementary Tables S4 and S5). Hepatocytes have a great metabolic capacity contributing to a variation in energy utilization. They generate 90% of the energy produced in the body, mainly in the mitochondria [47]. Furthermore, they are responsible for metabolizing lipids, proteins and carbohydrates into biologically-useful molecules [48,49]. Bottje et al. [50] were the first to demonstrate that energy metabolism could be a true determinant of variation in feed efficiency. It is estimated that two-thirds of RFI variation is due to differences in the resting energy expenditure [51]. Moreover, LRFI cattle consume 20% less food for the same level of yield [52,53]. The variation in the total energy expenditure of cattle of the same breed and of a similar management may be due to differences in the physiological processes, such as lipid metabolism [54].

Both G1 and G2 presented a down-regulation of the activation of the MAPKK pathway activity (see Additional Table 2 and Table S3) in the LRFI group. The MAPK pathway regulates food consumption and energy expenditure [55,56]. The inactivation in our more efficient group could have resulted in an inhibition of gluconeogenesis [57] and lipogenesis [58] which could have favored fewer lipids being stored or formed in the liver, because a constant activation of the MAKPP pathway causes fat accumulation [59].

The animals in the HRFI group could, consequently, have had an increased fat accumulation in hepatic cells, which could have led to the development of fatty liver [60], thereby exposing the animals to several metabolic stressors [61]. In addition, we observed a negative trend for lipid synthesis in the LRFI animals which may have been caused by processes that increased the flow and hepatic uptake of fatty acids and/or altered its metabolism (e.g., synthesis, oxidation or esterification) [62] (Figure 5). Our results are consistent with different studies of the liver transcriptome in bovine that dictate the great importance of lipid regulation for the RFI trait [63,64], as well as those of a study on pig liver [65].

### 4.2. Protein Turnover

We observed a trend toward protein synthesis and turnover in the LRFI groups (Figure 5, Supplementary Tables S3 and S4) as other studies have already reported [66,67]. This suggests that feed-efficient animals divert the energy or nutrients consumed toward protein synthesis. This feature may give feed-efficient animals a great metabolic advantage over less efficient animals. Our results show a modulation in actin cytoskeleton reorganization (Supplementary Tables S4 and S5 and Table 1), thereby contributing to the muscle regeneration that is usually accompanied by proliferation and fusion into myotubes [68]. It has been observed that a higher protein turnover results in a decrease in oxidative stress and a better efficiency in the use of energy in steers [69].

### 4.3. Redox Homeostasis

Oxidative stress is caused by an imbalance between the production of reactive oxygen species (ROS) and the antioxidant capacity of a cell. A malfunction of these mechanisms can result in excess ROS production or a decrease in ROS reduction capacity, which in turn can cause lipid peroxidation and apoptosis [70]. In addition, ROS generated by mitochondria play an important role in the regulation of other proapoptotic proteins [71]. Mitochondria are intrinsically associated with oxidative stress and cell signaling which are responsible for maintaining homeostasis and survival [72,73,74,75].

The more efficient RFI group (LRFI) would have been more protected against oxidative stress since many genes involved in its inhibition were observed (Figure 4), and they would be less likely to have had liver lesions as reported by a study on Nellore cattle [63]. In the same way, a better ability to modulate oxidative stress was observed in the more efficient animals [19,76] and a decreased respiratory capacity and increased ROS production in the HRFI animals [77,78,79]. Previous studies have suggested a link between the mitochondrial function and feed efficiency in broilers [50,77]; however, few studies have reported this link in beef cattle, especially in skeletal muscle tissue [18,80] and liver tissue [18,69,80].

This result is corroborated by the finding of an over-expression of genes common to the genetic groups that are involved in the reduction of oxidative stress and that may act as antioxidants in LRFI animals: *TMSB4X*, *COTL1*, *RPS6KA1*, *VIM*, *STMN1*, *TNFAIP3*, *IFI30, ANXA1*, and *TLR5* (Figure 5).

Antioxidants are molecules that slow down or inhibit the oxidation of diverse species and oxidative forms capable of attacking the organism, and these include glutathione peroxidase (GPx) and glutathione (GSH) among others [81]. Our results demonstrate that the LRFI group experienced an increase in antioxidants such as glutathione (Supplementary Table S5). The glutathione family has previously been implicated in food efficiency in several species [66,82,83,84]. Furthermore, the participation of the Wnt pathway in the LRFI group was identified (Supplementary Table S4); this pathway acts as an antioxidant, anti-inflammatory, antitumor and hepatoprotective agent [85] and controls multiple development processes, such as apoptosis. Its dysregulation has been observed in various pathologies [45,86].

Our study showed potential biomarkers in the LRFI and HRFI groups (Figure 2 and Figure 5), highlighting *B2M* as the biomarker involved in most of the biological processes. *B2M* was over-expressed in the LRFI animals when compared to the inefficient group. It has been identified in more efficient pigs, thus relating it to lipid metabolism [87]. Furthermore, it plays a key role in lipid homeostasis, regulating its downstream target genes, such as FAS, and an accumulation of fatty acids and lipid droplets [88]. *B2M* reduces the secretion of lipid binding protein-2 [89] and it also participates in protein turnover [90]. This gene regulates processes such as the inhibition of cell death [91]. Moreover, it seems that its over-expression could help to reduce infections [92], as it plays an important role in the regulation of the immune system by binding to T cells and other immune receptors [93]. Additionally, it has been described as a biomarker of kidney filtration and increased cell turnover [94], as has another biomarker, *SNX2* (sorting nexin 2) (Figure 2) [95].

### 4.4. Immune Response

Genetic group 1 showed a signaling pathway receptor activation via STAT that could have come from the activation transforming growth factor beta production, subsequently activating the ERK1 and ERK2 cascade, resulting in greater cell proliferation [96]. Growth factor pathways such as IGF-1 have already been described as biomarkers of food efficiency [97], whose function is linked to the immune system and tissue repair, among others, [98]. In addition, the ERK 1 and 2 pathways are related to immunity regulation, stress response, survival, and apoptosis [99]. Cytokines also activate the ERK 1 and 2 pathways and the phosphorylation of threonine appears to be necessary to induce the synthesis of these cytokines [100]. We found the modulation of this route (i.e., peptidyl-threonine phosphorylation) as well as the production of cytokines (Supplementary Tables S4 and S5) in the two genetic groups. Moreover, this was corroborated by the analysis of the biological processes of the genes common to both genetic groups (Table 1 and Figure 3 and Figure 4). An activation of NF-κB was observed in the LRFI of both genetic groups (Supplementary Tables S4 and S5), which may play an important role in immune responses and stress awhile retaining its impact in processes such as apoptosis, proliferation, differentiation and development [101].

The liver has potential functions in controlling the variation in feed efficiency [102], but little has been said about its immunological properties [103]. The liver is involved in innate immune defense and the regulation of liver regeneration [104]. The immune system uses a significant amount of energy, produces metabolic heat and increases oxygen consumption by 20–30% [105]. Energy metabolism is strongly related to productivity traits and the immune response could regulate energy metabolism [106]. In cattle, for example, there are several studies on the RFI-divergent immune system [107,108].

More efficient cattle may have a better response to inflammation and they may expend less energy fighting pathogens than heifers with a high RFI, while they also respond differently to pro-inflammatory hepatic stimuli and can have more energy available for muscle growth and deposition [108]. Furthermore, in humans, it was seen that a high immune response had a greater average daily weight gain compared to people with a low immune response [109]. In pigs, a connection between the various hepatic inflammatory responses was observed in animals with a high versus a low feed efficiency [110], the same as in cattle [63,108]. A rapid response to hepatic pro-inflammatory stimuli may result in a lower energy consumption and, therefore, a more efficient utilization of nutrients for growth and protein accumulation [108].

However, the immune response is said to be an energetically-costly process, which generates fewer nutrients available for growth [111] and can have a negative impact on the feed efficiency of the animal. In pigs, the genetic selection for LRFI has been shown to reduce the total digestive capacity of the growing tract during immune stimulation. [112]. In chickens, an inflammatory challenge tends to decrease the protein synthesis rates in skeletal muscles [113], although another study found that selection did not cause differences in the energy partition but rather increased the energy required for maintenance [114]. Therefore, the relationship between production and immunity in cattle is still unknown, as there are few studies that have investigated this topic. Further research is needed to elucidate the trade-offs between immunity and the RFI trait.

### 4.5. Non-Coding RNA

The central dogma of molecular biology states that RNA functions revolve around protein translation, namely, the messenger RNAs (mRNAs), as temporary copies of genetic information, and ribosomal RNAs (rRNAs), as a main component of ribosome, or translators of the codon sequence (tRNAs). It is now known that this process represents less than 2% of the genome, and this insufficiently explains the functionality of 98% of the RNA transcripts [115]. The ENCODE (Encyclopedia of DNA Elements) project was launched in 2005 and its recent reports revealed that up to 80% of the human genome has the capacity to transcribe into ncRNAs [116,117]. Currently, ncRNAs can be defined by length—small 18–200 nts and long >200 nts, and their regulatory functionality, such as the microRNAs (miRNAs), small nuclear RNAs (SnoRNAs) and long non-coding RNAs (lncRNAs) [118].

MicroRNAs act by regulating the expression of genes that drive numerous cellular processes. MIR25 is involved in biological processes such as the DNA damage response, cell cycle regulation, cell proliferation, migration and differentiation. In addition, many MIR25 target molecules can be found among the extracellular matrix components and membrane receptors [119]. It also has a role in the modulation of oxidative stress by targeting NADPH oxidase 4 [120]. Its over-expression protects cardiomyocytes against oxidative damage by inactivating the mitochondrial apoptosis pathway [121,122,123]. Additionally, MIR25 can decrease p53 levels, thus impairing the downstream p53 effects including senescence, apoptosis and cell cycle arrest [124]. Moreover, MIR25 acts as a negative feedback anti-fibrotic control during hepatic stellate cell activation by reducing the reactivity of hepatic stellate cells to a TGF-β-induced collagen expression, and modulating the cross-talk between Notch, Wnt and TGF-β signaling [125]. Therefore, this gene could regulate mitochondrial functions in animals with a low RFI. Several other studies have also associated mitochondrial function with RFI in cattle, including the Nellore breed [18,69,126]. In bovine, the MIR25 was also related to the fertility trait [127,128].

The other small non-coding RNAs observed were SNORD16, which are down-regulated in LRFI. SnoRNAs are predominately found in the nucleolus, and mainly function as guide RNAs for the post-transcriptional modification of ribosomal RNAs. The SNORD16 are categorized according to the modification type as box H/ACA snoRNAs and are associated with rRNA pseudouridylation [129]. The over-expression of snoRNAs plays a critical role in the onset and development of diseases, especially cancer [130,131]. This non-coding may help the immune system response to avoid diseases or assist when confronted by dysregulation of the immune system.

Long non-coding RNA (lncRNA) are thought to play a role in controlling gene expression in livestock species [132]. Furthermore, lncRNAs are considered evolutionarily less-conserved than protein-coding genes, which does not indicate a lack of function, but rather a possible fast adaptation mechanism [133]. LncRNAs associated with metabolic efficiency were classified to be functionally-involved in hepatic amino acid metabolism and protein synthesis in skeletal muscle cells in cattle [134], while in indicus cattle liver lncRNAs involved with lipid homeostasis and immune responses were detected [35]. LncRNAs have been suggested as therapeutic targets for metabolic diseases because of their involvement in lipid metabolism, adipogenesis and fat deposition [135]. Few studies, however, have attempted to catalogue lncRNAs in bovine, particularly in indicine cattle [136].

SCARNA10 is a lncRNA transcript detected in G2. Hepatic fibrosis is characterized by the accumulation of excessive amounts of extracellular matrix components in the liver. This gene could be relevant in liver fibrosis [137]; however, in our model, it may have been regulating in some way in the extracellular matrix in the LRFI group.

The last detected long non-coding was RNase_MRP l in G2, which is an RNA component of mitochondrial RNA processing endoribonuclease (RMRP). RMRP is a recognized non-coding transcript [138] which inhibits lipopolysaccharide-induced apoptosis of cardiomyocytes and mitochondrial damage by suppressing the post-transcriptional regulatory function of miR-1-5p on HSPA4. For example, RMRP lncRNA prevented mitochondrial dysfunction and cardiomyocyte apoptosis via the miR-1-5p/hsp70 axis in a sepsis model mouse [139]. Thus, RMRP gene mutations can lead to decreased cell growth by impairing the ribosomal assembly and altering the cyclin-dependent cell-cycle regulation. An over-expression of RMRP in human fibroblasts, for example, markedly elevated the production of cleaved or processed 5.8S ribosomal RNA and, consequently, accelerated cellular growth rates [140]. In addition, RMRP inhibition attenuated lipid accumulation [141]. This gene could have been involved in mitochondrial functions and in ribosomal assembly as well as helping the lipid accumulation in the LRFI group.

## 5. Conclusions

Eighty-eight common DEGs were identified in the two genetic groups. The biomarkers relevant to our trait in liver tissue were identified as B2M, ADSS, SNX2, TUBA4A, ARHGAP18, MECR, and ABCF3, with the over-expressed B2M gene participating in most cellular processes that differentiated the groups with a greater or lesser food efficiency. The biological pathways associated with RFI of the liver in Nellore cattle in the two genetic groups were energy metabolism, protein turnover, redox homeostasis and the immune response. The common transcripts, biomarkers and metabolic pathways found in the two genetic groups make this unprecedented work even more relevant, since the results are valid for different herds raised and slaughtered in different ways. We found up-regulated MIR25 and down-regulated SNORD16 non-coding RNAs in the RLFI for G1. G2 exhibited up-regulated RNase_MRP and SCARNA10. We highlight MIR25 which can act by blocking cytotoxicity and oxidative stress and RMRP as a blocker of mitochondrial damage. These results reinforce the biological importance of these known processes but also reveal new insights into the complexity of the liver tissue transcriptome of Nellore cattle.

## Figures and Tables

**Table 1:** The biological processes based on an enrichment *p*-value ≤ 0.05 involving the 88 genes detected between both genetic groups.

Go Term	Biological Processes	Genes	*p*-Value
GO:0071803	positive regulation of podosome assembly	*ARGEF5*, *LCP1*, *MSN*	8.0 × 10^−4^
GO:0045087	innate immune response	*FGR*, *ANXA1*, *B2M*, *CYBB*, *JCHAIN*, *LYA*, *TLR2*, *TLR5*	2.4 × 10^−3^
GO:0051493	regulation of cytoskeleton organization	*ARGEF5*, *CAPN2*, *STMN1*	3.0 × 10^−3^
GO:0008360	regulation of cell shape	*FGR*, *ARHGAP18*, *ANXA1*, *ITGB2*, *MSN*	3.1 × 10^−3^
GO:0001666	response to hypoxia	*CAPN2*, *NR4A2*, *PAK1*, *PKM*, *TLR2*	6.4 × 10^−3^
GO:0035556	intracellular signal transduction	*ARHGEP5*, *ADRAIA*, *ASB8*, *DAPK1*, *LCP1*, *ARS6KA1*, *STMN1*	7.6 × 10^−3^
GO:0032366	intracellular sterol transport	*ARV1*, *NPC2*	8.6 × 10^−3^
GO:0007409	axonogenesis	*CNTN4.LUM*, *PAK*, *STMN1*	8.6 × 10^−3^
GO:0007165	signal transduction	*CD74*, *ARHGAP30*, *ADRAIA*, *APBBIKP*, *AMXA1*, *DAPK1*, *GRN*, *MRC2*, *NR4A2*, *RPS6KA1*, *STMN1*, *TLR2*	1.0 × 10^−2^
GO:0006915	apoptotic process	*TNFAIP8*, *ADRAIA*, *DAPK1*, *ITGB2*, *MZB1*, *PAK1*, *RPRGKA1*, *TLR2*	1.1 × 10^−2^
GO:0001895	retina homeostasis	*ACTB*, *B2M*, *JCHAIN*	1.3 × 10^−2^
GO:0031532	actin cytoskeleton reorganization	*ANXA1*, *PAK1*, *PARVG*	1.7 × 10^−2^
GO:0006886	intracellular protein transport	*CD74*, *AP4B1*, *SNX2*, *SNX6*, *STX7*	1.9 × 10^−2^
GO:0034123	positive regulation of toll-like receptor signaling pathway	*TLR2*, *TLR5*	2.1 × 10^−2^
GO:0006897	endocytosis	*LRP5*, *MRC2*, *SNX2*, *SNX6*	2.2 × 10^−2^
GO:0098609	cell–cell adhesion	*ABI3*, *LRRFIPI*, *ARHGAP18*, *PKM*, *SNX2*	2.9 × 10^−2^
GO:0006955	immune response	*CD74*, *B2M*, *CTSS*, *JCHAIN*, *LCP2*, *TLR2*	3.4 × 10^−2^
GO:0022617	extracellular matrix disassembly	*CAPN2*, *CTSS*, *LCP1*	4.2 × 10^−2^
GO:0050707	regulation of cytokine secretion	*TLR2*, *TLR5*	4.6 × 10^−2^

**Table 2:** The differential analysis of non-coding RNAs in G1 and G2.

**Genetic group** **1**			
ID	Name	Fold change (log2) *	*p*-value
SNORD16	Small nucleolar RNA, C/D box 16	−0.356	0.01276
MIR25	MicroRNA 25	0.511	0.04741
**Genetic group 2**			
ID	Name	Fold-change (log2) *	*p*-value
SCARNA10	Small Cajal body-specific RNA 10	0.636	0.01855
RNase_MRP	RNA component of mitochondrial RNA processing endoribonuclease	0.664	0.02635

* The fold-change estimates (relative expression) refer to the LRFI group.

## Data Availability

The datasets for Genetic group 2 analyzed during the current study are available in the EMBL-EBI repository, under accession PRJEB769. The data for Genetic group 1 that support the findings of this study have belonged to commercial and experimental breeding programs, and restrictions are applied to the availability of these data, which were used under license for the current study, and so are not publicly available. However, the data are available by contacting the corresponding authors upon reasonable request and experimental breeding program (contacting the researcher maria.mercadante@sp.gov.br).

## Supplementary Materials

The following supporting information can be downloaded at: https://www.mdpi.com/article/10.3390/ani13030359/s1, Figure S1: The own script used for the reads count; Figure S2: Global statistics and quality control for both genetic group’s data. (A) box plot of CPMs distributions for individual conditions for genetic group 1 and genetic group 2 (C), color blue represents the LRFI group and color red represents the HRFI group. (B) PCA plot for genetic group 1 and genetic group 2 (D), color blue represents the LRFI group and color orange represents the HRFI group, Table S1: Descriptive statistics for alignment parameter using HITSAT2; Table S2: Eighty-eight common DEGs in G1 and G2 for LRFI (*p* ≤ 0.05); Table S3: The significant biological processes related to energy metabolism. Protein turnover, and redox homeostasis and immune system, in genetic group 1 in the most efficient group; Table S4: The significant biological processes related to energy metabolism. Protein turnover, and redox homeostasis and immune system in genetic group 2 in the most efficient group; Table S5. The significant biological processes related to Energy Metabolism. Protein turnover, and Redox homeostasis and immune system in genetic group 2 in the most efficient group.

## Abbreviations

Differentially-expressed genes (DEGs), Sustainable Development Goals (SDGs), residual feed intake (RFI), ribonucleic acid (RNA), low residual feed intake (LRFI), high residual feed intake (HRFI), counts per million (CPMs), false discovery rates (FDR), gene set enrichment analysis (GSEA), receiver operating characteristic (ROC), area under the curve (AUC), principal component analysis (PCA), logarithm of ratio (LOR), adenosine triphosphate (ATP), nicotinamide adenine dinucleotide (NADH), mitogen-activated protein kinase (MAPKK), reactive oxygen species (ROS), glutathione peroxidase (GPx), fas cell surface death receptor (FAS), signal transducer and activator of transcription (STAT), serine/threonine kinase 1(ERK1), serine/threonine kinase 2 (ERK1), insulin like growth factor 1 (IGF-1), nuclear factor kappa-light-chain-enhancer of activated B cells (NF-Κb).
