# Supplementary material for: Transcriptome Profiling of the Liver in Nellore Cattle Phenotypically Divergent for RFI in Two Genetic Groups
Source: Animals (Basel). 2023 Jan 20;13(3):359. doi: 10.3390/ani13030359 (PMC9913155; doi:10.3390/ani13030359)
Supplement: Supplementary file 1 [file animals-13-00359-s001.zip › Supplementary Figure S1.pdf]

Figure S1. The own script used for reads count.

1. Download from Biomart database the following fields Chromosome, start, end, gene (mart_export.txt)
2. Information was splitted by chromosome (this action will reduce the computation times when annotating)
3. cut -f1,2,3,4,5 OUT.sam > OUT.txt (Read_name, flag, chromosome, position, MAPQ)
3. Python script for annotation

```
import os
from job import*
gene_list = []
folders = os.listdir(MAP_FOLDER_RESULTS)
for folder in folders:
    inFile = MAP_FOLDER_RESULTS + '/' + folder + '/OUT.txt'
    infile = open(MAP_FOLDER_RESULTS + '/' + folder + '/OUT.txt', 'r')
    lineas = sum(1 for line in open(inFile))
    for i in range (0,lineas,1):
        content = infile.readline()
        col = content.strip().split('\t')
        if int(col[4]) >= 5:
            chr = col[2]
            pos = int(col[3])
            infile_chr = open( 'INDEX_FOLDER/' + chr + '.txt', 'r')
            content_chr = infile_chr.readlines()
            for line in content_chr:
                col = line.strip().split('\t')
                start = int(col[3])
                end = int(col[4])
                if pos >= start and pos <= end:
                    info = col[8].strip().split(';')
                    gene = info[2].strip().split(' ' )
                    gene = gene[1].replace('"','')
                    gene_list.append(gene)
            infile_chr.close()
uniq = list(set(gene_list))
outfile = open(MAP_FOLDER_RESULTS +  '/' + folder + '/conteos.txt', 'w')
for gene in uniq:
    n = gene_list.count(gene)
    outfile.write(gene + '\t' + str(n) + '\n')
outfile.close()
```
