# Supplementary material for: Transcriptome Profiling of the Liver in Nellore Cattle Phenotypically Divergent for RFI in Two Genetic Groups
Source: Animals (Basel). 2023 Jan 20;13(3):359. doi: 10.3390/ani13030359 (PMC9913155; doi:10.3390/ani13030359)

Figure S2. Global statistics and quality control for both genetic group data. (A) Box plot of CPMs distributions for individual conditions for genetic group 1 and genetic group 2 (C), color blue represents the LRFI group and color red represents the HRFI group. (B) PCA plot for genetic group 1 and genetic group 2 (D), color blue represents the LRFI group and color orange represents the HRFI group.

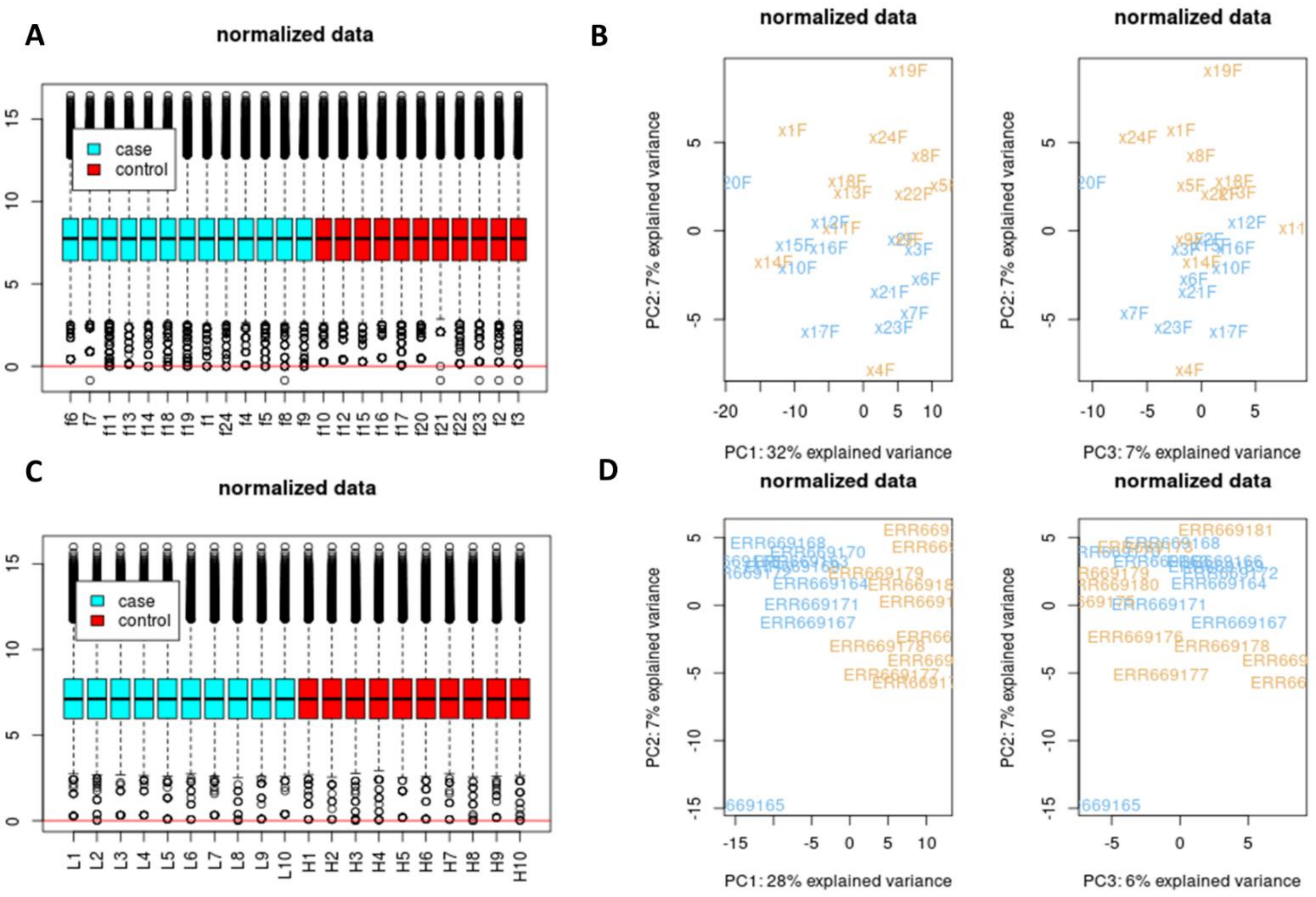

Supplement: Supplementary file 1 [file animals-13-00359-s001.zip › Supplementary Figure S2.pdf]
