# Supplementary material for: Transcriptome Profiling of the Liver in Nellore Cattle Phenotypically Divergent for RFI in Two Genetic Groups
Source: Animals (Basel). 2023 Jan 20;13(3):359. doi: 10.3390/ani13030359 (PMC9913155; doi:10.3390/ani13030359)
Supplement: Supplementary file 1 [file animals-13-00359-s001.zip › Supplementary Table S1.pdf]

Table S1. Descriptive statistics for alignment parameter using HITSAT2**.**

GENETIC GROUP 1			
**SEQUENCE ID**	Raw reads	Clean Reads (Mb)	Overall alignment rate (%)
**1**	23,225,642	19,014,864	81.87013302
**2**	20,051,086	17,212,955	85.84549984
**3**	18,988,306	15,889,039	83.67802267
**4**	20,205,500	17,574,112	86.97687263
**5**	18,829,397	16,264,153	86.37638794
**6**	20,146,026	17,381,814	86.27912026
**7**	18,249,944	16,186,137	88.69143379
**8**	19,573,711	16,880,873	86.24257812
**9**	17,903,628	15,736,760	87.89704522
**10**	19,769,426	17,087,279	86.43285344
**11**	17,534,365	15,482,048	88.29545866
**12**	21,066,221	15,814,760	75.07165144
**13**	22,230,830	18,936,800	85.18260452
**14**	20,087,642	17,272,061	85.98351663
**15**	17,484,201	15,082,220	86.26199161
**16**	20,735,282	18,109,819	87.33818522
**17**	18,991,037	15,699,968	82.6704092
**18**	16,449,275	14,561,444	88.5233179
**19**	20,211,361	17,472,382	86.44831983
**20**	20,560,712	17,973,022	87.41439499
**21**	17,944,043	15,322,488	85.39038833
**22**	17,679,765	15,634,492	88.43156003
**23**	17,659,388	15,043,701	85.18812203
**24**	19,262,155	16,550,940	85.92465381
**MEAN**	19,368,289	16,591,005	85.66066527
**GENETIC GROUP 2**			
**SEQUENCE ID**	Reads raw	Reads cleanined (Mb)	Overall alignment rate (%)
**1**	8,205,798	7,254,724	88.40973175
**2**	12,342,938	10,774,148	87.2899791
**3**	11,174,277	9,899,320	88.59025063
**4**	9,411,232	8,328,025	88.49027417
**5**	10,210,766	9,017,716	88.31576397
**6**	10,441,025	9,227,088	88.37339246
**7**	10,691,977	9,361,547	87.55674465
**8**	8,200,461	7,328,352	89.36512228
**9**	10,407,413	9,191,569	88.31751945
**10**	9,680,276	8,322,409	85.97284829
**11**	10,977,085	9,747,828	88.80160808
**12**	9,609,337	8,581,017	89.29874142
**13**	9,711,313	8,588,163	88.43462259
**14**	8,758,872	7,734,901	88.30932796
**15**	10,250,216	8,932,195	87.14152951
**16**	9,346,752	8,236,241	88.1187497
**17**	12,612,367	11,168,260	88.55007153
**18**	8,100,136	7,143,194	88.18609959
**19**	9,168,261	8,114,508	88.50651176
**20**	9,867,034	8,820,733	89.39599276
**MEAN**	19,916,754	17,577,194	88.25330751
