# Supplementary material for: Transcriptome Profiling of the Liver in Nellore Cattle Phenotypically Divergent for RFI in Two Genetic Groups
Source: Animals (Basel). 2023 Jan 20;13(3):359. doi: 10.3390/ani13030359 (PMC9913155; doi:10.3390/ani13030359)
Supplement: Supplementary file 1 [file animals-13-00359-s001.zip › Supplementary Table S2.pdf]

Table S2. Eighty-eight common DEGs in G1 and G2 for LRFI (p≤0.05).

		Genetic group 1			Genetic group 2		
**Gene Name**	**Gene Symbol**	**Fold change (log2)***	**p-value**	**padj**	**Fold change (log2)***	**p-value**	**padj**
**Ankyrin Repeat y SOCS Box Protein 8**	*ASB8*	-0.1495	0.0253	0.571	-0.1683	0.0056	0.308
**ATP Binding Cassette Subfamily F Member 3**	*ABCF3*	-0.1319	0.0412	0.571	-0.1681	0.0056	0.308
**Vascular cell adhesion molecule 1-like**	*LOC534578*	0.8064	0.0011	0.571	0.4097	0.0253	0.445
**Integral Membrane Protein 2B**	*ITM2B*	0.1688	0.0025	0.571	0.1242	0.0308	0.462
**Contactin 4**	*CNTN4*	0.9359	0.0066	0.571	0.6749	0.0472	0.51
**Pleckstrin Homology Domain Containing B2**	*PLEKHB2*	0.3896	0.0078	0.571	0.1984	0.0323	0.468
**Zinc finger protein 665**	*ZNF286A*	-0.2862	0.0085	0.571	-0.1821	0.0373	0.483
**Transmembrane And Coiled-Coil Domain Family 1**	*TMCC1*	-0.2	0.0093	0.571	-0.2596	0.0009	0.22
**Adenylosuccinate Synthase 2**	*ADSS*	0.276	0.0097	0.571	0.1704	0.0267	0.451
**Methionine Adenosyltransferase 2B**	*MAT2B*	0.2139	0.0097	0.571	0.1131	0.0435	0.5
**Major histocompatibility complex, class II, DR alpha Cyclin B3**	*BoLA-DRB3*	0.4489	0.0098	0.571	0.3182	0.0262	0.45
**Syntaxin 7**	*STX7*	0.2613	0.0106	0.571	0.1475	0.0479	0.51
**TNF Alpha Induced Protein 8**	*TNFAIP8*	0.3635	0.0107	0.571	0.2624	0.009	0.35
**F-Box Protein 15**	*FBXO15*	-0.4387	0.0108	0.571	-0.4727	0.0367	0.48
**LRR Binding FLII Interacting Protein 1**	*LRRFIP1*	0.327	0.0108	0.571	0.2132	0.0062	0.312
**Toll Like Receptor 5**	*TLR5*	0.5309	0.0112	0.571	0.8541	0.0159	0.394
**Joining Chain Of Multimeric IgA And IgM**	*JCHAIN*	0.5275	0.0124	0.571	0.3372	0.0146	0.387
**Uncharacterized**	*LOC515089*	-0.2801	0.0124	0.571	-0.3338	0.0054	0.306
**Cyclin B3**	*CCNB3*	-0.3393	0.0131	0.571	-0.437	0.0166	0.396
**Annexin A1**	*ANXA1*	0.3743	0.0136	0.571	0.2777	0.0171	0.401
**Inositol Polyphosphate-5-Phosphatase K**	*INPP5K*	-0.1223	0.0138	0.571	-0.1309	0.0405	0.494
**Adhesion G Protein-Coupled Receptor A3**	*ADGRA3*	-0.1803	0.0139	0.571	-0.1609	0.0074	0.333
**Zinc Finger CCHC-Type Containing 7**	*ZCCHC7*	-0.2154	0.0143	0.571	-0.2452	0.0201	0.425
**Lymphocyte Cytosolic Protein 1**	*LCP1*	0.5266	0.0145	0.571	0.4347	0.0078	0.338
**Major Histocompatibility Complex, Class II, DR Alpha**	*BoLA-DRA*	0.4832	0.0157	0.571	0.2185	0.0499	0.512
**CD74 Molecule**	*CD74*	0.5054	0.0158	0.571	0.2425	0.0213	0.432
**Transmembrane Protein 50A**	*TMEM50A*	0.1392	0.016	0.571	0.1724	0.0416	0.497
**Mannose Receptor C Type 2**	*MRC2*	0.5687	0.0169	0.571	0.3	0.0424	0.5
**Uncharacterized**	*LOC788175*	0.5953	0.0178	0.571	0.3008	0.027	0.451
**Adrenoceptor Alpha 1A**	*ADRA1A*	-0.2719	0.0193	0.571	-0.2767	0.0002	0.158
**Stathmin 1**	*STMN1*	0.4345	0.0194	0.571	0.2428	0.0382	0.487
**Helicase, Lymphoid Specific**	*HELLS*	0.4887	0.0205	0.571	0.4734	0.0255	0.446
**IFI30 Lysosomal Thiol Reductase**	*IFI30*	0.4426	0.0206	0.571	0.2357	0.0311	0.463

**Mitochondrial Trans-2-Enoyl-CoA Reductase Helicase, lymphoid-specific**	*MECR*	-0.2213	0.0217	0.571	-0.2052	0.0095	0.353
**DENN Domain Containing 10**	*FAM45A*	0.2852	0.0223	0.571	0.1354	0.027	0.451
**Sorting Nexin 2**	*SNX2*	0.214	0.0231	0.571	0.1328	0.0299	0.46
**Sorting nexin 6**	*SNX6*	0.1672	0.0234	0.571	0.1112	0.0456	0.508
**Rho Guanine Nucleotide Exchange Factor 5**	*ARHGEF5*	-0.2641	0.024	0.571	-0.2617	0.04	0.493
**Ribosomal Protein S6 Kinase A1**	*RPS6KA1*	0.252	0.025	0.571	0.252	0.025	0.571
**Protein Regulator Of Cytokinesis 1**	*PRC1*	0.4199	0.0252	0.571	0.1908	0.023	0.434
**Lumican**	*LUM*	0.4358	0.0263	0.571	0.3917	0.039	0.488
**Damage Specific DNA Binding Protein 2**	*DDB2*	0.1129	0.0264	0.571	0.2711	0.0081	0.342
**FYN Binding Protein 1**	*FYB1*	0.3626	0.0266	0.571	0.192	0.024	0.437
**Granulin Precursor**	*GRN*	0.1808	0.0271	0.571	0.1586	0.0036	0.282
**Pyruvate Kinase M1/2**	*PKM*	0.3742	0.0274	0.571	0.1707	0.0364	0.479
**Tetratricopeptide Repeat Domain 19**	*TTC19*	-0.1468	0.0278	0.571	-0.1329	0.0498	0.512
**ARV1 Homolog, Fatty Acid Homeostasis Modulator**	*ARV1*	-0.1859	0.0288	0.571	-0.175	0.0308	0.462
**BTB Domain Containing 6**	*BTBD6*	-0.169	0.0291	0.571	-0.0768	0.0388	0.488
**Vimentin**	*VIM*	0.3079	0.0291	0.571	0.3593	0.0085	0.342
**Uncharacterized**	*LOC783680*	0.2802	0.0297	0.571	0.1726	0.0247	0.441
**NPC Intracellular Cholesterol Transporter 2**	*NPC2*	0.2977	0.0297	0.571	0.3	0.0424	0.5
**Kelch Like Family Member 36**	*KLHL36*	-0.1304	0.0301	0.571	-0.1356	0.0355	0.474
**Lymphocyte Cytosolic Protein 2**	*LCP2*	0.3456	0.0302	0.571	0.2466	0.0003	0.161
**Rho GTPase Activating Protein 18**	*ARHGAP18*	0.3437	0.0304	0.571	0.2553	0.0292	0.458
**Calpain 2**	*CAPN2*	0.2296	0.031	0.571	0.1569	0.0408	0.495
**Thymus, Brain And Testes Associated**	*TBATA*	-0.4652	0.0319	0.571	-0.5368	0.0426	0.5
**Coactosin Like F-Actin Binding Protein 1**	*COTL1*	0.2718	0.0321	0.571	0.1888	0.0162	0.395
**LETM1 Domain Containing 1**	*LETMD1*	-0.1713	0.0335	0.571	-0.1957	0.0442	0.504
**Toll Like Receptor 2**	*TLR2*	0.3937	0.0337	0.571	0.3998	0.0008	0.204
**Cytochrome B-245 Beta Chain**	*CYBB*	0.5508	0.0338	0.571	0.4176	0.0248	0.443
**Amyloid Beta Precursor Protein Binding Family B Member 1 Interacting Protein**	*APBB1IP*	0.3322	0.034	0.571	0.2228	0.0061	0.31
**Uncharacterized**	*LOC112446800*	-0.5062	0.0343	0.571	-0.5978	0.0305	0.461
**P21 (RAC1) Activated Kinase 1**	*PAK1*	0.318	0.0344	0.571	0.2334	0.0356	0.474
**Thymosin Beta 4 X-Linked**	*TMSB4X*	0.3005	0.035	0.571	0.3199	0.0032	0.277
**Moesin**	*MSN*	0.3817	0.0356	0.571	0.1346	0.0447	0.506
**Nuclear Receptor Subfamily 4 Group A Member 2**	*NR4A2*	0.4061	0.0358	0.571	0.5458	0.0214	0.433
**Integrin Subunit Beta 2**	*ITGB2*	0.4358	0.0359	0.571	0.2014	0.0401	0.493
**Myosin IF**	*MYO1F*	0.3206	0.0366	0.571	0.1629	0.0369	0.482
**GDNF Inducible Zinc Finger Protein 1**	*GZF1*	-0.1394	0.0369	0.571	-0.2676	0.0003	0.161
**LDL Receptor Related Protein 5**	*LRP5*	-0.1151	0.038	0.571	-0.0944	0.0411	0.495
**Serine Palmitoyltransferase Small Subunit B**	*SPTSSB*	0.4723	0.04	0.571	0.5892	0.0184	0.413

**CD63 Molecule**	*CD63*	0.0771	0.0402	0.571	0.0907	0.0178	0.408
**Myosin Binding Protein H**	*MYBPH*	0.9146	0.0402	0.571	0.6306	0.0169	0.399
**Marginal Zone B And B1 Cell Specific Protein**	*MZB1*	0.4825	0.0407	0.571	0.57	0.0191	0.418
**Lymphocyte Antigen 9**	*LY9*	0.3472	0.0409	0.571	0.3123	0.0053	0.306
**SET Domain And Mariner Transposase Fusion Gene**	*SETMAR*	-0.2426	0.0409	0.571	-0.2787	0.017	0.399
**Cathepsin S**	*CTSS*	0.4946	0.041	0.571	0.3211	0.0209	0.428
**Ecotropic Viral Integration Site 2B**	*EVI2B*	0.2939	0.0427	0.571	0.1647	0.0454	0.508
**Solute Carrier Family 16 Member 9**	*SLC16A9*	0.5142	0.0431	0.571	0.5586	0.0058	0.308
**Parvin Gamma**	*PARVG*	0.3136	0.0454	0.571	0.2878	0.0205	0.427
**TATA-Box Binding Protein Associated Factor 5 Like**	*TAF5L*	-0.1487	0.0454	0.571	-0.1746	0.0458	0.508
**Beta-2-microglobulin**	*B2M*	0.2578	0.046	0.571	0.2057	0.0115	0.365
**Tubulin alpha 4a**	*TUBA4A*	-0.2453	0.0461	0.571	-0.2353	0.0178	0.408
**FGR Proto-Oncogene, Src Family Tyrosine Kinase**	*FGR*	0.3737	0.0462	0.571	0.202	0.0284	0.456
**Actin beta**	*ACTB*	0.2713	0.0466	0.571	0.1693	0.0346	0.472
**Rho GTPase Activating Protein 30**	*ARHGAP30*	0.299	0.0469	0.571	0.1761	0.0333	0.469
**Adaptor Related Protein Complex 4 Subunit Beta 1**	*AP4B1*	-0.2135	0.0481	0.571	-0.2169	0.0301	0.46
**Death Associated Protein Kinase 1**	*DAPK1*	0.2284	0.0482	0.571	0.2626	0.0192	0.418
