# Supplementary material for: Transcriptome Profiling of the Liver in Nellore Cattle Phenotypically Divergent for RFI in Two Genetic Groups
Source: Animals (Basel). 2023 Jan 20;13(3):359. doi: 10.3390/ani13030359 (PMC9913155; doi:10.3390/ani13030359)
Supplement: Supplementary file 1 [file animals-13-00359-s001.zip › Supplementary Table S3.pdf]

Table S3. The significant biological processes, molecular functions and cellular components in Genetic group 1and Genetic group 2 in the most efficient group.

**The significant biological processes in Genetic group 1 in the most efficient group.**					
**Go term**	**Number of genes**	**LOR***	**pvalue**	**padj**	**Biological process**
**GO:0140053**	161	-0.981	6.07E-38	3.51E-33	mitochondrial gene expression
**GO:0032543**	134	-1.021	3.16E-35	6.08E-31	mitochondrial translation
**GO:0070126**	89	-1.104	1.77E-34	2.56E-30	mitochondrial translational termination
**GO:0070125**	88	-1.093	2.74E-33	3.17E-29	mitochondrial translational elongation
**GO:0033108**	87	-1.039	3.46E-28	1.82E-24	mitochondrial respiratory chain complex assembly
**GO:0006415**	102	-1.001	5.55E-28	2.67E-24	translational termination
**GO:0034470**	350	-0.534	1.08E-22	2.07E-19	ncRNA processing
**GO:0010257**	58	-1.082	2.04E-22	3.59E-19	NADH dehydrogenase complex assembly
**GO:0032981**	58	-1.082	2.04E-22	3.59E-19	mitochondrial respiratory chain complex I assembly
**GO:0034660**	436	-0.458	5.36E-21	6.59E-18	ncRNA metabolic process
**GO:0006414**	138	-0.763	2.59E-19	2.42E-16	translational elongation
**GO:0008033**	123	-0.763	3.69E-17	2.77E-14	tRNA processing
**GO:0006399**	176	-0.630	8.62E-17	6.15E-14	tRNA metabolic process
**GO:0007005**	498	-0.374	4.61E-16	2.99E-13	mitochondrion organization
**GO:0006120**	46	-1.000	1.40E-14	7.84E-12	mitochondrial electron transport, NADH to ubiquinone
**GO:0009451**	149	-0.625	1.72E-14	9.45E-12	RNA modification
**GO:0042254**	279	-0.460	2.74E-14	1.44E-11	ribosome biogenesis
**GO:0022904**	100	-0.772	8.85E-14	4.41E-11	respiratory electron transport chain
**GO:0006400**	82	-0.782	2.02E-13	9.74E-11	tRNA modification
**GO:0006119**	121	-0.679	3.37E-13	1.58E-10	oxidative phosphorylation
**GO:0042773**	81	-0.833	1.18E-12	5.07E-10	ATP synthesis coupled electron transport
**GO:0022613**	405	-0.354	2.14E-12	8.97E-10	ribonucleoprotein complex biogenesis
**GO:0042775**	80	-0.823	2.60E-12	1.07E-09	mitochondrial ATP synthesis coupled electron transport
**GO:0022900**	164	-0.537	2.26E-11	8.17E-09	electron transport chain
**GO:0045333**	177	-0.506	4.25E-11	1.45E-08	cellular respiration
**GO:0016072**	212	-0.453	4.92E-11	1.64E-08	rRNA metabolic process
**GO:0006364**	202	-0.462	6.00E-11	1.97E-08	rRNA processing
**GO:0007007**	41	-0.917	1.52E-10	4.67E-08	inner mitochondrial membrane organization
**GO:0006520**	343	-0.335	8.63E-10	2.19E-07	cellular amino acid metabolic process

**GO:0015980**	271	-0.377	1.02E-09	2.54E-07	energy derivation by oxidation of organic compounds
**GO:1990542**	89	-0.645	1.36E-09	3.27E-07	mitochondrial transmembrane transport
**GO:0030150**	18	-1.155	6.31E-09	1.36E-06	protein import into mitochondrial matrix
**GO:0006839**	242	-0.377	7.86E-09	1.64E-06	mitochondrial transport
**GO:0006506**	31	-0.955	1.71E-08	3.31E-06	GPI anchor biosynthetic process
**GO:0006505**	32	-0.937	1.72E-08	3.32E-06	GPI anchor metabolic process
**GO:0006626**	96	-0.557	7.76E-08	1.27E-05	protein targeting to mitochondrion
**GO:0006744**	16	-1.097	8.66E-08	1.39E-05	ubiquinone biosynthetic process
**GO:1901663**	16	-1.097	8.66E-08	1.39E-05	quinone biosynthetic process
**GO:0044743**	33	-0.851	2.52E-07	3.58E-05	protein transmembrane import into intracellular organelle
**GO:0071806**	58	-0.650	6.54E-07	8.42E-05	protein transmembrane transport
**GO:0070131**	16	-1.070	9.17E-07	1.16E-04	positive regulation of mitochondrial translation
**GO:0065002**	48	-0.700	1.45E-06	1.74E-04	intracellular protein transmembrane transport
**GO:0043628**	34	-0.770	1.48E-06	1.76E-04	ncRNA 3'-end processing
**GO:0001522**	17	-1.070	1.59E-06	1.88E-04	pseudouridine synthesis
**GO:0015985**	20	-0.962	1.96E-06	2.26E-04	energy coupled proton transport, down electrochemical gradient
**GO:0015986**	20	-0.962	1.96E-06	2.26E-04	ATP synthesis coupled proton transport
**GO:1901605**	197	-0.340	2.21E-06	2.49E-04	alpha-amino acid metabolic process
**GO:0042158**	93	-0.497	2.40E-06	2.66E-04	lipoprotein biosynthetic process
**GO:0042407**	29	-0.800	2.73E-06	2.96E-04	cristae formation
**GO:0001510**	76	-0.533	2.73E-06	2.96E-04	RNA methylation
**GO:0009063**	122	-0.423	3.18E-06	3.39E-04	cellular amino acid catabolic process
**GO:0033617**	18	-0.981	3.46E-06	3.64E-04	mitochondrial cytochrome c oxidase assembly
**GO:0017004**	30	-0.826	3.86E-06	3.98E-04	cytochrome complex assembly
**GO:0016054**	268	-0.285	3.90E-06	4.00E-04	organic acid catabolic process
**GO:0046395**	268	-0.285	3.90E-06	4.00E-04	carboxylic acid catabolic process
**GO:0006497**	88	-0.490	6.23E-06	5.99E-04	protein lipidation
**GO:0000963**	17	-1.034	6.50E-06	6.24E-04	mitochondrial RNA processing
**GO:0042776**	19	-0.938	6.99E-06	6.62E-04	mitochondrial ATP synthesis coupled proton transport
**GO:0006457**	209	-0.313	7.72E-06	7.28E-04	protein folding
**GO:0000469**	22	-0.877	8.19E-06	7.63E-04	cleavage involved in rRNA processing
**GO:0016254**	17	-0.983	8.23E-06	7.64E-04	preassembly of GPI anchor in ER membrane

**GO:0000387**	38	-0.705	8.85E-06	8.11E-04	spliceosomal snRNP assembly
**GO:0016073**	31	-0.734	9.04E-06	8.27E-04	snRNA metabolic process
**GO:0030488**	38	-0.687	9.08E-06	8.29E-04	tRNA methylation
**GO:0008535**	21	-0.895	9.39E-06	8.52E-04	respiratory chain complex IV assembly
**GO:0097502**	33	-0.851	1.02E-05	9.13E-04	mannosylation
**GO:0062125**	26	-0.927	1.03E-05	9.20E-04	regulation of mitochondrial gene expression
**GO:0006743**	17	-0.976	1.32E-05	1.15E-03	ubiquinone metabolic process
**GO:0000154**	33	-0.731	1.91E-05	1.57E-03	rRNA modification
**GO:0042157**	121	-0.390	2.07E-05	1.70E-03	lipoprotein metabolic process
**GO:0070129**	24	-0.942	2.39E-05	1.93E-03	regulation of mitochondrial translation
**GO:0044282**	424	-0.208	2.41E-05	1.94E-03	small molecule catabolic process
**GO:0016180**	25	-0.754	3.38E-05	2.58E-03	snRNA processing
**GO:0042273**	67	-0.496	3.63E-05	2.73E-03	ribosomal large subunit biogenesis
**GO:0006264**	12	-1.097	3.66E-05	2.74E-03	mitochondrial DNA replication
**GO:0006122**	12	-1.022	3.74E-05	2.78E-03	mitochondrial electron transport, ubiquinol to cytochrome c
**GO:0030490**	44	-0.602	3.75E-05	2.79E-03	maturation of SSU-rRNA
**GO:0042274**	63	-0.507	4.12E-05	3.01E-03	ribosomal small subunit biogenesis
**GO:0090646**	13	-1.018	4.83E-05	3.46E-03	mitochondrial tRNA processing
**GO:0000959**	43	-0.621	4.98E-05	3.54E-03	mitochondrial RNA metabolic process
**GO:0090494**	15	-0.950	5.67E-05	3.98E-03	dopamine uptake
**GO:0033013**	56	-0.529	7.54E-05	5.07E-03	tetrapyrrole metabolic process
**GO:0009301**	72	-0.462	7.64E-05	5.12E-03	snRNA transcription
**GO:0042795**	71	-0.465	7.78E-05	5.18E-03	snRNA transcription by RNA polymerase II
**GO:0090305**	282	-0.236	8.74E-05	5.74E-03	nucleic acid phosphodiester bond hydrolysis
**GO:0034982**	13	-1.059	8.77E-05	5.74E-03	mitochondrial protein processing
**GO:0000466**	22	-0.792	9.17E-05	5.96E-03	maturation of 5.8S rRNA from tricistronic rRNA transcript (SSU-rRNA, 5.8S rRNA, LSU-rRNA)
**GO:1901606**	103	-0.381	1.13E-04	7.15E-03	alpha-amino acid catabolic process
**GO:0006851**	25	-0.743	1.27E-04	7.90E-03	mitochondrial calcium ion transmembrane transport
**GO:0090501**	141	-0.325	1.35E-04	8.32E-03	RNA phosphodiester bond hydrolysis
**GO:0006488**	18	-0.928	1.42E-04	8.68E-03	dolichol-linked oligosaccharide biosynthetic process
**GO:0098781**	107	-0.366	1.50E-04	9.09E-03	ncRNA transcription
**GO:0043624**	211	-0.258	2.25E-04	1.29E-02	cellular protein complex disassembly

**GO:0006490**	19	-0.866	2.27E-04	1.30E-02	oligosaccharide-lipid intermediate biosynthetic process
**GO:0036503**	97	-0.381	2.40E-04	1.36E-02	ERAD pathway
**GO:0033014**	30	-0.686	2.52E-04	1.42E-02	tetrapyrrole biosynthetic process
**GO:0072321**	11	-0.980	2.61E-04	1.46E-02	chaperone-mediated protein transport
**GO:0000462**	34	-0.595	3.21E-04	1.74E-02	maturation of SSU-rRNA from tricistronic rRNA transcript (SSU-rRNA, 5.8S rRNA, LSU-rRNA)
**GO:0042537**	24	-0.735	3.43E-04	1.85E-02	benzene-containing compound metabolic process
**GO:1901661**	28	-0.646	3.52E-04	1.89E-02	quinone metabolic process
**GO:0007006**	125	-0.321	3.76E-04	1.99E-02	mitochondrial membrane organization
**GO:0000460**	27	-0.670	3.93E-04	2.07E-02	maturation of 5.8S rRNA
**GO:0035269**	18	-0.964	4.43E-04	2.29E-02	protein O-linked mannosylation
**GO:0006888**	203	-0.240	6.80E-04	3.32E-02	endoplasmic reticulum to Golgi vesicle-mediated transport
**GO:0090502**	70	-0.411	6.83E-04	3.33E-02	RNA phosphodiester bond hydrolysis, endonucleolytic
**GO:0016074**	13	-0.849	7.86E-04	3.75E-02	snoRNA metabolic process
**GO:0070585**	139	-0.287	8.13E-04	3.86E-02	protein localization to mitochondrion
**GO:0046034**	270	-0.205	8.46E-04	4.00E-02	ATP metabolic process
**GO:0035437**	12	-0.879	1.01E-03	4.66E-02	maintenance of protein localization in endoplasmic reticulum
**GO:0042255**	57	-0.431	1.03E-03	4.72E-02	ribosome assembly
**GO:0034976**	274	-0.200	1.07E-03	4.87E-02	response to endoplasmic reticulum stress
**GO:0015697**	12	-0.956	1.09E-03	4.96E-02	quaternary ammonium group transport
**GO:0042110**	431	0.628	6.49E-36	1.87E-31	T cell activation
**GO:0002694**	476	0.557	1.55E-31	1.49E-27	regulation of leukocyte activation
**GO:0001525**	481	0.540	4.36E-31	3.60E-27	angiogenesis
**GO:0050900**	400	0.583	1.83E-29	1.32E-25	leukocyte migration
**GO:0006909**	256	0.736	7.84E-29	5.03E-25	phagocytosis
**GO:0051249**	395	0.579	1.65E-28	9.54E-25	regulation of lymphocyte activation
**GO:0045785**	386	0.568	1.93E-27	8.58E-24	positive regulation of cell adhesion
**GO:2000147**	500	0.499	3.92E-27	1.62E-23	positive regulation of cell motility
**GO:0030335**	479	0.503	1.89E-26	7.26E-23	positive regulation of cell migration
**GO:0002521**	488	0.496	3.23E-26	1.17E-22	leukocyte differentiation
**GO:0042060**	462	0.502	5.27E-26	1.79E-22	wound healing
**GO:0002764**	353	0.569	8.01E-25	2.57E-21	immune response-regulating signaling pathway

**GO:0002768**	351	0.570	8.64E-25	2.63E-21	immune response-regulating cell surface receptor signaling pathway
**GO:0007159**	313	0.605	1.13E-24	3.27E-21	leukocyte cell-cell adhesion
**GO:0043299**	493	0.478	1.34E-24	3.69E-21	leukocyte degranulation
**GO:0050863**	296	0.610	2.35E-24	6.18E-21	regulation of T cell activation
**GO:0050867**	306	0.597	6.12E-24	1.54E-20	positive regulation of cell activation
**GO:0031589**	338	0.551	2.24E-23	5.40E-20	cell-substrate adhesion
**GO:0002250**	352	0.547	2.77E-23	6.15E-20	adaptive immune response
**GO:0002696**	297	0.597	2.73E-23	6.15E-20	positive regulation of leukocyte activation
**GO:0070661**	271	0.619	3.17E-23	6.78E-20	leukocyte proliferation
**GO:0038094**	82	1.056	5.35E-23	1.10E-19	Fc-gamma receptor signaling pathway
**GO:1903037**	280	0.611	6.09E-23	1.21E-19	regulation of leukocyte cell-cell adhesion
**GO:0022407**	375	0.517	2.15E-22	3.59E-19	regulation of cell-cell adhesion
**GO:0001819**	356	0.534	2.07E-22	3.59E-19	positive regulation of cytokine production
**GO:0002429**	323	0.563	2.24E-22	3.59E-19	immune response-activating cell surface receptor signaling pathway
**GO:0002757**	323	0.563	2.24E-22	3.59E-19	immune response-activating signal transduction
**GO:0043087**	436	0.476	3.81E-22	5.95E-19	regulation of GTPase activity
**GO:0002431**	84	1.039	4.55E-22	6.91E-19	Fc receptor mediated stimulatory signaling pathway
**GO:0030098**	332	0.540	7.20E-22	1.07E-18	lymphocyte differentiation
**GO:1901342**	310	0.558	7.81E-22	1.13E-18	regulation of vasculature development
**GO:0002433**	78	1.059	8.46E-22	1.16E-18	immune response-regulating cell surface receptor signaling pathway involved in phagocytosis
**GO:0038096**	78	1.059	8.46E-22	1.16E-18	Fc-gamma receptor signaling pathway involved in phagocytosis
**GO:0051251**	254	0.622	1.10E-21	1.48E-18	positive regulation of lymphocyte activation
**GO:0032943**	250	0.620	1.57E-21	2.06E-18	mononuclear cell proliferation
**GO:0042119**	458	0.460	1.64E-21	2.10E-18	neutrophil activation
**GO:0043547**	364	0.510	3.07E-21	3.86E-18	positive regulation of GTPase activity
**GO:0036230**	464	0.449	6.56E-21	7.90E-18	granulocyte activation
**GO:1903039**	205	0.678	6.95E-21	8.20E-18	positive regulation of leukocyte cell-cell adhesion
**GO:0046651**	248	0.610	8.00E-21	9.25E-18	lymphocyte proliferation
**GO:0043312**	447	0.456	9.09E-21	1.03E-17	neutrophil degranulation
**GO:0002283**	449	0.454	1.16E-20	1.28E-17	neutrophil activation involved in immune response

**GO:0002446**	460	0.444	2.96E-20	3.23E-17	neutrophil mediated immunity
**GO:0045765**	280	0.561	3.50E-20	3.75E-17	regulation of angiogenesis
**GO:0022604**	477	0.430	4.68E-20	4.91E-17	regulation of cell morphogenesis
**GO:0022409**	241	0.604	7.01E-20	7.23E-17	positive regulation of cell-cell adhesion
**GO:0002253**	402	0.471	7.64E-20	7.75E-17	activation of immune response
**GO:0032970**	360	0.493	1.07E-19	1.07E-16	regulation of actin filament-based process
**GO:0070663**	207	0.656	1.13E-19	1.11E-16	regulation of leukocyte proliferation
**GO:0051056**	333	0.508	1.78E-19	1.71E-16	regulation of small GTPase mediated signal transduction
**GO:0007265**	433	0.446	2.04E-19	1.93E-16	Ras protein signal transduction
**GO:0030198**	354	0.488	2.73E-19	2.51E-16	extracellular matrix organization
**GO:0043062**	355	0.486	3.23E-19	2.91E-16	extracellular structure organization
**GO:0032103**	469	0.423	4.60E-19	4.09E-16	positive regulation of response to external stimulus
**GO:0007162**	255	0.568	5.16E-19	4.52E-16	negative regulation of cell adhesion
**GO:0007015**	378	0.468	9.14E-19	7.88E-16	actin filament organization
**GO:0032956**	317	0.509	1.17E-18	9.98E-16	regulation of actin cytoskeleton organization
**GO:0032944**	197	0.648	2.19E-18	1.83E-15	regulation of mononuclear cell proliferation
**GO:0001667**	381	0.456	2.51E-18	2.07E-15	ameboidal-type cell migration
**GO:0007160**	216	0.593	3.77E-18	3.06E-15	cell-matrix adhesion
**GO:0050870**	191	0.648	4.05E-18	3.25E-15	positive regulation of T cell activation
**GO:0050878**	463	0.411	5.44E-18	4.31E-15	regulation of body fluid levels
**GO:0050670**	196	0.641	5.62E-18	4.39E-15	regulation of lymphocyte proliferation
**GO:0002683**	376	0.445	3.13E-17	2.41E-14	negative regulation of immune system process
**GO:0008360**	148	0.705	3.47E-17	2.64E-14	regulation of cell shape
**GO:0050817**	310	0.486	4.60E-17	3.41E-14	coagulation
**GO:0090066**	479	0.392	5.11E-17	3.74E-14	regulation of anatomical structure size
**GO:0002697**	351	0.460	7.35E-17	5.31E-14	regulation of immune effector process
**GO:0007599**	311	0.480	9.46E-17	6.66E-14	hemostasis
**GO:0010631**	274	0.508	1.37E-16	9.56E-14	epithelial cell migration
**GO:0090130**	283	0.498	1.67E-16	1.15E-13	tissue migration
**GO:0030099**	383	0.433	1.98E-16	1.35E-13	myeloid cell differentiation
**GO:0030217**	231	0.552	2.10E-16	1.41E-13	T cell differentiation
**GO:0007596**	306	0.477	2.48E-16	1.65E-13	blood coagulation
**GO:0090132**	277	0.501	2.51E-16	1.65E-13	epithelium migration

**GO:0071346**	146	0.690	6.70E-16	4.30E-13	cellular response to interferon-gamma
**GO:0034341**	163	0.653	7.25E-16	4.60E-13	response to interferon-gamma
**GO:0009615**	282	0.488	1.10E-15	6.89E-13	response to virus
**GO:0032535**	354	0.434	1.31E-15	8.11E-13	regulation of cellular component size
**GO:0071900**	492	0.369	1.57E-15	9.67E-13	regulation of protein serine/threonine kinase activity
**GO:0060326**	268	0.492	2.18E-15	1.33E-12	cell chemotaxis
**GO:0050673**	379	0.412	2.24E-15	1.35E-12	epithelial cell proliferation
**GO:0042098**	173	0.615	2.29E-15	1.36E-12	T cell proliferation
**GO:0010810**	204	0.556	2.89E-15	1.70E-12	regulation of cell-substrate adhesion
**GO:0038093**	179	0.605	4.72E-15	2.75E-12	Fc receptor signaling pathway
**GO:0071902**	327	0.442	4.99E-15	2.88E-12	positive regulation of protein serine/threonine kinase activity
**GO:0018212**	351	0.424	5.52E-15	3.16E-12	peptidyl-tyrosine modification
**GO:0018108**	348	0.421	1.13E-14	6.38E-12	peptidyl-tyrosine phosphorylation
**GO:0043406**	254	0.489	1.60E-14	8.90E-12	positive regulation of MAP kinase activity
**GO:0002831**	354	0.418	1.86E-14	1.01E-11	regulation of response to biotic stimulus
**GO:1902903**	323	0.436	2.04E-14	1.10E-11	regulation of supramolecular fiber organization
**GO:0030168**	149	0.639	2.07E-14	1.11E-11	platelet activation
**GO:0002221**	185	0.586	2.47E-14	1.31E-11	pattern recognition receptor signaling pathway
**GO:0051271**	295	0.445	3.69E-14	1.92E-11	negative regulation of cellular component movement
**GO:0034329**	395	0.387	3.87E-14	2.00E-11	cell junction assembly
**GO:0070665**	132	0.693	4.52E-14	2.31E-11	positive regulation of leukocyte proliferation
**GO:0031349**	338	0.420	4.57E-14	2.32E-11	positive regulation of defense response
**GO:0110053**	240	0.496	7.23E-14	3.64E-11	regulation of actin filament organization
**GO:0051607**	202	0.537	9.10E-14	4.49E-11	defense response to virus
**GO:0040013**	293	0.438	9.57E-14	4.69E-11	negative regulation of locomotion
**GO:0007409**	462	0.351	1.31E-13	6.36E-11	axonogenesis
**GO:0043405**	329	0.412	2.33E-13	1.11E-10	regulation of MAP kinase activity
**GO:0010632**	216	0.504	2.50E-13	1.18E-10	regulation of epithelial cell migration
**GO:0042129**	145	0.620	3.96E-13	1.85E-10	regulation of T cell proliferation
**GO:0042113**	224	0.496	4.36E-13	1.99E-10	B cell activation
**GO:0046631**	134	0.626	4.35E-13	1.99E-10	alpha-beta T cell activation
**GO:0032946**	127	0.678	4.31E-13	1.99E-10	positive regulation of mononuclear cell proliferation

**GO:0046777**	236	0.474	4.55E-13	2.05E-10	protein autophosphorylation
**GO:1903706**	438	0.352	5.09E-13	2.26E-10	regulation of hemopoiesis
**GO:0050853**	56	0.976	5.06E-13	2.26E-10	B cell receptor signaling pathway
**GO:1904018**	180	0.541	9.91E-13	4.37E-10	positive regulation of vasculature development
**GO:0002703**	180	0.542	1.07E-12	4.68E-10	regulation of leukocyte mediated immunity
**GO:0050671**	126	0.667	1.08E-12	4.68E-10	positive regulation of lymphocyte proliferation
**GO:0099024**	58	0.908	1.16E-12	4.98E-10	plasma membrane invagination
**GO:0050678**	324	0.399	1.21E-12	5.14E-10	regulation of epithelial cell proliferation
**GO:0008154**	192	0.521	2.10E-12	8.85E-10	actin polymerization or depolymerization
**GO:0045766**	161	0.563	2.16E-12	8.99E-10	positive regulation of angiogenesis
**GO:0048017**	176	0.532	2.84E-12	1.16E-09	inositol lipid-mediated signaling
**GO:0001952**	113	0.651	2.87E-12	1.17E-09	regulation of cell-matrix adhesion
**GO:1902105**	260	0.439	4.16E-12	1.68E-09	regulation of leukocyte differentiation
**GO:2000146**	262	0.429	5.01E-12	2.01E-09	negative regulation of cell motility
**GO:0030595**	196	0.497	5.63E-12	2.24E-09	leukocyte chemotaxis
**GO:0050866**	181	0.524	5.91E-12	2.34E-09	negative regulation of cell activation
**GO:0002460**	235	0.461	5.97E-12	2.35E-09	adaptive immune response based on somatic recombination of immune receptors built from immunoglobulin superfamily domains
**GO:0043542**	197	0.497	6.07E-12	2.37E-09	endothelial cell migration
**GO:0032147**	324	0.387	6.79E-12	2.62E-09	activation of protein kinase activity
**GO:0048015**	173	0.527	6.79E-12	2.62E-09	phosphatidylinositol-mediated signaling
**GO:1901653**	369	0.362	8.20E-12	3.14E-09	cellular response to peptide
**GO:1901343**	114	0.634	8.89E-12	3.38E-09	negative regulation of vasculature development
**GO:0031341**	80	0.748	1.07E-11	4.03E-09	regulation of cell killing
**GO:0030336**	251	0.430	1.15E-11	4.31E-09	negative regulation of cell migration
**GO:0010634**	139	0.576	1.55E-11	5.77E-09	positive regulation of epithelial cell migration
**GO:0045088**	270	0.420	1.69E-11	6.25E-09	regulation of innate immune response
**GO:0043254**	404	0.341	2.06E-11	7.54E-09	regulation of protein-containing complex assembly
**GO:0002224**	138	0.598	2.06E-11	7.54E-09	toll-like receptor signaling pathway

**GO:0032612**	43	0.953	2.26E-11	8.17E-09	interleukin-1 production
**GO:0002237**	318	0.383	2.41E-11	8.65E-09	response to molecule of bacterial origin
**GO:0002685**	185	0.499	2.58E-11	9.21E-09	regulation of leukocyte migration
**GO:0048010**	92	0.705	2.65E-11	9.39E-09	vascular endothelial growth factor receptor signaling pathway
**GO:0031295**	55	0.861	2.80E-11	9.87E-09	T cell costimulation
**GO:0002695**	160	0.536	3.10E-11	1.09E-08	negative regulation of leukocyte activation
**GO:0019932**	416	0.330	3.15E-11	1.09E-08	second-messenger-mediated signaling
**GO:0097529**	184	0.494	3.29E-11	1.14E-08	myeloid leukocyte migration
**GO:0050769**	464	0.312	3.61E-11	1.24E-08	positive regulation of neurogenesis
**GO:0031334**	234	0.440	4.03E-11	1.38E-08	positive regulation of protein-containing complex assembly
**GO:0046578**	234	0.438	4.47E-11	1.51E-08	regulation of Ras protein signal transduction
**GO:0006911**	49	0.915	4.48E-11	1.51E-08	phagocytosis, engulfment
**GO:0050920**	202	0.466	5.42E-11	1.79E-08	regulation of chemotaxis
**GO:0007229**	106	0.662	5.43E-11	1.79E-08	integrin-mediated signaling pathway
**GO:0050729**	135	0.565	7.44E-11	2.43E-08	positive regulation of inflammatory response
**GO:0046634**	89	0.681	8.70E-11	2.82E-08	regulation of alpha-beta T cell activation
**GO:0050854**	60	0.819	8.76E-11	2.83E-08	regulation of antigen receptor-mediated signaling pathway
**GO:0032496**	307	0.378	9.26E-11	2.97E-08	response to lipopolysaccharide
**GO:0001776**	84	0.734	1.03E-10	3.28E-08	leukocyte homeostasis
**GO:0002285**	161	0.519	1.12E-10	3.54E-08	lymphocyte activation involved in immune response
**GO:0002699**	188	0.485	1.13E-10	3.56E-08	positive regulation of immune effector process
**GO:2000181**	103	0.628	1.13E-10	3.56E-08	negative regulation of blood vessel morphogenesis
**GO:0010975**	487	0.297	1.24E-10	3.88E-08	regulation of neuron projection development
**GO:0031294**	56	0.831	1.33E-10	4.14E-08	lymphocyte costimulation
**GO:0008064**	163	0.515	1.49E-10	4.61E-08	regulation of actin polymerization or depolymerization
**GO:0050727**	317	0.366	1.56E-10	4.77E-08	regulation of inflammatory response
**GO:0002573**	197	0.465	1.58E-10	4.82E-08	myeloid leukocyte differentiation
**GO:0046632**	100	0.637	1.60E-10	4.86E-08	alpha-beta T cell differentiation
**GO:0007266**	198	0.460	1.69E-10	5.10E-08	Rho protein signal transduction
**GO:0051983**	98	0.667	1.81E-10	5.42E-08	regulation of chromosome segregation

**GO:0031098**	302	0.372	1.96E-10	5.83E-08	stress-activated protein kinase signaling cascade
**GO:0030832**	164	0.510	1.98E-10	5.87E-08	regulation of actin filament length
**GO:2000106**	78	0.755	2.03E-10	6.00E-08	regulation of leukocyte apoptotic process
**GO:0002449**	223	0.433	2.12E-10	6.20E-08	lymphocyte mediated immunity
**GO:0001935**	140	0.536	2.12E-10	6.20E-08	endothelial cell proliferation
**GO:0007163**	210	0.444	2.44E-10	7.09E-08	establishment or maintenance of cell polarity
**GO:0097305**	233	0.420	2.50E-10	7.23E-08	response to alcohol
**GO:0007249**	253	0.405	2.54E-10	7.31E-08	I-kappaB kinase/NF-kappaB signaling
**GO:0030900**	381	0.328	2.57E-10	7.35E-08	forebrain development
**GO:0045123**	61	0.789	2.91E-10	8.28E-08	cellular extravasation
**GO:0030041**	165	0.502	2.96E-10	8.40E-08	actin filament polymerization
**GO:0051480**	345	0.343	3.05E-10	8.60E-08	regulation of cytosolic calcium ion concentration
**GO:0050679**	185	0.466	3.08E-10	8.63E-08	positive regulation of epithelial cell proliferation
**GO:0140014**	264	0.395	3.10E-10	8.65E-08	mitotic nuclear division
**GO:0010324**	66	0.770	3.14E-10	8.72E-08	membrane invagination
**GO:0050851**	218	0.436	3.30E-10	9.14E-08	antigen receptor-mediated signaling pathway
**GO:0016525**	101	0.618	3.33E-10	9.18E-08	negative regulation of angiogenesis
**GO:0000082**	240	0.412	3.54E-10	9.69E-08	G1/S transition of mitotic cell cycle
**GO:0071496**	300	0.368	3.85E-10	1.05E-07	cellular response to external stimulus
**GO:0001906**	128	0.559	4.16E-10	1.13E-07	cell killing
**GO:0070371**	284	0.378	4.18E-10	1.13E-07	ERK1 and ERK2 cascade
**GO:0032271**	198	0.454	4.68E-10	1.26E-07	regulation of protein polymerization
**GO:0050730**	240	0.407	5.19E-10	1.39E-07	regulation of peptidyl-tyrosine phosphorylation
**GO:0048872**	245	0.404	5.39E-10	1.43E-07	homeostasis of number of cells
**GO:0032611**	35	0.961	5.47E-10	1.45E-07	interleukin-1 beta production
**GO:0007204**	308	0.357	6.30E-10	1.66E-07	positive regulation of cytosolic calcium ion concentration
**GO:0033002**	187	0.461	6.51E-10	1.71E-07	muscle cell proliferation
**GO:0030833**	146	0.526	6.75E-10	1.77E-07	regulation of actin filament polymerization
**GO:0032652**	39	0.933	6.86E-10	1.79E-07	regulation of interleukin-1 production
**GO:0000910**	160	0.500	7.05E-10	1.83E-07	cytokinesis
**GO:0071559**	236	0.405	7.32E-10	1.89E-07	response to transforming growth factor beta

**GO:0071260**	75	0.729	7.69E-10	1.98E-07	cellular response to mechanical stimulus
**GO:0007059**	301	0.362	7.85E-10	2.01E-07	chromosome segregation
**GO:0071887**	99	0.638	8.09E-10	2.06E-07	leukocyte apoptotic process
**GO:0051051**	421	0.304	8.99E-10	2.27E-07	negative regulation of transport
**GO:0042102**	90	0.672	9.13E-10	2.30E-07	positive regulation of T cell proliferation
**GO:0001910**	61	0.763	1.09E-09	2.71E-07	regulation of leukocyte mediated cytotoxicity
**GO:0045619**	160	0.491	1.10E-09	2.74E-07	regulation of lymphocyte differentiation
**GO:0010594**	155	0.494	1.11E-09	2.74E-07	regulation of endothelial cell migration
**GO:0070302**	226	0.412	1.18E-09	2.90E-07	regulation of stress-activated protein kinase signaling cascade
**GO:0019882**	195	0.445	1.23E-09	3.02E-07	antigen processing and presentation
**GO:0043122**	222	0.413	1.27E-09	3.10E-07	regulation of I-kappaB kinase/NF-kappaB signaling
**GO:0060348**	215	0.418	1.31E-09	3.17E-07	bone development
**GO:0071375**	312	0.348	1.31E-09	3.18E-07	cellular response to peptide hormone stimulus
**GO:0051235**	298	0.356	1.52E-09	3.65E-07	maintenance of location
**GO:0031343**	54	0.817	1.78E-09	4.24E-07	positive regulation of cell killing
**GO:0072678**	60	0.803	1.80E-09	4.29E-07	T cell migration
**GO:0007044**	98	0.604	1.88E-09	4.44E-07	cell-substrate junction assembly
**GO:0150115**	98	0.604	1.88E-09	4.44E-07	cell-substrate junction organization
**GO:0010769**	299	0.349	2.47E-09	5.81E-07	regulation of cell morphogenesis involved in differentiation
**GO:0032273**	125	0.541	2.51E-09	5.86E-07	positive regulation of protein polymerization
**GO:0019058**	299	0.349	2.78E-09	6.47E-07	viral life cycle
**GO:0045637**	221	0.407	2.95E-09	6.84E-07	regulation of myeloid cell differentiation
**GO:0022408**	161	0.472	3.31E-09	7.66E-07	negative regulation of cell-cell adhesion
**GO:0044843**	254	0.377	3.40E-09	7.82E-07	cell cycle G1/S phase transition
**GO:0150076**	63	0.745	3.41E-09	7.82E-07	neuroinflammatory response
**GO:0009612**	209	0.417	3.45E-09	7.89E-07	response to mechanical stimulus
**GO:0060560**	231	0.393	3.48E-09	7.93E-07	developmental growth involved in morphogenesis
**GO:0051250**	132	0.524	3.89E-09	8.82E-07	negative regulation of lymphocyte activation
**GO:0043434**	426	0.290	3.94E-09	8.89E-07	response to peptide hormone
**GO:0032872**	224	0.399	4.03E-09	9.05E-07	regulation of stress-activated MAPK cascade
**GO:0006874**	440	0.285	4.19E-09	9.37E-07	cellular calcium ion homeostasis

**GO:0001936**	124	0.526	4.20E-09	9.37E-07	regulation of endothelial cell proliferation
**GO:0002706**	128	0.528	4.23E-09	9.40E-07	regulation of lymphocyte mediated immunity
**GO:0008361**	177	0.444	4.41E-09	9.76E-07	regulation of cell size
**GO:0050764**	91	0.639	4.54E-09	1.00E-06	regulation of phagocytosis
**GO:0070372**	268	0.364	4.74E-09	1.04E-06	regulation of ERK1 and ERK2 cascade
**GO:0006898**	243	0.378	4.92E-09	1.08E-06	receptor-mediated endocytosis
**GO:0030888**	61	0.790	5.09E-09	1.11E-06	regulation of B cell proliferation
**GO:0002819**	144	0.498	5.13E-09	1.12E-06	regulation of adaptive immune response
**GO:0060485**	263	0.362	6.13E-09	1.33E-06	mesenchyme development
**GO:0043123**	176	0.444	6.73E-09	1.45E-06	positive regulation of I-kappaB kinase/NF-kappaB signaling
**GO:0014065**	142	0.490	6.82E-09	1.46E-06	phosphatidylinositol 3-kinase signaling
**GO:0072676**	94	0.616	6.96E-09	1.49E-06	lymphocyte migration
**GO:0000819**	176	0.446	7.03E-09	1.49E-06	sister chromatid segregation
**GO:0061572**	150	0.478	7.12E-09	1.50E-06	actin filament bundle organization
**GO:0042100**	82	0.653	7.11E-09	1.50E-06	B cell proliferation
**GO:0055074**	452	0.277	7.20E-09	1.51E-06	calcium ion homeostasis
**GO:0071560**	230	0.385	7.31E-09	1.53E-06	cellular response to transforming growth factor beta stimulus
**GO:0097530**	125	0.520	8.26E-09	1.72E-06	granulocyte migration
**GO:0051403**	274	0.353	8.44E-09	1.75E-06	stress-activated MAPK cascade
**GO:0048608**	425	0.283	8.76E-09	1.81E-06	reproductive structure development
**GO:0003012**	432	0.281	8.88E-09	1.83E-06	muscle system process
**GO:0060333**	65	0.720	9.39E-09	1.92E-06	interferon-gamma-mediated signaling pathway
**GO:0050868**	101	0.577	1.02E-08	2.09E-06	negative regulation of T cell activation
**GO:0031532**	99	0.578	1.03E-08	2.10E-06	actin cytoskeleton reorganization
**GO:0045580**	131	0.506	1.11E-08	2.26E-06	regulation of T cell differentiation
**GO:0048771**	161	0.455	1.12E-08	2.26E-06	tissue remodeling
**GO:0045931**	149	0.477	1.14E-08	2.29E-06	positive regulation of mitotic cell cycle
**GO:0002228**	51	0.761	1.16E-08	2.34E-06	natural killer cell mediated immunity
**GO:0031346**	373	0.298	1.27E-08	2.54E-06	positive regulation of cell projection organization
**GO:0043491**	239	0.374	1.37E-08	2.72E-06	protein kinase B signaling
**GO:0042116**	88	0.602	1.39E-08	2.75E-06	macrophage activation
**GO:0048041**	82	0.619	1.45E-08	2.88E-06	focal adhesion assembly

**GO:1901888**	191	0.413	1.48E-08	2.92E-06	regulation of cell junction assembly
**GO:0034765**	462	0.267	1.53E-08	3.00E-06	regulation of ion transmembrane transport
**GO:0038083**	37	0.844	1.55E-08	3.03E-06	peptidyl-tyrosine autophosphorylation
**GO:0051090**	405	0.285	1.68E-08	3.28E-06	regulation of DNA-binding transcription factor activity
**GO:0048638**	327	0.316	1.70E-08	3.31E-06	regulation of developmental growth
**GO:0001818**	209	0.396	1.71E-08	3.31E-06	negative regulation of cytokine production
**GO:0032651**	31	0.938	1.90E-08	3.66E-06	regulation of interleukin-1 beta production
**GO:0002833**	221	0.385	1.96E-08	3.74E-06	positive regulation of response to biotic stimulus
**GO:0002286**	89	0.599	2.05E-08	3.91E-06	T cell activation involved in immune response
**GO:0050864**	113	0.549	2.12E-08	4.03E-06	regulation of B cell activation
**GO:1902905**	193	0.408	2.14E-08	4.06E-06	positive regulation of supramolecular fiber organization
**GO:0042267**	48	0.773	2.17E-08	4.09E-06	natural killer cell mediated cytotoxicity
**GO:0043534**	112	0.537	2.24E-08	4.22E-06	blood vessel endothelial cell migration
**GO:1903829**	321	0.317	2.26E-08	4.24E-06	positive regulation of cellular protein localization
**GO:0061458**	428	0.274	2.29E-08	4.28E-06	reproductive system development
**GO:0050808**	393	0.285	2.45E-08	4.57E-06	synapse organization
**GO:0046822**	107	0.547	2.48E-08	4.62E-06	regulation of nucleocytoplasmic transport
**GO:0014066**	118	0.517	2.66E-08	4.93E-06	regulation of phosphatidylinositol 3-kinase signaling
**GO:0034109**	80	0.616	3.04E-08	5.62E-06	homotypic cell-cell adhesion
**GO:0048660**	132	0.489	3.21E-08	5.91E-06	regulation of smooth muscle cell proliferation
**GO:0050731**	177	0.419	3.34E-08	6.12E-06	positive regulation of peptidyl-tyrosine phosphorylation
**GO:0010959**	369	0.291	3.40E-08	6.22E-06	regulation of metal ion transport
**GO:0072503**	467	0.259	3.51E-08	6.40E-06	cellular divalent inorganic cation homeostasis
**GO:0051017**	148	0.457	3.55E-08	6.44E-06	actin filament bundle assembly
**GO:0001909**	88	0.599	3.55E-08	6.44E-06	leukocyte mediated cytotoxicity
**GO:0003158**	119	0.508	3.63E-08	6.56E-06	endothelium development
**GO:0071216**	211	0.388	3.64E-08	6.56E-06	cellular response to biotic stimulus

**GO:0010721**	326	0.308	3.73E-08	6.69E-06	negative regulation of cell development
**GO:0048588**	219	0.375	3.84E-08	6.87E-06	developmental cell growth
**GO:0048659**	133	0.483	4.07E-08	7.26E-06	smooth muscle cell proliferation
**GO:0051258**	260	0.345	4.34E-08	7.72E-06	protein polymerization
**GO:0051893**	61	0.688	4.51E-08	7.95E-06	regulation of focal adhesion assembly
**GO:0090109**	61	0.688	4.51E-08	7.95E-06	regulation of cell-substrate junction assembly
**GO:0150116**	61	0.688	4.51E-08	7.95E-06	regulation of cell-substrate junction organization
**GO:0002040**	112	0.518	4.64E-08	8.16E-06	sprouting angiogenesis
**GO:0000070**	144	0.464	4.67E-08	8.17E-06	mitotic sister chromatid segregation
**GO:0060537**	384	0.282	4.69E-08	8.19E-06	muscle tissue development
**GO:0070374**	193	0.398	4.75E-08	8.26E-06	positive regulation of ERK1 and ERK2 cascade
**GO:0051283**	122	0.503	4.77E-08	8.27E-06	negative regulation of sequestering of calcium ion
**GO:1903708**	179	0.414	5.39E-08	9.29E-06	positive regulation of hemopoiesis
**GO:1990266**	104	0.536	5.38E-08	9.29E-06	neutrophil migration
**GO:0072507**	486	0.251	5.47E-08	9.41E-06	divalent inorganic cation homeostasis
**GO:0043535**	88	0.585	5.62E-08	9.63E-06	regulation of blood vessel endothelial cell migration
**GO:0048762**	211	0.377	5.81E-08	9.94E-06	mesenchymal cell differentiation
**GO:0007179**	181	0.406	5.95E-08	1.01E-05	transforming growth factor beta receptor signaling pathway
**GO:0044409**	126	0.485	6.05E-08	1.03E-05	entry into host
**GO:0051924**	238	0.356	6.23E-08	1.06E-05	regulation of calcium ion transport
**GO:0030593**	89	0.571	6.35E-08	1.07E-05	neutrophil chemotaxis
**GO:0030183**	118	0.503	6.40E-08	1.08E-05	B cell differentiation
**GO:0071674**	77	0.608	6.58E-08	1.10E-05	mononuclear cell migration
**GO:0060759**	160	0.434	6.94E-08	1.16E-05	regulation of response to cytokine stimulus
**GO:0014706**	368	0.284	7.04E-08	1.18E-05	striated muscle tissue development
**GO:0002504**	83	0.620	7.21E-08	1.20E-05	antigen processing and presentation of peptide or polysaccharide antigen via MHC class II
**GO:0071222**	181	0.409	7.28E-08	1.21E-05	cellular response to lipopolysaccharide
**GO:0045787**	359	0.288	7.35E-08	1.22E-05	positive regulation of cell cycle
**GO:0002705**	119	0.504	7.38E-08	1.22E-05	positive regulation of leukocyte mediated immunity
**GO:0045576**	57	0.722	7.55E-08	1.24E-05	mast cell activation

**GO:0002822**	130	0.482	7.64E-08	1.25E-05	regulation of adaptive immune response based on somatic recombination of immune receptors built from immunoglobulin superfamily domains
**GO:0061900**	51	0.751	7.92E-08	1.29E-05	glial cell activation
**GO:0014812**	79	0.593	7.97E-08	1.30E-05	muscle cell migration
**GO:0001774**	43	0.811	8.18E-08	1.32E-05	microglial cell activation
**GO:0002269**	43	0.811	8.18E-08	1.32E-05	leukocyte activation involved in inflammatory response
**GO:0051897**	163	0.423	8.54E-08	1.38E-05	positive regulation of protein kinase B signaling
**GO:0006816**	415	0.267	8.60E-08	1.38E-05	calcium ion transport
**GO:0014068**	86	0.580	9.44E-08	1.51E-05	positive regulation of phosphatidylinositol 3-kinase signaling
**GO:0001503**	380	0.277	9.51E-08	1.51E-05	ossification
**GO:0030101**	67	0.644	9.99E-08	1.59E-05	natural killer cell activation
**GO:0071621**	107	0.515	1.00E-07	1.59E-05	granulocyte chemotaxis
**GO:0051896**	214	0.370	1.02E-07	1.61E-05	regulation of protein kinase B signaling
**GO:0051282**	123	0.488	1.05E-07	1.65E-05	regulation of sequestering of calcium ion
**GO:0048511**	297	0.311	1.09E-07	1.71E-05	rhythmic process
**GO:0046847**	57	0.687	1.09E-07	1.71E-05	filopodium assembly
**GO:0001655**	334	0.294	1.10E-07	1.72E-05	urogenital system development
**GO:0002495**	82	0.615	1.12E-07	1.75E-05	antigen processing and presentation of peptide antigen via MHC class II
**GO:0071219**	187	0.396	1.14E-07	1.77E-05	cellular response to molecule of bacterial origin
**GO:0098813**	246	0.343	1.16E-07	1.79E-05	nuclear chromosome segregation
**GO:0070838**	460	0.251	1.18E-07	1.82E-05	divalent metal ion transport
**GO:0032609**	75	0.623	1.24E-07	1.92E-05	interferon-gamma production
**GO:0061640**	95	0.564	1.26E-07	1.94E-05	cytoskeleton-dependent cytokinesis
**GO:0031032**	191	0.387	1.31E-07	2.01E-05	actomyosin structure organization
**GO:0072511**	464	0.249	1.36E-07	2.08E-05	divalent inorganic cation transport
**GO:0051209**	120	0.489	1.38E-07	2.10E-05	release of sequestered calcium ion into cytosol
**GO:0048013**	85	0.587	1.40E-07	2.12E-05	ephrin receptor signaling pathway
**GO:0050772**	84	0.568	1.41E-07	2.13E-05	positive regulation of axonogenesis
**GO:0050770**	182	0.394	1.42E-07	2.14E-05	regulation of axonogenesis

**GO:1903034**	155	0.430	1.48E-07	2.23E-05	regulation of response to wounding
**GO:1902107**	141	0.450	1.52E-07	2.29E-05	positive regulation of leukocyte differentiation
**GO:0001959**	151	0.434	1.54E-07	2.32E-05	regulation of cytokine-mediated signaling pathway
**GO:0071214**	317	0.299	1.56E-07	2.33E-05	cellular response to abiotic stimulus
**GO:0104004**	317	0.299	1.56E-07	2.33E-05	cellular response to environmental stimulus
**GO:0051208**	126	0.474	1.62E-07	2.41E-05	sequestering of calcium ion
**GO:0031099**	187	0.389	1.67E-07	2.48E-05	regeneration
**GO:1903038**	112	0.503	1.67E-07	2.48E-05	negative regulation of leukocyte cell-cell adhesion
**GO:0002715**	35	0.821	1.75E-07	2.58E-05	regulation of natural killer cell mediated immunity
**GO:0021700**	275	0.317	1.97E-07	2.91E-05	developmental maturation
**GO:0010811**	119	0.479	1.98E-07	2.92E-05	positive regulation of cell-substrate adhesion
**GO:0048732**	442	0.250	2.05E-07	3.01E-05	gland development
**GO:0033627**	67	0.639	2.08E-07	3.05E-05	cell adhesion mediated by integrin
**GO:0010718**	46	0.780	2.10E-07	3.07E-05	positive regulation of epithelial to mesenchymal transition
**GO:0032732**	19	1.101	2.11E-07	3.07E-05	positive regulation of interleukin-1 production
**GO:0090068**	271	0.320	2.12E-07	3.07E-05	positive regulation of cell cycle process
**GO:0043112**	186	0.383	2.17E-07	3.14E-05	receptor metabolic process
**GO:0061028**	41	0.805	2.17E-07	3.14E-05	establishment of endothelial barrier
**GO:0007088**	160	0.415	2.18E-07	3.14E-05	regulation of mitotic nuclear division
**GO:0018209**	294	0.306	2.24E-07	3.23E-05	peptidyl-serine modification
**GO:0002064**	207	0.361	2.29E-07	3.29E-05	epithelial cell development
**GO:0048871**	466	0.243	2.33E-07	3.33E-05	multicellular organismal homeostasis
**GO:0007221**	18	1.162	2.39E-07	3.41E-05	positive regulation of transcription of Notch receptor target
**GO:0000280**	397	0.263	2.42E-07	3.44E-05	nuclear division
**GO:1903306**	24	1.026	2.57E-07	3.64E-05	negative regulation of regulated secretory pathway
**GO:0042269**	33	0.836	2.61E-07	3.69E-05	regulation of natural killer cell mediated cytotoxicity
**GO:0042063**	286	0.308	2.66E-07	3.75E-05	gliogenesis
**GO:0046635**	61	0.653	2.72E-07	3.82E-05	positive regulation of alpha-beta T cell activation
**GO:1990138**	164	0.404	2.76E-07	3.87E-05	neuron projection extension

**GO:0050766**	63	0.677	2.78E-07	3.90E-05	positive regulation of phagocytosis
**GO:0007411**	271	0.315	2.83E-07	3.96E-05	axon guidance
**GO:0035710**	89	0.549	2.87E-07	3.99E-05	CD4-positive, alpha-beta T cell activation
**GO:0021537**	249	0.327	3.01E-07	4.19E-05	telencephalon development
**GO:0043367**	73	0.602	3.10E-07	4.30E-05	CD4-positive, alpha-beta T cell differentiation
**GO:0051495**	215	0.353	3.23E-07	4.47E-05	positive regulation of cytoskeleton organization
**GO:1903555**	106	0.512	3.24E-07	4.47E-05	regulation of tumor necrosis factor superfamily cytokine production
**GO:0038127**	135	0.443	3.35E-07	4.61E-05	ERBB signaling pathway
**GO:0032649**	69	0.630	3.36E-07	4.61E-05	regulation of interferon-gamma production
**GO:0097485**	272	0.313	3.41E-07	4.67E-05	neuron projection guidance
**GO:0048675**	117	0.473	3.47E-07	4.74E-05	axon extension
**GO:0046637**	62	0.646	3.50E-07	4.77E-05	regulation of alpha-beta T cell differentiation
**GO:0097191**	215	0.351	3.66E-07	4.98E-05	extrinsic apoptotic signaling pathway
**GO:0051656**	398	0.259	3.72E-07	5.03E-05	establishment of organelle localization
**GO:0019886**	79	0.600	3.71E-07	5.03E-05	antigen processing and presentation of exogenous peptide antigen via MHC class II
**GO:0090257**	226	0.341	3.76E-07	5.07E-05	regulation of muscle system process
**GO:0051047**	321	0.287	3.85E-07	5.18E-05	positive regulation of secretion
**GO:0071706**	110	0.500	3.92E-07	5.27E-05	tumor necrosis factor superfamily cytokine production
**GO:0050855**	26	0.944	3.93E-07	5.27E-05	regulation of B cell receptor signaling pathway
**GO:0046328**	180	0.383	4.24E-07	5.68E-05	regulation of JNK cascade
**GO:0035023**	137	0.435	4.35E-07	5.79E-05	regulation of Rho protein signal transduction
**GO:0101023**	18	1.045	4.36E-07	5.79E-05	vascular endothelial cell proliferation
**GO:1905562**	18	1.045	4.36E-07	5.79E-05	regulation of vascular endothelial cell proliferation
**GO:0060840**	88	0.532	4.42E-07	5.86E-05	artery development
**GO:0050890**	291	0.299	4.52E-07	5.97E-05	cognition
**GO:0002708**	88	0.552	4.65E-07	6.14E-05	positive regulation of lymphocyte mediated immunity
**GO:1901214**	308	0.290	4.79E-07	6.30E-05	regulation of neuron death
**GO:0048639**	169	0.389	4.84E-07	6.36E-05	positive regulation of developmental growth
**GO:0019722**	204	0.354	5.17E-07	6.78E-05	calcium-mediated signaling

**GO:0045216**	182	0.376	5.20E-07	6.80E-05	cell-cell junction organization
**GO:0001912**	42	0.764	5.54E-07	7.23E-05	positive regulation of leukocyte mediated cytotoxicity
**GO:0042542**	132	0.446	5.67E-07	7.38E-05	response to hydrogen peroxide
**GO:0007051**	162	0.401	5.72E-07	7.43E-05	spindle organization
**GO:0007254**	210	0.349	5.95E-07	7.71E-05	JNK cascade
**GO:0045621**	89	0.533	6.26E-07	8.10E-05	positive regulation of lymphocyte differentiation
**GO:0071349**	48	0.743	6.31E-07	8.14E-05	cellular response to interleukin-12
**GO:0042742**	192	0.362	6.84E-07	8.79E-05	defense response to bacterium
**GO:0097553**	136	0.430	6.95E-07	8.90E-05	calcium ion transmembrane import into cytosol
**GO:0007173**	113	0.472	7.50E-07	9.59E-05	epidermal growth factor receptor signaling pathway
**GO:0070671**	49	0.726	7.88E-07	1.01E-04	response to interleukin-12
**GO:0045666**	358	0.264	8.05E-07	1.03E-04	positive regulation of neuron differentiation
**GO:0051092**	147	0.413	8.29E-07	1.05E-04	positive regulation of NF-kappaB transcription factor activity
**GO:0045428**	57	0.654	8.94E-07	1.13E-04	regulation of nitric oxide biosynthetic process
**GO:0045446**	101	0.489	9.48E-07	1.20E-04	endothelial cell differentiation
**GO:0050921**	132	0.430	9.65E-07	1.21E-04	positive regulation of chemotaxis
**GO:0016049**	465	0.230	9.94E-07	1.25E-04	cell growth
**GO:0007611**	255	0.310	9.92E-07	1.25E-04	learning or memory
**GO:0000302**	218	0.336	1.02E-06	1.28E-04	response to reactive oxygen species
**GO:0060402**	155	0.397	1.03E-06	1.28E-04	calcium ion transport into cytosol
**GO:0032635**	88	0.530	1.03E-06	1.28E-04	interleukin-6 production
**GO:0070486**	12	1.214	1.09E-06	1.35E-04	leukocyte aggregation
**GO:0048002**	162	0.389	1.13E-06	1.40E-04	antigen processing and presentation of peptide antigen
**GO:0070482**	377	0.254	1.14E-06	1.41E-04	response to oxygen levels
**GO:0062197**	330	0.272	1.15E-06	1.42E-04	cellular response to chemical stress
**GO:0032102**	333	0.270	1.17E-06	1.44E-04	negative regulation of response to external stimulus
**GO:0045058**	48	0.706	1.17E-06	1.44E-04	T cell selection
**GO:0002687**	124	0.441	1.20E-06	1.47E-04	positive regulation of leukocyte migration
**GO:0007187**	232	0.321	1.20E-06	1.47E-04	G protein-coupled receptor signaling pathway, coupled to cyclic nucleotide second messenger
**GO:0070664**	71	0.583	1.23E-06	1.50E-04	negative regulation of leukocyte proliferation
**GO:0030316**	94	0.509	1.27E-06	1.55E-04	osteoclast differentiation

**GO:0051098**	356	0.260	1.28E-06	1.55E-04	regulation of binding
**GO:0035265**	180	0.364	1.28E-06	1.55E-04	organ growth
**GO:0002260**	60	0.653	1.29E-06	1.56E-04	lymphocyte homeostasis
**GO:0046718**	113	0.456	1.30E-06	1.57E-04	viral entry into host cell
**GO:0018105**	274	0.296	1.33E-06	1.60E-04	peptidyl-serine phosphorylation
**GO:0010812**	60	0.621	1.34E-06	1.61E-04	negative regulation of cell-substrate adhesion
**GO:0051651**	208	0.339	1.38E-06	1.66E-04	maintenance of location in cell
**GO:0051091**	255	0.306	1.41E-06	1.69E-04	positive regulation of DNA-binding transcription factor activity
**GO:0014909**	66	0.583	1.41E-06	1.69E-04	smooth muscle cell migration
**GO:0051961**	306	0.278	1.48E-06	1.76E-04	negative regulation of nervous system development
**GO:0030865**	47	0.725	1.50E-06	1.78E-04	cortical cytoskeleton organization
**GO:0006606**	135	0.419	1.52E-06	1.80E-04	protein import into nucleus
**GO:0002292**	66	0.599	1.57E-06	1.85E-04	T cell differentiation involved in immune response
**GO:0030838**	92	0.506	1.60E-06	1.88E-04	positive regulation of actin filament polymerization
**GO:0001953**	37	0.763	1.60E-06	1.88E-04	negative regulation of cell-matrix adhesion
**GO:0045089**	190	0.353	1.64E-06	1.93E-04	positive regulation of innate immune response
**GO:2000273**	34	0.808	1.74E-06	2.03E-04	positive regulation of signaling receptor activity
**GO:0051057**	73	0.563	1.78E-06	2.07E-04	positive regulation of small GTPase mediated signal transduction
**GO:0010770**	152	0.389	1.82E-06	2.11E-04	positive regulation of cell morphogenesis involved in differentiation
**GO:0032675**	83	0.535	1.82E-06	2.11E-04	regulation of interleukin-6 production
**GO:0000281**	70	0.590	1.92E-06	2.23E-04	mitotic cytokinesis
**GO:0002577**	16	1.058	1.95E-06	2.26E-04	regulation of antigen processing and presentation
**GO:0033047**	65	0.594	1.97E-06	2.27E-04	regulation of mitotic sister chromatid segregation
**GO:0010595**	97	0.485	2.00E-06	2.30E-04	positive regulation of endothelial cell migration
**GO:0048545**	376	0.248	2.03E-06	2.33E-04	response to steroid hormone
**GO:0045773**	41	0.719	2.05E-06	2.35E-04	positive regulation of axon extension
**GO:1990868**	85	0.524	2.07E-06	2.36E-04	response to chemokine
**GO:1990869**	85	0.524	2.07E-06	2.36E-04	cellular response to chemokine
**GO:0001771**	14	1.100	2.15E-06	2.45E-04	immunological synapse formation

**GO:0038063**	10	1.175	2.16E-06	2.45E-04	collagen-activated tyrosine kinase receptor signaling pathway
**GO:0032680**	103	0.479	2.18E-06	2.47E-04	regulation of tumor necrosis factor production
**GO:1901654**	194	0.342	2.19E-06	2.48E-04	response to ketone
**GO:0007188**	205	0.332	2.21E-06	2.49E-04	adenylate cyclase-modulating G protein-coupled receptor signaling pathway
**GO:0019935**	199	0.337	2.24E-06	2.51E-04	cyclic-nucleotide-mediated signaling
**GO:0046824**	64	0.587	2.29E-06	2.57E-04	positive regulation of nucleocytoplasmic transport
**GO:0042493**	394	0.241	2.32E-06	2.60E-04	response to drug
**GO:0043536**	54	0.641	2.33E-06	2.60E-04	positive regulation of blood vessel endothelial cell migration
**GO:0001894**	216	0.324	2.34E-06	2.61E-04	tissue homeostasis
**GO:0030866**	42	0.757	2.36E-06	2.63E-04	cortical actin cytoskeleton organization
**GO:0043277**	46	0.682	2.37E-06	2.63E-04	apoptotic cell clearance
**GO:0030219**	74	0.562	2.38E-06	2.64E-04	megakaryocyte differentiation
**GO:0045582**	78	0.541	2.43E-06	2.68E-04	positive regulation of T cell differentiation
**GO:0036005**	12	1.140	2.43E-06	2.68E-04	response to macrophage colony-stimulating factor
**GO:0036006**	12	1.140	2.43E-06	2.68E-04	cellular response to macrophage colony-stimulating factor stimulus
**GO:0030010**	134	0.411	2.49E-06	2.73E-04	establishment of cell polarity
**GO:0016202**	132	0.411	2.51E-06	2.75E-04	regulation of striated muscle tissue development
**GO:0032640**	107	0.468	2.53E-06	2.77E-04	tumor necrosis factor production
**GO:0032835**	61	0.598	2.58E-06	2.81E-04	glomerulus development
**GO:0034103**	75	0.530	2.64E-06	2.88E-04	regulation of tissue remodeling
**GO:0070997**	343	0.256	2.67E-06	2.91E-04	neuron death
**GO:0002821**	94	0.497	2.70E-06	2.94E-04	positive regulation of adaptive immune response
**GO:0002468**	12	1.202	2.83E-06	3.06E-04	dendritic cell antigen processing and presentation
**GO:0014910**	60	0.590	2.84E-06	3.07E-04	regulation of smooth muscle cell migration
**GO:1902532**	477	0.217	2.96E-06	3.18E-04	negative regulation of intracellular signal transduction
**GO:0051261**	105	0.469	2.99E-06	3.21E-04	protein depolymerization
**GO:1901222**	101	0.471	2.99E-06	3.21E-04	regulation of NIK/NF-kappaB signaling
**GO:0002548**	52	0.634	3.10E-06	3.32E-04	monocyte chemotaxis
**GO:0014896**	87	0.506	3.14E-06	3.35E-04	muscle hypertrophy
**GO:0000187**	147	0.386	3.17E-06	3.38E-04	activation of MAPK activity

**GO:0051701**	190	0.341	3.23E-06	3.43E-04	interaction with host
**GO:0045920**	32	0.869	3.23E-06	3.43E-04	negative regulation of exocytosis
**GO:0048661**	85	0.515	3.28E-06	3.47E-04	positive regulation of smooth muscle cell proliferation
**GO:1901861**	135	0.401	3.29E-06	3.47E-04	regulation of muscle tissue development
**GO:0001666**	343	0.254	3.38E-06	3.56E-04	response to hypoxia
**GO:0061041**	126	0.422	3.38E-06	3.56E-04	regulation of wound healing
**GO:0034446**	98	0.474	3.50E-06	3.67E-04	substrate adhesion-dependent cell spreading
**GO:0050858**	25	0.883	3.58E-06	3.75E-04	negative regulation of antigen receptor-mediated signaling pathway
**GO:0031102**	57	0.608	3.62E-06	3.78E-04	neuron projection regeneration
**GO:0034121**	63	0.626	3.64E-06	3.79E-04	regulation of toll-like receptor signaling pathway
**GO:0001937**	37	0.739	3.68E-06	3.83E-04	negative regulation of endothelial cell proliferation
**GO:0099177**	433	0.225	3.72E-06	3.87E-04	regulation of trans-synaptic signaling
**GO:0007052**	103	0.463	3.74E-06	3.88E-04	mitotic spindle organization
**GO:0035722**	46	0.701	3.80E-06	3.94E-04	interleukin-12-mediated signaling pathway
**GO:0046633**	31	0.798	3.84E-06	3.97E-04	alpha-beta T cell proliferation
**GO:0032388**	212	0.322	3.90E-06	4.00E-04	positive regulation of intracellular transport
**GO:0010976**	273	0.282	3.92E-06	4.02E-04	positive regulation of neuron projection development
**GO:0051302**	157	0.371	4.05E-06	4.14E-04	regulation of cell division
**GO:2000514**	58	0.606	4.15E-06	4.23E-04	regulation of CD4-positive, alpha-beta T cell activation
**GO:0019884**	155	0.376	4.20E-06	4.28E-04	antigen processing and presentation of exogenous antigen
**GO:1903557**	52	0.682	4.20E-06	4.28E-04	positive regulation of tumor necrosis factor superfamily cytokine production
**GO:0061515**	70	0.578	4.28E-06	4.35E-04	myeloid cell development
**GO:0006968**	44	0.729	4.32E-06	4.38E-04	cellular defense response
**GO:0007517**	383	0.237	4.43E-06	4.49E-04	muscle organ development
**GO:0002440**	177	0.352	4.46E-06	4.50E-04	production of molecular mediator of immune response
**GO:0048634**	135	0.396	4.48E-06	4.51E-04	regulation of muscle organ development
**GO:0110020**	89	0.491	4.62E-06	4.66E-04	regulation of actomyosin structure organization
**GO:0036293**	353	0.247	4.74E-06	4.77E-04	response to decreased oxygen levels

**GO:0150077**	34	0.765	4.93E-06	4.95E-04	regulation of neuroinflammatory response
**GO:0045471**	130	0.401	4.94E-06	4.95E-04	response to ethanol
**GO:0043303**	45	0.689	5.04E-06	5.04E-04	mast cell degranulation
**GO:0032729**	41	0.716	5.20E-06	5.18E-04	positive regulation of interferon-gamma production
**GO:2000114**	21	0.990	5.20E-06	5.18E-04	regulation of establishment of cell polarity
**GO:0050804**	432	0.222	5.25E-06	5.22E-04	modulation of chemical synaptic transmission
**GO:0051783**	185	0.338	5.27E-06	5.22E-04	regulation of nuclear division
**GO:1901992**	77	0.536	5.26E-06	5.22E-04	positive regulation of mitotic cell cycle phase transition
**GO:0032945**	66	0.566	5.40E-06	5.33E-04	negative regulation of mononuclear cell proliferation
**GO:0050672**	66	0.566	5.40E-06	5.33E-04	negative regulation of lymphocyte proliferation
**GO:0035904**	53	0.602	5.50E-06	5.43E-04	aorta development
**GO:0050768**	285	0.272	5.57E-06	5.46E-04	negative regulation of neurogenesis
**GO:0019079**	106	0.446	5.56E-06	5.46E-04	viral genome replication
**GO:0046579**	65	0.568	5.56E-06	5.46E-04	positive regulation of Ras protein signal transduction
**GO:0008037**	140	0.386	5.59E-06	5.48E-04	cell recognition
**GO:0071604**	37	0.725	5.61E-06	5.49E-04	transforming growth factor beta production
**GO:0030038**	94	0.471	5.69E-06	5.55E-04	contractile actin filament bundle assembly
**GO:0043149**	94	0.471	5.69E-06	5.55E-04	stress fiber assembly
**GO:0014897**	85	0.498	5.80E-06	5.64E-04	striated muscle hypertrophy
**GO:1902850**	128	0.407	5.90E-06	5.73E-04	microtubule cytoskeleton organization involved in mitosis
**GO:0043588**	316	0.257	5.93E-06	5.74E-04	skin development
**GO:0050856**	37	0.731	5.93E-06	5.74E-04	regulation of T cell receptor signaling pathway
**GO:0002062**	118	0.417	6.00E-06	5.80E-04	chondrocyte differentiation
**GO:0032608**	43	0.763	6.01E-06	5.80E-04	interferon-beta production
**GO:0007568**	302	0.264	6.10E-06	5.88E-04	aging
**GO:0048285**	438	0.219	6.53E-06	6.26E-04	organelle fission
**GO:0061387**	107	0.439	6.58E-06	6.28E-04	regulation of extent of cell growth
**GO:0003300**	82	0.505	6.57E-06	6.28E-04	cardiac muscle hypertrophy
**GO:0002604**	11	1.226	6.78E-06	6.46E-04	regulation of dendritic cell antigen processing and presentation
**GO:2000401**	58	0.621	6.84E-06	6.51E-04	regulation of lymphocyte migration
**GO:0033045**	77	0.520	6.89E-06	6.55E-04	regulation of sister chromatid segregation

**GO:1990090**	52	0.627	6.92E-06	6.57E-04	cellular response to nerve growth factor stimulus
**GO:0035924**	54	0.630	7.21E-06	6.82E-04	cellular response to vascular endothelial growth factor stimulus
**GO:0030516**	92	0.471	7.30E-06	6.89E-04	regulation of axon extension
**GO:0098751**	34	0.908	7.75E-06	7.30E-04	bone cell development
**GO:1902882**	82	0.503	7.93E-06	7.41E-04	regulation of response to oxidative stress
**GO:0043368**	35	0.782	7.92E-06	7.41E-04	positive T cell selection
**GO:0033032**	28	0.829	7.91E-06	7.41E-04	regulation of myeloid cell apoptotic process
**GO:0032731**	15	1.067	7.91E-06	7.41E-04	positive regulation of interleukin-1 beta production
**GO:0002717**	23	0.879	7.97E-06	7.44E-04	positive regulation of natural killer cell mediated immunity
**GO:0043500**	106	0.436	8.15E-06	7.60E-04	muscle adaptation
**GO:2001204**	11	1.152	8.28E-06	7.68E-04	regulation of osteoclast development
**GO:0034340**	70	0.538	8.61E-06	7.98E-04	response to type I interferon
**GO:0002456**	89	0.479	8.67E-06	8.01E-04	T cell mediated immunity
**GO:0070304**	166	0.348	8.69E-06	8.02E-04	positive regulation of stress-activated protein kinase signaling cascade
**GO:2001236**	149	0.368	8.74E-06	8.04E-04	regulation of extrinsic apoptotic signaling pathway
**GO:0032606**	117	0.422	8.73E-06	8.04E-04	type I interferon production
**GO:0050830**	67	0.547	8.75E-06	8.04E-04	defense response to Gram-positive bacterium
**GO:0071229**	75	0.519	8.80E-06	8.07E-04	cellular response to acid chemical
**GO:0014911**	37	0.708	9.17E-06	8.35E-04	positive regulation of smooth muscle cell migration
**GO:0071230**	68	0.544	9.25E-06	8.42E-04	cellular response to amino acid stimulus
**GO:0061448**	263	0.275	9.40E-06	8.52E-04	connective tissue development
**GO:0062207**	83	0.505	9.57E-06	8.67E-04	regulation of pattern recognition receptor signaling pathway
**GO:0018210**	122	0.410	9.65E-06	8.72E-04	peptidyl-threonine modification
**GO:0032528**	24	0.921	9.65E-06	8.72E-04	microvillus organization
**GO:0007043**	126	0.395	9.86E-06	8.89E-04	cell-cell junction assembly
**GO:0001938**	92	0.460	9.90E-06	8.92E-04	positive regulation of endothelial cell proliferation
**GO:0001822**	280	0.266	1.01E-05	9.09E-04	kidney development
**GO:0051988**	12	1.316	1.01E-05	9.09E-04	regulation of attachment of spindle microtubules to kinetochore
**GO:0002279**	46	0.658	1.02E-05	9.12E-04	mast cell activation involved in immune response

**GO:0052126**	150	0.363	1.02E-05	9.14E-04	movement in host environment
**GO:2000045**	145	0.372	1.03E-05	9.21E-04	regulation of G1/S transition of mitotic cell cycle
**GO:0045954**	22	0.891	1.05E-05	9.33E-04	positive regulation of natural killer cell mediated cytotoxicity
**GO:0006470**	305	0.255	1.06E-05	9.38E-04	protein dephosphorylation
**GO:0050922**	54	0.601	1.07E-05	9.52E-04	negative regulation of chemotaxis
**GO:0090287**	263	0.273	1.10E-05	9.76E-04	regulation of cellular response to growth factor stimulus
**GO:0032479**	115	0.420	1.14E-05	1.00E-03	regulation of type I interferon production
**GO:0002824**	89	0.477	1.15E-05	1.02E-03	positive regulation of adaptive immune response based on somatic recombination of immune receptors built from immunoglobulin superfamily domains
**GO:0032613**	38	0.704	1.21E-05	1.06E-03	interleukin-10 production
**GO:0051222**	340	0.240	1.22E-05	1.08E-03	positive regulation of protein transport
**GO:1901990**	399	0.222	1.23E-05	1.08E-03	regulation of mitotic cell cycle phase transition
**GO:0050777**	129	0.390	1.23E-05	1.08E-03	negative regulation of immune response
**GO:0071634**	35	0.721	1.23E-05	1.08E-03	regulation of transforming growth factor beta production
**GO:0072001**	296	0.256	1.24E-05	1.08E-03	renal system development
**GO:0045927**	251	0.279	1.24E-05	1.08E-03	positive regulation of growth
**GO:0001837**	133	0.382	1.24E-05	1.08E-03	epithelial to mesenchymal transition
**GO:0002791**	347	0.237	1.27E-05	1.11E-03	regulation of peptide secretion
**GO:0032760**	50	0.657	1.29E-05	1.12E-03	positive regulation of tumor necrosis factor production
**GO:0022406**	177	0.332	1.36E-05	1.18E-03	membrane docking
**GO:0071692**	422	0.215	1.37E-05	1.19E-03	protein localization to extracellular region
**GO:0001954**	50	0.616	1.37E-05	1.19E-03	positive regulation of cell-matrix adhesion
**GO:0002790**	454	0.207	1.38E-05	1.19E-03	peptide secretion
**GO:0032878**	25	0.889	1.38E-05	1.19E-03	regulation of establishment or maintenance of cell polarity
**GO:0060401**	172	0.335	1.39E-05	1.20E-03	cytosolic calcium ion transport
**GO:0030199**	50	0.634	1.42E-05	1.22E-03	collagen fibril organization
**GO:0001913**	35	0.760	1.43E-05	1.23E-03	T cell mediated cytotoxicity
**GO:0001763**	195	0.312	1.44E-05	1.23E-03	morphogenesis of a branching structure
**GO:0032874**	165	0.340	1.46E-05	1.25E-03	positive regulation of stress-activated MAPK cascade

**GO:0034599**	280	0.262	1.48E-05	1.26E-03	cellular response to oxidative stress
**GO:1901136**	177	0.328	1.51E-05	1.29E-03	carbohydrate derivative catabolic process
**GO:1905477**	123	0.399	1.52E-05	1.29E-03	positive regulation of protein localization to membrane
**GO:0002478**	149	0.360	1.54E-05	1.31E-03	antigen processing and presentation of exogenous peptide antigen
**GO:0019933**	171	0.332	1.55E-05	1.31E-03	cAMP-mediated signaling
**GO:0009595**	28	0.815	1.55E-05	1.31E-03	detection of biotic stimulus
**GO:0042552**	130	0.383	1.58E-05	1.34E-03	myelination
**GO:0045670**	62	0.560	1.61E-05	1.36E-03	regulation of osteoclast differentiation
**GO:0097306**	84	0.478	1.62E-05	1.37E-03	cellular response to alcohol
**GO:0097581**	79	0.492	1.65E-05	1.39E-03	lamellipodium organization
**GO:0002761**	113	0.410	1.68E-05	1.42E-03	regulation of myeloid leukocyte differentiation
**GO:0032869**	216	0.295	1.74E-05	1.46E-03	cellular response to insulin stimulus
**GO:0007009**	99	0.436	1.75E-05	1.47E-03	plasma membrane organization
**GO:1904951**	356	0.230	1.77E-05	1.48E-03	positive regulation of establishment of protein localization
**GO:0001101**	131	0.377	1.77E-05	1.48E-03	response to acid chemical
**GO:0051048**	176	0.327	1.78E-05	1.49E-03	negative regulation of secretion
**GO:0051216**	202	0.303	1.82E-05	1.51E-03	cartilage development
**GO:0017015**	110	0.412	1.82E-05	1.51E-03	regulation of transforming growth factor beta receptor signaling pathway
**GO:0010522**	99	0.434	1.81E-05	1.51E-03	regulation of calcium ion transport into cytosol
**GO:1903844**	112	0.408	1.84E-05	1.53E-03	regulation of cellular response to transforming growth factor beta stimulus
**GO:0010506**	317	0.243	1.86E-05	1.54E-03	regulation of autophagy
**GO:0032963**	98	0.438	1.87E-05	1.54E-03	collagen metabolic process
**GO:1990089**	55	0.583	1.87E-05	1.54E-03	response to nerve growth factor
**GO:0046640**	28	0.783	1.86E-05	1.54E-03	regulation of alpha-beta T cell proliferation
**GO:0048144**	80	0.483	1.88E-05	1.55E-03	fibroblast proliferation
**GO:0051494**	134	0.377	1.90E-05	1.56E-03	negative regulation of cytoskeleton organization
**GO:0021543**	166	0.333	1.96E-05	1.61E-03	pallium development
**GO:0038065**	13	1.023	2.11E-05	1.73E-03	collagen-activated signaling pathway
**GO:0060996**	93	0.443	2.14E-05	1.74E-03	dendritic spine development

**GO:0050857**	22	0.882	2.13E-05	1.74E-03	positive regulation of antigen receptor-mediated signaling pathway
**GO:0006979**	422	0.210	2.15E-05	1.75E-03	response to oxidative stress
**GO:0090316**	177	0.323	2.23E-05	1.82E-03	positive regulation of intracellular protein transport
**GO:0050803**	224	0.285	2.27E-05	1.84E-03	regulation of synapse structure or activity
**GO:0007272**	132	0.372	2.35E-05	1.91E-03	ensheathment of neurons
**GO:0008366**	132	0.372	2.35E-05	1.91E-03	axon ensheathment
**GO:0070266**	35	0.741	2.39E-05	1.93E-03	necroptotic process
**GO:0046330**	138	0.362	2.42E-05	1.95E-03	positive regulation of JNK cascade
**GO:0032653**	37	0.689	2.45E-05	1.97E-03	regulation of interleukin-10 production
**GO:0071352**	13	1.011	2.45E-05	1.97E-03	cellular response to interleukin-2
**GO:0070228**	50	0.623	2.48E-05	1.99E-03	regulation of lymphocyte apoptotic process
**GO:0072659**	260	0.264	2.49E-05	2.00E-03	protein localization to plasma membrane
**GO:0006936**	345	0.229	2.55E-05	2.04E-03	muscle contraction
**GO:0002011**	52	0.573	2.55E-05	2.04E-03	morphogenesis of an epithelial sheet
**GO:1903532**	298	0.246	2.57E-05	2.05E-03	positive regulation of secretion by cell
**GO:0001933**	388	0.216	2.59E-05	2.06E-03	negative regulation of protein phosphorylation
**GO:0016358**	230	0.280	2.62E-05	2.09E-03	dendrite development
**GO:0051099**	179	0.317	2.65E-05	2.10E-03	positive regulation of binding
**GO:0001780**	17	1.028	2.68E-05	2.12E-03	neutrophil homeostasis
**GO:0019883**	14	1.046	2.69E-05	2.13E-03	antigen processing and presentation of endogenous antigen
**GO:1904062**	325	0.235	2.79E-05	2.21E-03	regulation of cation transmembrane transport
**GO:0032570**	46	0.605	2.81E-05	2.22E-03	response to progesterone
**GO:0034110**	25	0.807	2.88E-05	2.27E-03	regulation of homotypic cell-cell adhesion
**GO:0048145**	79	0.475	2.88E-05	2.27E-03	regulation of fibroblast proliferation
**GO:0050708**	317	0.238	2.89E-05	2.27E-03	regulation of protein secretion
**GO:0050807**	215	0.287	2.89E-05	2.27E-03	regulation of synapse organization
**GO:0042326**	425	0.206	2.93E-05	2.30E-03	negative regulation of phosphorylation
**GO:0070588**	307	0.241	2.95E-05	2.32E-03	calcium ion transmembrane transport
**GO:0051770**	15	1.197	2.96E-05	2.32E-03	positive regulation of nitric-oxide synthase biosynthetic process

**GO:0032648**	41	0.707	2.96E-05	2.32E-03	regulation of interferon-beta production
**GO:1903169**	144	0.352	2.99E-05	2.34E-03	regulation of calcium ion transmembrane transport
**GO:1901224**	75	0.493	3.02E-05	2.36E-03	positive regulation of NIK/NF-kappaB signaling
**GO:0070527**	59	0.544	3.04E-05	2.37E-03	platelet aggregation
**GO:0014015**	75	0.488	3.07E-05	2.38E-03	positive regulation of gliogenesis
**GO:1904377**	63	0.531	3.07E-05	2.38E-03	positive regulation of protein localization to cell periphery
**GO:1904019**	98	0.419	3.10E-05	2.41E-03	epithelial cell apoptotic process
**GO:0003170**	60	0.546	3.11E-05	2.41E-03	heart valve development
**GO:0002287**	60	0.543	3.16E-05	2.44E-03	alpha-beta T cell activation involved in immune response
**GO:0002293**	60	0.543	3.16E-05	2.44E-03	alpha-beta T cell differentiation involved in immune response
**GO:0035592**	417	0.206	3.19E-05	2.46E-03	establishment of protein localization to extracellular region
**GO:0032486**	14	1.080	3.20E-05	2.46E-03	Rap protein signal transduction
**GO:0048705**	236	0.272	3.30E-05	2.54E-03	skeletal system morphogenesis
**GO:0050931**	35	0.722	3.31E-05	2.54E-03	pigment cell differentiation
**GO:0043200**	112	0.395	3.32E-05	2.55E-03	response to amino acid
**GO:0036119**	21	0.878	3.34E-05	2.56E-03	response to platelet-derived growth factor
**GO:0009306**	416	0.206	3.37E-05	2.58E-03	protein secretion
**GO:0007091**	54	0.571	3.38E-05	2.58E-03	metaphase/anaphase transition of mitotic cell cycle
**GO:0010965**	54	0.571	3.38E-05	2.58E-03	regulation of mitotic sister chromatid separation
**GO:0071695**	224	0.279	3.40E-05	2.59E-03	anatomical structure maturation
**GO:0060337**	67	0.512	3.48E-05	2.64E-03	type I interferon signaling pathway
**GO:0071357**	67	0.512	3.48E-05	2.64E-03	cellular response to type I interferon
**GO:0002294**	59	0.545	3.50E-05	2.65E-03	CD4-positive, alpha-beta T cell differentiation involved in immune response
**GO:2000108**	25	0.828	3.50E-05	2.65E-03	positive regulation of leukocyte apoptotic process
**GO:0046638**	46	0.610	3.56E-05	2.69E-03	positive regulation of alpha-beta T cell differentiation
**GO:0051894**	25	0.783	3.58E-05	2.70E-03	positive regulation of focal adhesion assembly
**GO:0060562**	323	0.232	3.60E-05	2.72E-03	epithelial tube morphogenesis
**GO:0030889**	16	0.923	3.61E-05	2.72E-03	negative regulation of B cell proliferation
**GO:0051489**	43	0.620	3.62E-05	2.72E-03	regulation of filopodium assembly

**GO:0036092**	14	1.002	3.65E-05	2.74E-03	phosphatidylinositol-3-phosphate biosynthetic process
**GO:2000379**	94	0.432	3.66E-05	2.74E-03	positive regulation of reactive oxygen species metabolic process
**GO:0002688**	106	0.404	3.69E-05	2.76E-03	regulation of leukocyte chemotaxis
**GO:0070098**	77	0.478	3.70E-05	2.76E-03	chemokine-mediated signaling pathway
**GO:0033631**	16	0.959	3.71E-05	2.77E-03	cell-cell adhesion mediated by integrin
**GO:0043502**	83	0.454	3.72E-05	2.77E-03	regulation of muscle adaptation
**GO:0008544**	362	0.218	3.75E-05	2.79E-03	epidermis development
**GO:0060420**	65	0.510	3.78E-05	2.80E-03	regulation of heart growth
**GO:0007156**	119	0.377	3.83E-05	2.84E-03	homophilic cell adhesion via plasma membrane adhesion molecules
**GO:0003231**	126	0.365	3.94E-05	2.91E-03	cardiac ventricle development
**GO:0007062**	56	0.562	3.96E-05	2.92E-03	sister chromatid cohesion
**GO:0006333**	168	0.319	3.96E-05	2.92E-03	chromatin assembly or disassembly
**GO:0060252**	17	0.911	3.99E-05	2.93E-03	positive regulation of glial cell proliferation
**GO:0050860**	18	0.915	3.98E-05	2.93E-03	negative regulation of T cell receptor signaling pathway
**GO:0043370**	46	0.610	4.05E-05	2.97E-03	regulation of CD4-positive, alpha-beta T cell differentiation
**GO:0007178**	327	0.229	4.05E-05	2.97E-03	transmembrane receptor protein serine/threonine kinase signaling pathway
**GO:0150117**	26	0.762	4.08E-05	2.99E-03	positive regulation of cell-substrate junction organization
**GO:0032868**	273	0.251	4.09E-05	2.99E-03	response to insulin
**GO:0055024**	80	0.458	4.10E-05	3.00E-03	regulation of cardiac muscle tissue development
**GO:0006338**	185	0.305	4.19E-05	3.05E-03	chromatin remodeling
**GO:0002367**	82	0.465	4.22E-05	3.07E-03	cytokine production involved in immune response
**GO:1904705**	50	0.585	4.22E-05	3.07E-03	regulation of vascular associated smooth muscle cell proliferation
**GO:1990874**	50	0.585	4.22E-05	3.07E-03	vascular associated smooth muscle cell proliferation
**GO:0043506**	84	0.447	4.45E-05	3.22E-03	regulation of JUN kinase activity
**GO:0070227**	65	0.522	4.45E-05	3.22E-03	lymphocyte apoptotic process
**GO:1901184**	87	0.442	4.48E-05	3.24E-03	regulation of ERBB signaling pathway
**GO:0043331**	42	0.632	4.57E-05	3.30E-03	response to dsRNA
**GO:1905475**	187	0.303	4.59E-05	3.31E-03	regulation of protein localization to membrane

**GO:0048738**	208	0.285	4.60E-05	3.32E-03	cardiac muscle tissue development
**GO:0034332**	64	0.508	4.66E-05	3.35E-03	adherens junction organization
**GO:0001890**	153	0.330	4.75E-05	3.41E-03	placenta development
**GO:0032615**	39	0.661	4.77E-05	3.42E-03	interleukin-12 production
**GO:2000353**	18	0.894	4.77E-05	3.42E-03	positive regulation of endothelial cell apoptotic process
**GO:0036120**	20	0.887	4.84E-05	3.46E-03	cellular response to platelet-derived growth factor stimulus
**GO:0030890**	41	0.675	4.92E-05	3.51E-03	positive regulation of B cell proliferation
**GO:0002042**	54	0.552	4.93E-05	3.52E-03	cell migration involved in sprouting angiogenesis
**GO:0044319**	28	0.735	4.98E-05	3.54E-03	wound healing, spreading of cells
**GO:0090505**	28	0.735	4.98E-05	3.54E-03	epiboly involved in wound healing
**GO:0090504**	29	0.720	5.05E-05	3.58E-03	epiboly
**GO:1990778**	317	0.230	5.12E-05	3.62E-03	protein localization to cell periphery
**GO:0070301**	88	0.439	5.11E-05	3.62E-03	cellular response to hydrogen peroxide
**GO:0032386**	336	0.224	5.19E-05	3.67E-03	regulation of intracellular transport
**GO:0003208**	72	0.468	5.33E-05	3.76E-03	cardiac ventricle morphogenesis
**GO:0018107**	114	0.385	5.37E-05	3.79E-03	peptidyl-threonine phosphorylation
**GO:1903670**	67	0.493	5.37E-05	3.79E-03	regulation of sprouting angiogenesis
**GO:0051491**	30	0.702	5.40E-05	3.80E-03	positive regulation of filopodium assembly
**GO:0002262**	147	0.335	5.54E-05	3.89E-03	myeloid cell homeostasis
**GO:0043552**	33	0.677	5.60E-05	3.93E-03	positive regulation of phosphatidylinositol 3-kinase activity
**GO:0055021**	59	0.524	5.70E-05	3.99E-03	regulation of cardiac muscle tissue growth
**GO:0002448**	47	0.609	5.71E-05	4.00E-03	mast cell mediated immunity
**GO:0050871**	72	0.490	5.74E-05	4.01E-03	positive regulation of B cell activation
**GO:0050663**	61	0.516	5.79E-05	4.04E-03	cytokine secretion
**GO:0061138**	181	0.300	5.89E-05	4.11E-03	morphogenesis of a branching epithelium
**GO:0033157**	251	0.257	5.91E-05	4.11E-03	regulation of intracellular protein transport
**GO:0033028**	32	0.698	5.96E-05	4.15E-03	myeloid cell apoptotic process
**GO:0032755**	53	0.561	6.02E-05	4.18E-03	positive regulation of interleukin-6 production
**GO:0045665**	220	0.273	6.07E-05	4.21E-03	negative regulation of neuron differentiation

**GO:0060368**	12	1.193	6.08E-05	4.21E-03	regulation of Fc receptor mediated stimulatory signaling pathway
**GO:0034612**	281	0.241	6.23E-05	4.31E-03	response to tumor necrosis factor
**GO:1903510**	110	0.388	6.26E-05	4.33E-03	mucopolysaccharide metabolic process
**GO:1903522**	282	0.240	6.30E-05	4.35E-03	regulation of blood circulation
**GO:0036037**	22	0.781	6.39E-05	4.41E-03	CD8-positive, alpha-beta T cell activation
**GO:0042698**	71	0.469	6.44E-05	4.43E-03	ovulation cycle
**GO:0042771**	45	0.592	6.45E-05	4.44E-03	intrinsic apoptotic signaling pathway in response to DNA damage by p53 class mediator
**GO:0036035**	17	0.948	6.63E-05	4.56E-03	osteoclast development
**GO:0070102**	19	0.884	6.67E-05	4.58E-03	interleukin-6-mediated signaling pathway
**GO:0060249**	405	0.201	6.68E-05	4.58E-03	anatomical structure homeostasis
**GO:0052548**	358	0.213	6.79E-05	4.65E-03	regulation of endopeptidase activity
**GO:0030203**	155	0.323	6.81E-05	4.66E-03	glycosaminoglycan metabolic process
**GO:0052547**	383	0.206	6.86E-05	4.69E-03	regulation of peptidase activity
**GO:0071786**	16	0.920	6.90E-05	4.71E-03	endoplasmic reticulum tubular network organization
**GO:0034614**	156	0.321	6.94E-05	4.73E-03	cellular response to reactive oxygen species
**GO:0051492**	80	0.449	7.00E-05	4.77E-03	regulation of stress fiber assembly
**GO:2000134**	100	0.405	7.10E-05	4.83E-03	negative regulation of G1/S transition of mitotic cell cycle
**GO:0050690**	30	0.727	7.21E-05	4.90E-03	regulation of defense response to virus by virus
**GO:0033077**	71	0.476	7.31E-05	4.96E-03	T cell differentiation in thymus
**GO:0042692**	346	0.216	7.34E-05	4.97E-03	muscle cell differentiation
**GO:1901215**	204	0.279	7.38E-05	4.99E-03	negative regulation of neuron death
**GO:0071675**	43	0.600	7.41E-05	5.01E-03	regulation of mononuclear cell migration
**GO:0016572**	38	0.635	7.49E-05	5.05E-03	histone phosphorylation
**GO:0008608**	32	0.750	7.49E-05	5.05E-03	attachment of spindle microtubules to kinetochore
**GO:0048844**	64	0.492	7.53E-05	5.07E-03	artery morphogenesis
**GO:0048247**	50	0.563	7.56E-05	5.08E-03	lymphocyte chemotaxis
**GO:0050852**	176	0.302	7.58E-05	5.09E-03	T cell receptor signaling pathway
**GO:0014013**	123	0.360	7.63E-05	5.12E-03	regulation of gliogenesis
**GO:0001701**	346	0.215	7.71E-05	5.16E-03	in utero embryonic development

**GO:0006925**	21	0.875	7.72E-05	5.16E-03	inflammatory cell apoptotic process
**GO:0001885**	60	0.516	7.78E-05	5.18E-03	endothelial cell development
**GO:0003205**	166	0.308	7.84E-05	5.22E-03	cardiac chamber development
**GO:0120034**	99	0.397	7.95E-05	5.28E-03	positive regulation of plasma membrane bounded cell projection assembly
**GO:0002686**	42	0.622	8.08E-05	5.37E-03	negative regulation of leukocyte migration
**GO:0006809**	68	0.483	8.16E-05	5.42E-03	nitric oxide biosynthetic process
**GO:0031668**	231	0.262	8.22E-05	5.45E-03	cellular response to extracellular stimulus
**GO:1902904**	127	0.355	8.32E-05	5.51E-03	negative regulation of supramolecular fiber organization
**GO:0048880**	371	0.206	8.49E-05	5.61E-03	sensory system development
**GO:0050901**	25	0.748	8.51E-05	5.62E-03	leukocyte tethering or rolling
**GO:1904037**	30	0.689	8.57E-05	5.65E-03	positive regulation of epithelial cell apoptotic process
**GO:1903078**	56	0.532	8.68E-05	5.72E-03	positive regulation of protein localization to plasma membrane
**GO:0042058**	80	0.444	8.73E-05	5.74E-03	regulation of epidermal growth factor receptor signaling pathway
**GO:0033628**	46	0.589	8.74E-05	5.74E-03	regulation of cell adhesion mediated by integrin
**GO:0072006**	143	0.331	8.77E-05	5.74E-03	nephron development
**GO:0006027**	61	0.509	8.79E-05	5.74E-03	glycosaminoglycan catabolic process
**GO:0014009**	50	0.559	8.78E-05	5.74E-03	glial cell proliferation
**GO:1904407**	41	0.621	8.90E-05	5.81E-03	positive regulation of nitric oxide metabolic process
**GO:0060419**	87	0.426	8.95E-05	5.83E-03	heart growth
**GO:0006929**	26	0.759	8.95E-05	5.83E-03	substrate-dependent cell migration
**GO:0032231**	93	0.411	9.01E-05	5.86E-03	regulation of actin filament bundle assembly
**GO:0001570**	77	0.443	9.25E-05	6.01E-03	vasculogenesis
**GO:0150063**	366	0.206	9.69E-05	6.28E-03	visual system development
**GO:0045069**	80	0.440	9.68E-05	6.28E-03	regulation of viral genome replication
**GO:0030856**	137	0.335	9.73E-05	6.30E-03	regulation of epithelial cell differentiation
**GO:1990253**	10	1.099	9.81E-05	6.34E-03	cellular response to leucine starvation
**GO:1905564**	14	0.942	9.83E-05	6.35E-03	positive regulation of vascular endothelial cell proliferation
**GO:0009896**	407	0.195	9.85E-05	6.35E-03	positive regulation of catabolic process
**GO:0034504**	251	0.248	1.00E-04	6.45E-03	protein localization to nucleus

**GO:0021987**	110	0.371	1.01E-04	6.47E-03	cerebral cortex development
**GO:0002507**	22	0.837	1.01E-04	6.51E-03	tolerance induction
**GO:0046209**	73	0.461	1.03E-04	6.63E-03	nitric oxide metabolic process
**GO:0007548**	263	0.241	1.05E-04	6.73E-03	sex differentiation
**GO:0007584**	177	0.294	1.05E-04	6.74E-03	response to nutrient
**GO:0043201**	13	1.029	1.05E-04	6.75E-03	response to leucine
**GO:0010717**	82	0.436	1.06E-04	6.78E-03	regulation of epithelial to mesenchymal transition
**GO:0060043**	41	0.608	1.06E-04	6.78E-03	regulation of cardiac muscle cell proliferation
**GO:0001649**	206	0.272	1.07E-04	6.84E-03	osteoblast differentiation
**GO:1903707**	132	0.340	1.07E-04	6.84E-03	negative regulation of hemopoiesis
**GO:0034105**	32	0.660	1.07E-04	6.84E-03	positive regulation of tissue remodeling
**GO:0010639**	367	0.205	1.08E-04	6.87E-03	negative regulation of organelle organization
**GO:1902743**	42	0.595	1.10E-04	6.97E-03	regulation of lamellipodium organization
**GO:0070168**	27	0.739	1.13E-04	7.14E-03	negative regulation of biomineral tissue development
**GO:0110150**	27	0.739	1.13E-04	7.14E-03	negative regulation of biomineralization
**GO:2000116**	219	0.264	1.13E-04	7.16E-03	regulation of cysteine-type endopeptidase activity
**GO:0022617**	74	0.453	1.14E-04	7.22E-03	extracellular matrix disassembly
**GO:0006906**	94	0.405	1.15E-04	7.28E-03	vesicle fusion
**GO:1902806**	158	0.310	1.16E-04	7.33E-03	regulation of cell cycle G1/S phase transition
**GO:0001654**	362	0.205	1.16E-04	7.33E-03	eye development
**GO:0046620**	95	0.397	1.17E-04	7.34E-03	regulation of organ growth
**GO:1902807**	102	0.388	1.17E-04	7.35E-03	negative regulation of cell cycle G1/S phase transition
**GO:1903531**	152	0.316	1.19E-04	7.43E-03	negative regulation of secretion by cell
**GO:0002700**	119	0.359	1.19E-04	7.43E-03	regulation of production of molecular mediator of immune response
**GO:0042306**	58	0.509	1.18E-04	7.43E-03	regulation of protein import into nucleus
**GO:0042093**	57	0.514	1.21E-04	7.56E-03	T-helper cell differentiation
**GO:0030071**	51	0.543	1.22E-04	7.64E-03	regulation of mitotic metaphase/anaphase transition
**GO:2000107**	45	0.587	1.23E-04	7.70E-03	negative regulation of leukocyte apoptotic process
**GO:0031331**	345	0.209	1.24E-04	7.75E-03	positive regulation of cellular catabolic process
**GO:0032655**	37	0.642	1.25E-04	7.78E-03	regulation of interleukin-12 production

**GO:0051279**	78	0.440	1.27E-04	7.91E-03	regulation of release of sequestered calcium ion into cytosol
**GO:0003007**	248	0.245	1.29E-04	7.98E-03	heart morphogenesis
**GO:0045137**	217	0.262	1.29E-04	7.98E-03	development of primary sexual characteristics
**GO:0032355**	133	0.334	1.29E-04	8.00E-03	response to estradiol
**GO:0006939**	103	0.379	1.29E-04	8.00E-03	smooth muscle contraction
**GO:0031960**	160	0.305	1.30E-04	8.03E-03	response to corticosteroid
**GO:0050688**	66	0.480	1.31E-04	8.12E-03	regulation of defense response to virus
**GO:0046849**	91	0.404	1.33E-04	8.19E-03	bone remodeling
**GO:0042130**	53	0.530	1.36E-04	8.40E-03	negative regulation of T cell proliferation
**GO:0032740**	14	0.923	1.38E-04	8.48E-03	positive regulation of interleukin-17 production
**GO:0030278**	187	0.281	1.39E-04	8.55E-03	regulation of ossification
**GO:0061383**	49	0.534	1.40E-04	8.59E-03	trabecula morphogenesis
**GO:2001237**	99	0.387	1.40E-04	8.60E-03	negative regulation of extrinsic apoptotic signaling pathway
**GO:0140056**	168	0.297	1.41E-04	8.62E-03	organelle localization by membrane tethering
**GO:0070672**	14	0.903	1.41E-04	8.63E-03	response to interleukin-15
**GO:0006509**	43	0.642	1.42E-04	8.69E-03	membrane protein ectodomain proteolysis
**GO:0007565**	183	0.283	1.43E-04	8.72E-03	female pregnancy
**GO:0030048**	137	0.328	1.43E-04	8.73E-03	actin filament-based movement
**GO:0043281**	196	0.275	1.44E-04	8.75E-03	regulation of cysteine-type endopeptidase activity involved in apoptotic process
**GO:0071233**	10	1.134	1.44E-04	8.75E-03	cellular response to leucine
**GO:0043393**	209	0.265	1.44E-04	8.77E-03	regulation of protein binding
**GO:0033046**	41	0.604	1.45E-04	8.81E-03	negative regulation of sister chromatid segregation
**GO:0031650**	13	0.909	1.46E-04	8.84E-03	regulation of heat generation
**GO:0097696**	139	0.325	1.53E-04	9.25E-03	receptor signaling pathway via STAT
**GO:0097300**	42	0.605	1.53E-04	9.27E-03	programmed necrotic cell death
**GO:0055017**	80	0.430	1.55E-04	9.33E-03	cardiac muscle tissue growth
**GO:1901987**	430	0.185	1.55E-04	9.36E-03	regulation of cell cycle phase transition
**GO:0051985**	42	0.592	1.56E-04	9.41E-03	negative regulation of chromosome segregation
**GO:0045429**	40	0.606	1.57E-04	9.46E-03	positive regulation of nitric oxide biosynthetic process
**GO:0061097**	88	0.406	1.58E-04	9.50E-03	regulation of protein tyrosine kinase activity
**GO:0002756**	33	0.646	1.61E-04	9.65E-03	MyD88-independent toll-like receptor signaling pathway

**GO:0097242**	34	0.659	1.61E-04	9.65E-03	amyloid-beta clearance
**GO:1901879**	80	0.431	1.62E-04	9.70E-03	regulation of protein depolymerization
**GO:0006959**	190	0.277	1.64E-04	9.83E-03	humoral immune response
**GO:0007259**	135	0.326	1.71E-04	1.02E-02	receptor signaling pathway via JAK-STAT
**GO:1905818**	59	0.499	1.72E-04	1.03E-02	regulation of chromosome separation
**GO:0016055**	499	0.170	1.73E-04	1.03E-02	Wnt signaling pathway
**GO:0032728**	25	0.788	1.73E-04	1.03E-02	positive regulation of interferon-beta production
**GO:0070507**	180	0.283	1.73E-04	1.03E-02	regulation of microtubule cytoskeleton organization
**GO:0042307**	39	0.596	1.77E-04	1.06E-02	positive regulation of protein import into nucleus
**GO:0014855**	64	0.479	1.78E-04	1.06E-02	striated muscle cell proliferation
**GO:0032465**	82	0.418	1.83E-04	1.08E-02	regulation of cytokinesis
**GO:1901989**	91	0.402	1.86E-04	1.10E-02	positive regulation of cell cycle phase transition
**GO:0007292**	131	0.328	1.88E-04	1.11E-02	female gamete generation
**GO:0046850**	50	0.512	1.88E-04	1.11E-02	regulation of bone remodeling
**GO:0032495**	20	0.914	1.90E-04	1.12E-02	response to muramyl dipeptide
**GO:0043270**	265	0.231	1.91E-04	1.13E-02	positive regulation of ion transport
**GO:0043030**	55	0.502	1.91E-04	1.13E-02	regulation of macrophage activation
**GO:0002371**	11	1.083	1.93E-04	1.13E-02	dendritic cell cytokine production
**GO:0033003**	39	0.608	1.94E-04	1.14E-02	regulation of mast cell activation
**GO:0048568**	429	0.182	1.96E-04	1.15E-02	embryonic organ development
**GO:0071356**	263	0.232	1.97E-04	1.16E-02	cellular response to tumor necrosis factor
**GO:0003206**	125	0.332	1.98E-04	1.16E-02	cardiac chamber morphogenesis
**GO:0010742**	33	0.647	1.99E-04	1.16E-02	macrophage derived foam cell differentiation
**GO:0090077**	33	0.647	1.99E-04	1.16E-02	foam cell differentiation
**GO:0003179**	51	0.530	1.99E-04	1.16E-02	heart valve morphogenesis
**GO:0060135**	65	0.461	2.00E-04	1.17E-02	maternal process involved in female pregnancy
**GO:0060251**	33	0.624	2.03E-04	1.18E-02	regulation of glial cell proliferation
**GO:1904375**	115	0.351	2.06E-04	1.20E-02	regulation of protein localization to cell periphery
**GO:2000404**	41	0.595	2.06E-04	1.20E-02	regulation of T cell migration
**GO:0048813**	142	0.314	2.06E-04	1.20E-02	dendrite morphogenesis
**GO:0061437**	26	0.699	2.07E-04	1.20E-02	renal system vasculature development
**GO:0061440**	26	0.699	2.07E-04	1.20E-02	kidney vasculature development

**GO:0046641**	19	0.819	2.07E-04	1.20E-02	positive regulation of alpha-beta T cell proliferation
**GO:0007189**	127	0.330	2.08E-04	1.21E-02	adenylate cyclase-activating G protein-coupled receptor signaling pathway
**GO:0002698**	103	0.368	2.08E-04	1.21E-02	negative regulation of immune effector process
**GO:0085029**	37	0.622	2.09E-04	1.21E-02	extracellular matrix assembly
**GO:1902041**	54	0.516	2.11E-04	1.22E-02	regulation of extrinsic apoptotic signaling pathway via death domain receptors
**GO:2001057**	76	0.430	2.11E-04	1.22E-02	reactive nitrogen species metabolic process
**GO:0002691**	28	0.709	2.12E-04	1.22E-02	regulation of cellular extravasation
**GO:0010762**	30	0.670	2.13E-04	1.23E-02	regulation of fibroblast migration
**GO:0002576**	125	0.334	2.13E-04	1.23E-02	platelet degranulation
**GO:0090594**	11	0.985	2.14E-04	1.23E-02	inflammatory response to wounding
**GO:1903727**	49	0.534	2.15E-04	1.24E-02	positive regulation of phospholipid metabolic process
**GO:0003229**	55	0.490	2.16E-04	1.24E-02	ventricular cardiac muscle tissue development
**GO:0033619**	60	0.506	2.18E-04	1.25E-02	membrane protein proteolysis
**GO:0044784**	56	0.504	2.27E-04	1.30E-02	metaphase/anaphase transition of cell cycle
**GO:0051767**	19	0.919	2.29E-04	1.31E-02	nitric-oxide synthase biosynthetic process
**GO:0051769**	19	0.919	2.29E-04	1.31E-02	regulation of nitric-oxide synthase biosynthetic process
**GO:0070757**	11	1.001	2.29E-04	1.31E-02	interleukin-35-mediated signaling pathway
**GO:0006022**	164	0.290	2.30E-04	1.31E-02	aminoglycan metabolic process
**GO:0009913**	257	0.231	2.31E-04	1.31E-02	epidermal cell differentiation
**GO:0022612**	123	0.334	2.32E-04	1.32E-02	gland morphogenesis
**GO:0051225**	99	0.378	2.33E-04	1.32E-02	spindle assembly
**GO:0016050**	320	0.208	2.35E-04	1.34E-02	vesicle organization
**GO:0060349**	114	0.345	2.39E-04	1.36E-02	bone morphogenesis
**GO:0043116**	14	0.849	2.43E-04	1.37E-02	negative regulation of vascular permeability
**GO:0003015**	279	0.221	2.49E-04	1.40E-02	heart process
**GO:0043647**	71	0.436	2.49E-04	1.40E-02	inositol phosphate metabolic process
**GO:0030318**	26	0.743	2.49E-04	1.40E-02	melanocyte differentiation
**GO:1903426**	84	0.403	2.53E-04	1.42E-02	regulation of reactive oxygen species biosynthetic process
**GO:0048146**	48	0.532	2.53E-04	1.42E-02	positive regulation of fibroblast proliferation

**GO:0002755**	35	0.667	2.53E-04	1.42E-02	MyD88-dependent toll-like receptor signaling pathway
**GO:0050707**	48	0.526	2.56E-04	1.44E-02	regulation of cytokine secretion
**GO:0021955**	35	0.618	2.56E-04	1.44E-02	central nervous system neuron axonogenesis
**GO:1901186**	31	0.661	2.58E-04	1.45E-02	positive regulation of ERBB signaling pathway
**GO:0010591**	30	0.666	2.60E-04	1.45E-02	regulation of lamellipodium assembly
**GO:0038110**	12	0.921	2.62E-04	1.46E-02	interleukin-2-mediated signaling pathway
**GO:0031103**	48	0.522	2.62E-04	1.46E-02	axon regeneration
**GO:0006869**	337	0.201	2.65E-04	1.47E-02	lipid transport
**GO:0050000**	76	0.425	2.67E-04	1.48E-02	chromosome localization
**GO:0007569**	100	0.370	2.68E-04	1.49E-02	cell aging
**GO:1903428**	50	0.523	2.68E-04	1.49E-02	positive regulation of reactive oxygen species biosynthetic process
**GO:0007143**	30	0.665	2.76E-04	1.53E-02	female meiotic nuclear division
**GO:0044706**	211	0.252	2.80E-04	1.55E-02	multi-multicellular organism process
**GO:0001911**	12	0.915	2.83E-04	1.57E-02	negative regulation of leukocyte mediated cytotoxicity
**GO:0071103**	280	0.219	2.85E-04	1.58E-02	DNA conformation change
**GO:0003222**	16	0.823	2.85E-04	1.58E-02	ventricular trabecula myocardium morphogenesis
**GO:0032620**	26	0.701	2.86E-04	1.58E-02	interleukin-17 production
**GO:0001914**	24	0.736	2.86E-04	1.58E-02	regulation of T cell mediated cytotoxicity
**GO:0061756**	35	0.630	2.95E-04	1.63E-02	leukocyte adhesion to vascular endothelial cell
**GO:0055010**	48	0.507	2.96E-04	1.63E-02	ventricular cardiac muscle tissue morphogenesis
**GO:0035666**	29	0.666	2.96E-04	1.63E-02	TRIF-dependent toll-like receptor signaling pathway
**GO:0071354**	36	0.595	3.02E-04	1.66E-02	cellular response to interleukin-6
**GO:0070167**	91	0.379	3.05E-04	1.68E-02	regulation of biomineral tissue development
**GO:0110149**	91	0.379	3.05E-04	1.68E-02	regulation of biomineralization
**GO:0045616**	39	0.568	3.06E-04	1.68E-02	regulation of keratinocyte differentiation
**GO:1901880**	63	0.468	3.08E-04	1.68E-02	negative regulation of protein depolymerization
**GO:0036473**	80	0.412	3.09E-04	1.69E-02	cell death in response to oxidative stress
**GO:0010592**	20	0.808	3.11E-04	1.70E-02	positive regulation of lamellipodium assembly
**GO:0031214**	156	0.290	3.13E-04	1.71E-02	biomineral tissue development
**GO:0110148**	156	0.290	3.13E-04	1.71E-02	biomineralization

**GO:0031663**	54	0.513	3.13E-04	1.71E-02	lipopolysaccharide-mediated signaling pathway
**GO:0061384**	34	0.598	3.15E-04	1.71E-02	heart trabecula morphogenesis
**GO:1904646**	35	0.652	3.15E-04	1.71E-02	cellular response to amyloid-beta
**GO:0051303**	75	0.423	3.15E-04	1.71E-02	establishment of chromosome localization
**GO:0016239**	65	0.452	3.22E-04	1.75E-02	positive regulation of macroautophagy
**GO:0032886**	224	0.243	3.23E-04	1.75E-02	regulation of microtubule-based process
**GO:0098543**	13	1.089	3.24E-04	1.75E-02	detection of other organism
**GO:0002718**	63	0.468	3.24E-04	1.75E-02	regulation of cytokine production involved in immune response
**GO:0010596**	49	0.510	3.31E-04	1.79E-02	negative regulation of endothelial cell migration
**GO:0007077**	12	1.079	3.33E-04	1.80E-02	mitotic nuclear envelope disassembly
**GO:0045124**	42	0.537	3.36E-04	1.81E-02	regulation of bone resorption
**GO:0061037**	26	0.685	3.44E-04	1.85E-02	negative regulation of cartilage development
**GO:0019048**	31	0.626	3.45E-04	1.86E-02	modulation by virus of host process
**GO:0106027**	84	0.394	3.47E-04	1.87E-02	neuron projection organization
**GO:0032660**	24	0.721	3.50E-04	1.88E-02	regulation of interleukin-17 production
**GO:0048066**	45	0.538	3.51E-04	1.89E-02	developmental pigmentation
**GO:0008406**	211	0.247	3.53E-04	1.89E-02	gonad development
**GO:1900407**	73	0.424	3.53E-04	1.89E-02	regulation of cellular response to oxidative stress
**GO:0031623**	107	0.346	3.57E-04	1.91E-02	receptor internalization
**GO:1900182**	75	0.416	3.59E-04	1.92E-02	positive regulation of protein localization to nucleus
**GO:0051306**	57	0.479	3.61E-04	1.93E-02	mitotic sister chromatid separation
**GO:0010761**	37	0.591	3.63E-04	1.93E-02	fibroblast migration
**GO:0034694**	31	0.636	3.63E-04	1.93E-02	response to prostaglandin
**GO:0071361**	13	0.932	3.64E-04	1.94E-02	cellular response to ethanol
**GO:0006323**	187	0.263	3.66E-04	1.94E-02	DNA packaging
**GO:0001558**	396	0.181	3.67E-04	1.95E-02	regulation of cell growth
**GO:0043242**	70	0.435	3.69E-04	1.96E-02	negative regulation of protein-containing complex disassembly
**GO:0048008**	55	0.489	3.71E-04	1.97E-02	platelet-derived growth factor receptor signaling pathway
**GO:0010508**	117	0.330	3.77E-04	1.99E-02	positive regulation of autophagy
**GO:0008630**	103	0.351	3.81E-04	2.02E-02	intrinsic apoptotic signaling pathway in response to DNA damage
**GO:0010256**	413	0.177	3.83E-04	2.02E-02	endomembrane system organization

**GO:0030100**	197	0.254	3.88E-04	2.05E-02	regulation of endocytosis
**GO:1903201**	62	0.457	3.93E-04	2.07E-02	regulation of oxidative stress-induced cell death
**GO:0045010**	44	0.561	4.01E-04	2.11E-02	actin nucleation
**GO:0035313**	15	0.865	4.04E-04	2.13E-02	wound healing, spreading of epidermal cells
**GO:0003018**	161	0.280	4.07E-04	2.14E-02	vascular process in circulatory system
**GO:0061098**	55	0.480	4.09E-04	2.15E-02	positive regulation of protein tyrosine kinase activity
**GO:0070106**	11	1.051	4.14E-04	2.17E-02	interleukin-27-mediated signaling pathway
**GO:0001660**	10	0.975	4.15E-04	2.17E-02	fever generation
**GO:0072012**	24	0.694	4.16E-04	2.18E-02	glomerulus vasculature development
**GO:0003418**	24	0.684	4.18E-04	2.18E-02	growth plate cartilage chondrocyte differentiation
**GO:0051016**	19	0.811	4.19E-04	2.19E-02	barbed-end actin filament capping
**GO:0006937**	157	0.283	4.22E-04	2.20E-02	regulation of muscle contraction
**GO:0070306**	31	0.635	4.24E-04	2.21E-02	lens fiber cell differentiation
**GO:0010952**	172	0.271	4.25E-04	2.21E-02	positive regulation of peptidase activity
**GO:0006026**	65	0.441	4.25E-04	2.21E-02	aminoglycan catabolic process
**GO:0051385**	31	0.645	4.29E-04	2.23E-02	response to mineralocorticoid
**GO:0043010**	321	0.198	4.30E-04	2.23E-02	camera-type eye development
**GO:0010611**	56	0.478	4.34E-04	2.25E-02	regulation of cardiac muscle hypertrophy
**GO:1903900**	128	0.314	4.36E-04	2.26E-02	regulation of viral life cycle
**GO:0032958**	42	0.539	4.39E-04	2.28E-02	inositol phosphate biosynthetic process
**GO:0001773**	26	0.739	4.41E-04	2.29E-02	myeloid dendritic cell activation
**GO:1903978**	21	0.739	4.45E-04	2.30E-02	regulation of microglial cell activation
**GO:0038061**	161	0.279	4.52E-04	2.33E-02	NIK/NF-kappaB signaling
**GO:0090174**	100	0.356	4.63E-04	2.39E-02	organelle membrane fusion
**GO:0016601**	34	0.590	4.69E-04	2.42E-02	Rac protein signal transduction
**GO:0060760**	54	0.489	4.70E-04	2.42E-02	positive regulation of response to cytokine stimulus
**GO:0014743**	59	0.461	4.72E-04	2.43E-02	regulation of muscle hypertrophy
**GO:0051146**	273	0.213	4.78E-04	2.46E-02	striated muscle cell differentiation
**GO:0098581**	17	0.888	4.91E-04	2.52E-02	detection of external biotic stimulus
**GO:2000241**	151	0.284	4.93E-04	2.53E-02	regulation of reproductive process
**GO:1904645**	45	0.539	4.93E-04	2.53E-02	response to amyloid-beta
**GO:1902117**	72	0.418	4.95E-04	2.54E-02	positive regulation of organelle assembly

**GO:0072577**	54	0.470	4.97E-04	2.54E-02	endothelial cell apoptotic process
**GO:0001764**	157	0.278	5.04E-04	2.58E-02	neuron migration
**GO:0062208**	29	0.710	5.06E-04	2.58E-02	positive regulation of pattern recognition receptor signaling pathway
**GO:0003177**	21	0.712	5.07E-04	2.59E-02	pulmonary valve development
**GO:1901890**	97	0.353	5.08E-04	2.59E-02	positive regulation of cell junction assembly
**GO:0035335**	97	0.354	5.10E-04	2.60E-02	peptidyl-tyrosine dephosphorylation
**GO:0030042**	52	0.501	5.13E-04	2.61E-02	actin filament depolymerization
**GO:1903305**	160	0.277	5.15E-04	2.62E-02	regulation of regulated secretory pathway
**GO:0051497**	21	0.798	5.20E-04	2.64E-02	negative regulation of stress fiber assembly
**GO:0038095**	112	0.331	5.26E-04	2.67E-02	Fc-epsilon receptor signaling pathway
**GO:0033622**	22	0.735	5.27E-04	2.67E-02	integrin activation
**GO:0051169**	332	0.192	5.29E-04	2.68E-02	nuclear transport
**GO:0030522**	267	0.214	5.29E-04	2.68E-02	intracellular receptor signaling pathway
**GO:0034764**	196	0.250	5.30E-04	2.68E-02	positive regulation of transmembrane transport
**GO:0032418**	72	0.413	5.30E-04	2.68E-02	lysosome localization
**GO:0051310**	58	0.463	5.29E-04	2.68E-02	metaphase plate congression
**GO:0051984**	28	0.702	5.30E-04	2.68E-02	positive regulation of chromosome segregation
**GO:0043383**	12	0.989	5.29E-04	2.68E-02	negative T cell selection
**GO:0043244**	106	0.341	5.34E-04	2.69E-02	regulation of protein-containing complex disassembly
**GO:0002730**	10	1.073	5.38E-04	2.71E-02	regulation of dendritic cell cytokine production
**GO:1903203**	26	0.732	5.43E-04	2.73E-02	regulation of oxidative stress-induced neuron death
**GO:0030224**	34	0.639	5.44E-04	2.73E-02	monocyte differentiation
**GO:1903131**	34	0.639	5.44E-04	2.73E-02	mononuclear cell differentiation
**GO:0007219**	184	0.256	5.51E-04	2.76E-02	Notch signaling pathway
**GO:0016311**	459	0.163	5.54E-04	2.78E-02	dephosphorylation
**GO:0032722**	36	0.612	5.58E-04	2.79E-02	positive regulation of chemokine production
**GO:0031100**	72	0.416	5.59E-04	2.79E-02	animal organ regeneration
**GO:2000249**	38	0.569	5.59E-04	2.79E-02	regulation of actin cytoskeleton reorganization
**GO:0043374**	14	0.826	5.59E-04	2.79E-02	CD8-positive, alpha-beta T cell differentiation
**GO:0007223**	37	0.561	5.68E-04	2.83E-02	Wnt signaling pathway, calcium modulating pathway
**GO:1904589**	61	0.446	5.74E-04	2.86E-02	regulation of protein import

**GO:1901889**	30	0.621	5.79E-04	2.88E-02	negative regulation of cell junction assembly
**GO:0070741**	40	0.543	5.82E-04	2.90E-02	response to interleukin-6
**GO:0043507**	72	0.406	5.84E-04	2.90E-02	positive regulation of JUN kinase activity
**GO:0032330**	47	0.502	5.97E-04	2.97E-02	regulation of chondrocyte differentiation
**GO:2001185**	14	0.837	6.01E-04	2.98E-02	regulation of CD8-positive, alpha-beta T cell activation
**GO:0002063**	46	0.498	6.04E-04	3.00E-02	chondrocyte development
**GO:0000186**	55	0.572	4.20E-05	9.80E-03	activation of MAPKK activity
**GO:0032733**	25	0.688	6.12E-04	3.03E-02	positive regulation of interleukin-10 production
**GO:0120032**	168	0.266	6.25E-04	3.09E-02	regulation of plasma membrane bounded cell projection assembly
**GO:0033048**	39	0.555	6.27E-04	3.10E-02	negative regulation of mitotic sister chromatid segregation
**GO:0070265**	49	0.502	6.33E-04	3.12E-02	necrotic cell death
**GO:0002544**	19	0.763	6.36E-04	3.14E-02	chronic inflammatory response
**GO:0031116**	27	0.656	6.40E-04	3.16E-02	positive regulation of microtubule polymerization
**GO:0032409**	265	0.212	6.42E-04	3.16E-02	regulation of transporter activity
**GO:0048535**	16	0.870	6.48E-04	3.19E-02	lymph node development
**GO:0045742**	29	0.640	6.55E-04	3.22E-02	positive regulation of epidermal growth factor receptor signaling pathway
**GO:0090025**	22	0.706	6.55E-04	3.22E-02	regulation of monocyte chemotaxis
**GO:0031345**	177	0.259	6.58E-04	3.23E-02	negative regulation of cell projection organization
**GO:0046546**	137	0.293	6.60E-04	3.23E-02	development of primary male sexual characteristics
**GO:0045639**	90	0.366	6.59E-04	3.23E-02	positive regulation of myeloid cell differentiation
**GO:0070570**	30	0.627	6.60E-04	3.23E-02	regulation of neuron projection regeneration
**GO:0035455**	18	0.756	6.60E-04	3.23E-02	response to interferon-alpha
**GO:0030834**	49	0.506	6.63E-04	3.24E-02	regulation of actin filament depolymerization
**GO:0002886**	53	0.470	6.71E-04	3.28E-02	regulation of myeloid leukocyte mediated immunity
**GO:0090630**	82	0.384	6.73E-04	3.29E-02	activation of GTPase activity
**GO:0002467**	15	0.915	6.81E-04	3.32E-02	germinal center formation
**GO:0007612**	146	0.284	6.88E-04	3.35E-02	learning
**GO:0045840**	53	0.470	6.93E-04	3.36E-02	positive regulation of mitotic nuclear division
**GO:0071677**	23	0.706	6.92E-04	3.36E-02	positive regulation of mononuclear cell migration
**GO:0031498**	21	0.765	7.00E-04	3.40E-02	chromatin disassembly

**GO:0006913**	329	0.189	7.06E-04	3.42E-02	nucleocytoplasmic transport
**GO:0010715**	16	0.861	7.08E-04	3.43E-02	regulation of extracellular matrix disassembly
**GO:0030879**	143	0.284	7.11E-04	3.44E-02	mammary gland development
**GO:1902099**	53	0.474	7.19E-04	3.48E-02	regulation of metaphase/anaphase transition of cell cycle
**GO:0010876**	370	0.178	7.24E-04	3.50E-02	lipid localization
**GO:0050765**	21	0.776	7.42E-04	3.58E-02	negative regulation of phagocytosis
**GO:0007623**	208	0.235	7.44E-04	3.59E-02	circadian rhythm
**GO:0043550**	63	0.429	7.52E-04	3.62E-02	regulation of lipid kinase activity
**GO:0090218**	37	0.560	7.52E-04	3.62E-02	positive regulation of lipid kinase activity
**GO:1903053**	38	0.561	7.55E-04	3.63E-02	regulation of extracellular matrix organization
**GO:0033044**	326	0.188	7.60E-04	3.65E-02	regulation of chromosome organization
**GO:0060491**	170	0.260	7.60E-04	3.65E-02	regulation of cell projection assembly
**GO:0090049**	38	0.540	7.64E-04	3.67E-02	regulation of cell migration involved in sprouting angiogenesis
**GO:0008286**	139	0.287	7.74E-04	3.71E-02	insulin receptor signaling pathway
**GO:0045059**	13	0.961	7.76E-04	3.72E-02	positive thymic T cell selection
**GO:0044003**	37	0.542	7.78E-04	3.72E-02	modulation by symbiont of host process
**GO:0048278**	65	0.425	7.80E-04	3.73E-02	vesicle docking
**GO:0030512**	72	0.398	7.84E-04	3.75E-02	negative regulation of transforming growth factor beta receptor signaling pathway
**GO:1903845**	74	0.392	7.93E-04	3.78E-02	negative regulation of cellular response to transforming growth factor beta stimulus
**GO:2000377**	175	0.256	7.94E-04	3.78E-02	regulation of reactive oxygen species metabolic process
**GO:0048678**	73	0.394	7.94E-04	3.78E-02	response to axon injury
**GO:0060142**	28	0.641	7.96E-04	3.79E-02	regulation of syncytium formation by plasma membrane fusion
**GO:0070669**	14	0.862	7.97E-04	3.79E-02	response to interleukin-2
**GO:0007492**	77	0.381	8.17E-04	3.88E-02	endoderm development
**GO:0034162**	25	0.748	8.17E-04	3.88E-02	toll-like receptor 9 signaling pathway
**GO:0050798**	38	0.552	8.20E-04	3.89E-02	activated T cell proliferation
**GO:0010977**	148	0.278	8.23E-04	3.90E-02	negative regulation of neuron projection development
**GO:0030033**	16	0.863	8.35E-04	3.95E-02	microvillus assembly

**GO:0043551**	54	0.453	8.44E-04	3.99E-02	regulation of phosphatidylinositol 3-kinase activity
**GO:0001782**	28	0.665	8.55E-04	4.04E-02	B cell homeostasis
**GO:0016241**	167	0.260	8.56E-04	4.04E-02	regulation of macroautophagy
**GO:0034113**	57	0.451	8.64E-04	4.07E-02	heterotypic cell-cell adhesion
**GO:0043297**	61	0.426	8.71E-04	4.10E-02	apical junction assembly
**GO:0006907**	21	0.728	8.71E-04	4.10E-02	pinocytosis
**GO:0034724**	41	0.531	8.74E-04	4.11E-02	DNA replication-independent nucleosome organization
**GO:0010878**	16	0.784	8.97E-04	4.21E-02	cholesterol storage
**GO:0045841**	36	0.561	9.00E-04	4.22E-02	negative regulation of mitotic metaphase/anaphase transition
**GO:2000816**	36	0.561	9.00E-04	4.22E-02	negative regulation of mitotic sister chromatid separation
**GO:0072583**	41	0.513	9.10E-04	4.26E-02	clathrin-dependent endocytosis
**GO:0008584**	136	0.286	9.12E-04	4.27E-02	male gonad development
**GO:0060047**	270	0.203	9.25E-04	4.33E-02	heart contraction
**GO:0045741**	10	1.054	9.31E-04	4.35E-02	positive regulation of epidermal growth factor-activated receptor activity
**GO:0062033**	17	0.779	9.32E-04	4.35E-02	positive regulation of mitotic sister chromatid segregation
**GO:0098974**	10	0.958	9.35E-04	4.37E-02	postsynaptic actin cytoskeleton organization
**GO:0035723**	13	0.823	9.44E-04	4.40E-02	interleukin-15-mediated signaling pathway
**GO:0071350**	13	0.823	9.44E-04	4.40E-02	cellular response to interleukin-15
**GO:0043524**	147	0.273	9.48E-04	4.41E-02	negative regulation of neuron apoptotic process
**GO:0009991**	482	0.152	9.59E-04	4.46E-02	response to extracellular stimulus
**GO:1902100**	37	0.549	9.63E-04	4.47E-02	negative regulation of metaphase/anaphase transition of cell cycle
**GO:1905819**	37	0.549	9.63E-04	4.47E-02	negative regulation of chromosome separation
**GO:0043931**	19	0.707	9.72E-04	4.51E-02	ossification involved in bone maturation
**GO:0030307**	158	0.265	9.73E-04	4.51E-02	positive regulation of cell growth
**GO:0030032**	62	0.424	9.83E-04	4.55E-02	lamellipodium assembly
**GO:0051058**	56	0.448	9.84E-04	4.55E-02	negative regulation of small GTPase mediated signal transduction
**GO:0006904**	44	0.511	9.84E-04	4.55E-02	vesicle docking involved in exocytosis
**GO:0034111**	15	0.805	9.84E-04	4.55E-02	negative regulation of homotypic cell-cell adhesion

**GO:0034123**	21	0.833	9.84E-04	4.55E-02	positive regulation of toll-like receptor signaling pathway
**GO:0097061**	76	0.381	9.92E-04	4.58E-02	dendritic spine organization
**GO:0043523**	208	0.230	1.01E-03	4.64E-02	regulation of neuron apoptotic process
**GO:0035051**	146	0.274	1.01E-03	4.64E-02	cardiocyte differentiation
**GO:0010950**	153	0.268	1.01E-03	4.67E-02	positive regulation of endopeptidase activity
**GO:0043044**	74	0.385	1.03E-03	4.72E-02	ATP-dependent chromatin remodeling
**GO:0008217**	174	0.250	1.03E-03	4.72E-02	regulation of blood pressure
**GO:1902751**	30	0.646	1.04E-03	4.75E-02	positive regulation of cell cycle G2/M phase transition
**GO:0008306**	79	0.372	1.04E-03	4.77E-02	associative learning
**GO:0017157**	215	0.226	1.04E-03	4.78E-02	regulation of exocytosis
**GO:1903725**	84	0.359	1.04E-03	4.78E-02	regulation of phospholipid metabolic process
**GO:0061614**	45	0.488	1.05E-03	4.81E-02	pri-miRNA transcription by RNA polymerase II
**GO:0070232**	31	0.620	1.05E-03	4.81E-02	regulation of T cell apoptotic process
**GO:0007260**	75	0.380	1.06E-03	4.83E-02	tyrosine phosphorylation of STAT protein
**GO:0034142**	34	0.592	1.06E-03	4.85E-02	toll-like receptor 4 signaling pathway
**GO:0002709**	59	0.432	1.07E-03	4.86E-02	regulation of T cell mediated immunity
**GO:0060998**	70	0.391	1.07E-03	4.87E-02	regulation of dendritic spine development
**GO:0001569**	31	0.573	1.07E-03	4.87E-02	branching involved in blood vessel morphogenesis
**GO:0046661**	158	0.261	1.08E-03	4.90E-02	male sex differentiation
**GO:0001975**	34	0.559	1.09E-03	4.96E-02	response to amphetamine
**GO:0010743**	27	0.618	1.10E-03	4.98E-02	regulation of macrophage derived foam cell differentiation
**Molecular functions in Genetic group 1 in the most efficient group.**					
**Go term**	**Number of genes**	**LOR***	**pvalue**	**padj**	**Molecular functions**
**GO:0036312**	10	1.108	1.45E-06	2.38E-03	phosphatidylinositol 3-kinase regulatory subunit binding
**GO:0023026**	10	1.089	6.87E-06	7.50E-03	MHC class II protein complex binding
**GO:0016303**	10	1.038	1.79E-04	1.61E-02	1-phosphatidylinositol-3-kinase activity
**GO:0048407**	11	1.301	4.05E-10	1.74E-07	platelet-derived growth factor binding
**GO:0035004**	11	1.035	5.37E-06	7.95E-03	phosphatidylinositol 3-kinase activity

**GO:0097493**	11	1.035	8.94E-06	9.28E-03	structural molecule activity conferring elasticity
**GO:0004115**	11	0.967	2.55E-04	2.13E-02	3',5'-cyclic-AMP phosphodiesterase activity
**GO:0005522**	11	0.981	7.18E-04	4.99E-02	profilin binding
**GO:0031005**	12	0.943	4.99E-04	3.81E-02	filamin binding
**GO:0042834**	13	1.026	3.53E-04	2.82E-02	peptidoglycan binding
**GO:0017160**	15	0.969	2.45E-04	2.07E-02	Ral GTPase binding
**GO:0023023**	16	1.011	2.99E-07	5.99E-04	MHC protein complex binding
**GO:0035173**	17	0.916	5.81E-06	6.53E-03	histone kinase activity
**GO:0008139**	20	0.802	2.09E-04	1.82E-02	nuclear localization sequence binding
**GO:0050431**	22	0.900	1.01E-04	1.03E-02	transforming growth factor beta binding
**GO:0043325**	25	0.697	3.80E-04	3.01E-02	phosphatidylinositol-3,4-bisphosphate binding
**GO:0042974**	26	0.874	4.02E-07	7.54E-04	retinoic acid receptor binding
**GO:0042287**	27	0.757	4.49E-06	5.69E-03	MHC protein binding
**GO:0043236**	27	0.671	4.62E-04	3.59E-02	laminin binding
**GO:0071889**	28	0.711	1.09E-04	1.08E-02	14-3-3 protein binding
**GO:0003823**	29	0.710	6.31E-04	4.57E-02	antigen binding
**GO:0043548**	31	0.828	1.26E-07	2.93E-04	phosphatidylinositol 3-kinase binding
**GO:0043394**	34	0.644	1.51E-04	1.37E-02	proteoglycan binding
**GO:0042169**	36	0.635	7.06E-06	7.61E-03	SH2 domain binding
**GO:0005547**	37	0.790	1.44E-07	3.18E-04	phosphatidylinositol-3,4,5-trisphosphate binding
**GO:0030020**	40	0.641	2.87E-06	4.25E-03	extracellular matrix structural constituent conferring tensile strength
**GO:0001102**	41	0.573	2.75E-04	2.26E-02	RNA polymerase II activating transcription factor binding
**GO:0004715**	46	0.896	1.75E-12	1.26E-09	non-membrane spanning protein tyrosine kinase activity
**GO:0042379**	48	0.496	6.55E-04	4.70E-02	chemokine receptor binding
**GO:0045309**	49	0.555	1.31E-04	1.25E-02	protein phosphorylated amino acid binding
**GO:0050840**	53	0.546	5.49E-06	6.53E-03	extracellular matrix binding
**GO:0004714**	62	0.496	1.14E-04	1.12E-02	transmembrane receptor protein tyrosine kinase activity
**GO:0005518**	64	0.681	6.84E-09	2.11E-06	collagen binding
**GO:0048365**	66	0.620	8.51E-08	2.10E-04	Rac GTPase binding
**GO:0001618**	68	0.490	5.25E-06	6.47E-03	virus receptor activity
**GO:0030971**	68	0.479	1.24E-04	1.20E-02	receptor tyrosine kinase binding
**GO:0140272**	69	0.482	6.07E-06	6.70E-03	exogenous protein binding
**GO:0003725**	72	0.505	3.28E-06	4.42E-03	double-stranded RNA binding
**GO:0033613**	74	0.511	1.39E-06	2.38E-03	activating transcription factor binding

**GO:0038024**	75	0.400	5.97E-04	4.38E-02	cargo receptor activity
**GO:0005089**	77	0.434	1.50E-04	1.37E-02	Rho guanyl-nucleotide exchange factor activity
**GO:0019199**	80	0.474	3.06E-06	4.25E-03	transmembrane receptor protein kinase activity
**GO:0046332**	80	0.473	3.02E-06	4.25E-03	SMAD binding
**GO:0003777**	80	0.459	5.72E-06	6.53E-03	microtubule motor activity
**GO:1990782**	90	0.524	1.34E-07	3.05E-04	protein tyrosine kinase binding
**GO:0035258**	91	0.404	1.46E-04	1.35E-02	steroid hormone receptor binding
**GO:0004896**	94	0.424	4.64E-06	5.79E-03	cytokine receptor activity
**GO:0004725**	96	0.359	4.66E-04	3.59E-02	protein tyrosine phosphatase activity
**GO:1902936**	101	0.389	1.04E-04	1.06E-02	phosphatidylinositol bisphosphate binding
**GO:0140375**	109	0.447	3.63E-07	6.98E-04	immune receptor activity
**GO:0016922**	110	0.402	2.98E-06	4.25E-03	nuclear receptor binding
**GO:0019955**	122	0.534	9.62E-10	3.73E-07	cytokine binding
**GO:0044325**	125	0.308	5.97E-04	4.38E-02	ion channel binding
**GO:0005178**	126	0.700	2.22E-01	4.79E-13	integrin binding
**GO:0017124**	127	0.552	1.26E-10	6.01E-08	SH3 domain binding
**GO:0019838**	129	0.384	1.70E-06	2.72E-03	growth factor binding
**GO:0003774**	132	0.448	4.25E-08	1.08E-04	motor activity
**GO:0005088**	134	0.419	1.70E-07	3.66E-04	Ras guanyl-nucleotide exchange factor activity
**GO:0004713**	135	0.462	9.36E-09	2.78E-06	protein tyrosine kinase activity
**GO:0019903**	135	0.350	5.57E-06	6.53E-03	protein phosphatase binding
**GO:0070851**	136	0.434	4.02E-08	1.05E-04	growth factor receptor binding
**GO:1901981**	152	0.432	1.55E-08	4.45E-06	phosphatidylinositol phosphate binding
**GO:0035257**	152	0.352	1.83E-06	2.86E-03	nuclear hormone receptor binding
**GO:0005201**	157	0.551	9.50E-13	9.10E-10	extracellular matrix structural constituent
**GO:0017048**	171	0.488	2.39E-11	1.29E-08	Rho GTPase binding
**GO:0004721**	176	0.280	2.33E-04	2.01E-02	phosphoprotein phosphatase activity
**GO:0019902**	180	0.307	4.43E-06	5.69E-03	phosphatase binding
**GO:0051427**	182	0.355	2.23E-07	4.69E-04	hormone receptor binding
**GO:0051015**	190	0.437	3.90E-10	1.74E-07	actin filament binding
**GO:0005516**	195	0.408	1.35E-09	4.30E-07	calmodulin binding
**GO:0005539**	199	0.336	2.59E-07	5.32E-04	glycosaminoglycan binding
**GO:0005085**	210	0.304	1.46E-06	2.38E-03	guanyl-nucleotide exchange factor activity
**GO:0035091**	234	0.450	1.38E-12	1.11E-09	phosphatidylinositol binding
**GO:0008017**	236	0.324	9.05E-08	2.17E-04	microtubule binding
**GO:0030246**	237	0.250	1.36E-04	1.27E-02	carbohydrate binding

**GO:0005096**	242	0.511	1.00E-15	1.44E-12	GTPase activator activity
**GO:0005126**	249	0.331	2.25E-08	6.26E-06	cytokine receptor binding
**GO:0001664**	258	0.263	2.90E-06	4.25E-03	G protein-coupled receptor binding
**GO:0061629**	265	0.230	2.08E-04	1.82E-02	RNA polymerase II-specific DNA-binding transcription factor binding
**GO:0030695**	272	0.485	5.53E-02	9.53E-13	GTPase regulator activity
**GO:0060589**	311	0.372	1.34E-12	8.25E-09	nucleoside-triphosphatase regulator activity
**GO:0045296**	313	0.496	8.69E-04	4.05E-02	cadherin binding
**GO:0015631**	321	0.210	2.04E-04	1.82E-02	tubulin binding
**GO:0140297**	336	0.223	5.59E-06	6.53E-03	DNA-binding transcription factor binding
**GO:0042578**	355	0.188	4.48E-04	3.51E-02	phosphoric ester hydrolase activity
**GO:0016887**	402	0.175	5.36E-04	4.05E-02	ATPase activity
**GO:0017016**	403	0.366	6.00E-14	6.46E-11	Ras GTPase binding
**GO:0005543**	408	0.405	1.54E-12	4.44E-13	phospholipid binding
**GO:0003779**	414	0.433	9.39E-04	4.05E-02	actin binding
**GO:0031267**	417	0.367	2.17E-14	2.67E-11	small GTPase binding
**GO:0001216**	420	0.206	2.97E-06	4.25E-03	DNA-binding transcription activator activity
**GO:0001228**	420	0.206	2.97E-06	4.25E-03	DNA-binding transcription activator activity, RNA polymerase II-specific
**GO:0004674**	430	0.300	1.02E-10	5.15E-08	protein serine/threonine kinase activity
**GO:0046982**	451	0.247	2.40E-08	6.45E-06	protein heterodimerization activity
**GO:0008047**	476	0.317	1.42E-12	1.11E-09	enzyme activator activity
**GO:0016831**	34	-0.594	7.15E-04	4.99E-02	carboxy-lyase activity
**GO:0034062**	41	-0.538	7.03E-04	4.97E-02	5'-3' RNA polymerase activity
**GO:0097747**	41	-0.538	7.03E-04	4.97E-02	RNA polymerase activity
**GO:0051536**	61	-0.445	6.00E-04	4.38E-02	iron-sulfur cluster binding
**GO:0051540**	61	-0.445	6.00E-04	4.38E-02	metal cluster binding
**GO:0000030**	25	-0.809	3.45E-04	2.78E-02	mannosyltransferase activity
**GO:0004519**	116	-0.339	2.90E-04	2.36E-02	endonuclease activity
**GO:0009982**	13	-0.951	2.71E-04	2.25E-02	pseudouridine synthase activity
**GO:0008175**	33	-0.615	2.38E-04	2.03E-02	tRNA methyltransferase activity
**GO:0008320**	19	-0.809	1.33E-04	1.26E-02	protein transmembrane transporter activity
**GO:0016783**	11	-1.041	1.07E-04	1.07E-02	sulfurtransferase activity
**GO:0004549**	16	-1.059	7.56E-06	7.95E-03	tRNA-specific ribonuclease activity
**GO:0008173**	63	-0.503	5.73E-06	6.53E-03	RNA methyltransferase activity
**GO:0016829**	183	-0.300	5.83E-06	6.53E-03	lyase activity

**GO:0140318**	21	-0.819	4.29E-06	5.61E-03	protein transporter activity
**GO:0009055**	101	-0.412	3.99E-06	5.30E-03	electron transfer activity
**GO:0016779**	116	-0.393	3.28E-06	4.42E-03	nucleotidyltransferase activity
**GO:0016651**	95	-0.449	1.42E-06	2.38E-03	oxidoreductase activity, acting on NAD(P)H
**GO:0008168**	202	-0.312	1.09E-06	1.92E-03	methyltransferase activity
**GO:0016741**	212	-0.304	1.09E-06	1.92E-03	transferase activity, transferring one-carbon groups
**GO:0004518**	190	-0.323	1.01E-06	1.85E-03	nuclease activity
**GO:0015651**	10	-1.182	3.64E-07	6.98E-04	quaternary ammonium group transmembrane transporter activity
**GO:0003735**	155	-0.453	1.15E-09	3.80E-07	structural constituent of ribosome
**GO:0003954**	38	-0.959	1.08E-09	3.73E-07	NADH dehydrogenase activity
**GO:0008137**	38	-0.959	1.08E-09	3.73E-07	NADH dehydrogenase (ubiquinone) activity
**GO:0050136**	38	-0.959	1.08E-09	3.73E-07	NADH dehydrogenase (quinone) activity
**GO:0016655**	50	-0.805	7.20E-10	2.96E-07	oxidoreductase activity, acting on NAD(P)H, quinone or similar compound as acceptor
**GO:0140098**	300	-0.374	1.78E-11	1.02E-08	catalytic activity, acting on RNA
**GO:0140101**	119	-0.599	5.86E-12	3.89E-09	catalytic activity, acting on a tRNA
**Cellular components in Genetic group 1 in the most efficient group.**					
**Go term**	**Number of genes**	**LOR***	**pvalue**	**padj**	**Cellular components**
**GO:0005743**	435	-0.793	1.90E-57	1.03E-53	mitochondrial inner membrane
**GO:0098798**	239	-0.958	5.51E-52	1.50E-48	mitochondrial protein complex
**GO:0019866**	492	-0.677	7.03E-46	1.27E-42	organelle inner membrane
**GO:0005759**	456	-0.675	1.01E-42	1.37E-39	mitochondrial matrix
**GO:0000313**	86	-1.131	1.52E-35	1.38E-32	organellar ribosome
**GO:0005761**	86	-1.131	1.52E-35	1.38E-32	mitochondrial ribosome
**GO:0005925**	400	0.629	1.48E-34	1.15E-31	focal adhesion
**GO:0030055**	407	0.619	5.30E-34	3.60E-31	cell-substrate junction
**GO:0000315**	56	-1.172	7.76E-28	4.21E-25	organellar large ribosomal subunit
**GO:0005762**	56	-1.172	7.76E-28	4.21E-25	mitochondrial large ribosomal subunit
**GO:0098552**	466	0.511	3.28E-26	1.62E-23	side of membrane
**GO:0098800**	116	-0.957	1.36E-25	6.17E-23	inner mitochondrial membrane protein complex
**GO:0009897**	283	0.565	3.58E-20	1.50E-17	external side of plasma membrane
**GO:0031252**	398	0.473	4.79E-20	1.86E-17	cell leading edge
**GO:0098589**	322	0.504	8.65E-19	3.13E-16	membrane region

**GO:0098857**	310	0.507	2.23E-18	7.57E-16	membrane microdomain
**GO:0045121**	309	0.507	2.84E-18	9.07E-16	membrane raft
**GO:0015629**	487	0.397	1.53E-17	4.62E-15	actin cytoskeleton
**GO:0030667**	277	0.512	6.32E-17	1.81E-14	secretory granule membrane
**GO:0070469**	81	-0.916	8.03E-17	2.18E-14	respirasome
**GO:0098803**	69	-0.957	1.60E-15	4.14E-13	respiratory chain complex
**GO:0030027**	190	0.561	3.24E-14	8.00E-12	lamellipodium
**GO:0005746**	74	-0.871	3.57E-14	8.42E-12	mitochondrial respirasome
**GO:0062023**	375	0.397	4.58E-14	1.04E-11	collagen-containing extracellular matrix
**GO:0031012**	488	0.344	1.06E-13	2.30E-11	extracellular matrix
**GO:0044391**	182	-0.533	3.56E-13	7.44E-11	ribosomal subunit
**GO:0001726**	168	0.570	7.80E-13	1.57E-10	ruffle
**GO:0005840**	229	-0.467	1.65E-12	3.21E-10	ribosome
**GO:0030139**	277	0.412	1.75E-11	3.28E-09	endocytic vesicle
**GO:0015934**	115	-0.607	3.02E-11	5.47E-09	large ribosomal subunit
**GO:0043235**	382	0.338	8.71E-11	1.53E-08	receptor complex
**GO:0000775**	190	0.479	1.41E-10	2.39E-08	chromosome, centromeric region
**GO:0005769**	331	0.357	1.85E-10	3.04E-08	early endosome
**GO:0098858**	197	0.461	2.29E-10	3.66E-08	actin-based cell projection
**GO:0005911**	417	0.315	2.53E-10	3.67E-08	cell-cell junction
**GO:0005747**	43	-1.007	2.57E-10	3.67E-08	mitochondrial respiratory chain complex I
**GO:0030964**	43	-1.007	2.57E-10	3.67E-08	NADH dehydrogenase complex
**GO:0045271**	43	-1.007	2.57E-10	3.67E-08	respiratory chain complex I
**GO:0019897**	162	0.497	3.69E-10	5.04E-08	extrinsic component of plasma membrane
**GO:0045335**	123	0.589	3.71E-10	5.04E-08	phagocytic vesicle
**GO:0005938**	304	0.363	5.59E-10	7.41E-08	cell cortex
**GO:0030175**	98	0.639	9.01E-10	1.17E-07	filopodium
**GO:0070820**	151	0.498	1.17E-09	1.47E-07	tertiary granule
**GO:0005884**	105	0.604	1.84E-09	2.27E-07	actin filament
**GO:0032432**	73	0.695	2.08E-09	2.52E-07	actin filament bundle
**GO:0042641**	77	0.675	2.78E-09	3.29E-07	actomyosin
**GO:0005819**	338	0.327	4.20E-09	4.85E-07	spindle
**GO:0000314**	28	-0.973	5.31E-09	5.89E-07	organellar small ribosomal subunit
**GO:0005763**	28	-0.973	5.31E-09	5.89E-07	mitochondrial small ribosomal subunit
**GO:0009898**	151	0.478	1.10E-08	1.19E-06	cytoplasmic side of plasma membrane
**GO:0001725**	67	0.689	1.48E-08	1.55E-06	stress fiber
**GO:0097517**	67	0.689	1.48E-08	1.55E-06	contractile actin filament bundle
**GO:0010008**	443	0.273	1.65E-08	1.69E-06	endosome membrane
**GO:0001772**	37	1.049	2.15E-08	2.14E-06	immunological synapse

**GO:0031304**	40	-0.838	2.21E-08	2.14E-06	intrinsic component of mitochondrial inner membrane
**GO:0031305**	40	-0.838	2.21E-08	2.14E-06	integral component of mitochondrial inner membrane
**GO:0098687**	322	0.316	2.53E-08	2.41E-06	chromosomal region
**GO:0035579**	86	0.617	2.60E-08	2.43E-06	specific granule membrane
**GO:0042581**	151	0.463	3.09E-08	2.85E-06	specific granule
**GO:0043202**	92	0.598	3.94E-08	3.57E-06	lysosomal lumen
**GO:0031901**	133	0.476	5.28E-08	4.70E-06	early endosome membrane
**GO:0005874**	399	0.276	5.97E-08	5.23E-06	microtubule
**GO:0042629**	22	1.077	7.13E-08	6.15E-06	mast cell granule
**GO:0070821**	66	0.651	7.49E-08	6.36E-06	tertiary granule membrane
**GO:0045177**	375	0.280	8.96E-08	7.48E-06	apical part of cell
**GO:0005902**	79	0.616	9.95E-08	8.19E-06	microvillus
**GO:0005775**	158	0.435	1.02E-07	8.29E-06	vacuolar lumen
**GO:0002102**	30	1.053	1.07E-07	8.58E-06	podosome
**GO:0031234**	88	0.571	1.37E-07	1.08E-05	extrinsic component of cytoplasmic side of plasma membrane
**GO:0098562**	177	0.403	1.53E-07	1.17E-05	cytoplasmic side of membrane
**GO:0150034**	285	0.314	1.53E-07	1.17E-05	distal axon
**GO:0034774**	303	0.305	1.87E-07	1.41E-05	secretory granule lumen
**GO:0030427**	174	0.398	1.97E-07	1.47E-05	site of polarized growth
**GO:0016323**	212	0.360	2.19E-07	1.61E-05	basolateral plasma membrane
**GO:0030426**	171	0.398	2.66E-07	1.93E-05	growth cone
**GO:0042470**	104	0.515	2.78E-07	1.96E-05	melanosome
**GO:0048770**	104	0.515	2.78E-07	1.96E-05	pigment granule
**GO:0005581**	93	0.528	2.92E-07	2.03E-05	collagen trimer
**GO:0031983**	309	0.297	2.95E-07	2.03E-05	vesicle lumen
**GO:0000776**	132	0.454	3.12E-07	2.12E-05	kinetochore
**GO:0060205**	307	0.296	3.44E-07	2.31E-05	cytoplasmic vesicle lumen
**GO:0098978**	358	0.268	5.40E-07	3.58E-05	glutamatergic synapse
**GO:0032592**	68	-0.596	7.15E-07	4.68E-05	integral component of mitochondrial membrane
**GO:0098573**	69	-0.584	1.00E-06	6.47E-05	intrinsic component of mitochondrial membrane
**GO:0072686**	106	0.493	1.13E-06	7.20E-05	mitotic spindle
**GO:0005871**	52	0.697	1.20E-06	7.61E-05	kinesin complex
**GO:0005604**	92	0.498	1.26E-06	7.66E-05	basement membrane
**GO:0005766**	138	0.422	1.24E-06	7.66E-05	primary lysosome
**GO:0042582**	138	0.422	1.24E-06	7.66E-05	azurophil granule
**GO:0031253**	313	0.278	1.30E-06	7.86E-05	cell projection membrane
**GO:0030136**	173	0.370	1.38E-06	8.24E-05	clathrin-coated vesicle
**GO:0005875**	148	0.402	1.42E-06	8.41E-05	microtubule associated complex
**GO:0098852**	329	0.268	1.69E-06	9.77E-05	lytic vacuole membrane
**GO:1905368**	92	-0.505	1.69E-06	9.77E-05	peptidase complex

**GO:0005765**	328	0.267	1.83E-06	1.04E-04	lysosomal membrane
**GO:0033643**	15	1.053	2.54E-06	1.44E-04	host cell part
**GO:0000779**	116	0.443	2.81E-06	1.57E-04	condensed chromosome, centromeric region
**GO:1990204**	101	-0.471	2.83E-06	1.57E-04	oxidoreductase complex
**GO:0030496**	170	0.366	2.87E-06	1.57E-04	midbody
**GO:0005758**	76	-0.552	2.88E-06	1.57E-04	mitochondrial intermembrane space
**GO:0019898**	288	0.278	3.13E-06	1.69E-04	extrinsic component of membrane
**GO:0098685**	85	0.504	3.39E-06	1.81E-04	Schaffer collateral - CA1 synapse
**GO:0030684**	73	-0.532	3.46E-06	1.83E-04	preribosome
**GO:0000777**	103	0.466	3.54E-06	1.85E-04	condensed chromosome kinetochore
**GO:0030666**	145	0.389	3.86E-06	2.00E-04	endocytic vesicle membrane
**GO:0005774**	381	0.239	4.12E-06	2.11E-04	vacuolar membrane
**GO:0016461**	10	1.157	5.65E-06	2.87E-04	unconventional myosin complex
**GO:0016324**	310	0.260	6.16E-06	3.10E-04	apical plasma membrane
**GO:0098644**	19	0.956	8.71E-06	4.34E-04	complex of collagen trimers
**GO:0031256**	166	0.349	9.36E-06	4.62E-04	leading edge membrane
**GO:0044853**	108	0.424	1.22E-05	5.96E-04	plasma membrane raft
**GO:0001891**	26	0.860	1.32E-05	6.40E-04	phagocytic cup
**GO:0098636**	34	0.733	1.39E-05	6.66E-04	protein complex involved in cell adhesion
**GO:0030687**	23	-0.846	1.78E-05	8.48E-04	preribosome, large subunit precursor
**GO:0033646**	13	1.048	1.83E-05	8.58E-04	host intracellular part
**GO:0043656**	13	1.048	1.83E-05	8.58E-04	host intracellular region
**GO:0120114**	74	-0.498	1.85E-05	8.61E-04	Sm-like protein family complex
**GO:0030863**	113	0.410	1.92E-05	8.82E-04	cortical cytoskeleton
**GO:0005905**	68	0.514	2.37E-05	1.08E-03	clathrin-coated pit
**GO:0097386**	26	0.828	2.39E-05	1.08E-03	glial cell projection
**GO:0005876**	57	0.604	2.71E-05	1.22E-03	spindle microtubule
**GO:0034719**	19	-0.886	2.87E-05	1.28E-03	SMN-Sm protein complex
**GO:0034399**	133	0.363	3.33E-05	1.47E-03	nuclear periphery
**GO:0101003**	53	0.556	3.69E-05	1.60E-03	ficolin-1-rich granule membrane
**GO:0031970**	84	-0.459	3.69E-05	1.60E-03	organelle envelope lumen
**GO:0005732**	22	-0.804	3.91E-05	1.69E-03	small nucleolar ribonucleoprotein complex
**GO:0016363**	105	0.404	4.10E-05	1.75E-03	nuclear matrix
**GO:0000793**	211	0.284	4.53E-05	1.92E-03	condensed chromosome
**GO:0032587**	90	0.441	4.81E-05	2.01E-03	ruffle membrane
**GO:0070069**	25	-0.777	4.79E-05	2.01E-03	cytochrome complex
**GO:0008305**	31	0.702	6.59E-05	2.73E-03	integrin complex
**GO:0031314**	14	-0.935	7.42E-05	3.05E-03	extrinsic component of mitochondrial inner membrane
**GO:0032154**	53	0.556	7.76E-05	3.17E-03	cleavage furrow

**GO:0101002**	120	0.364	8.41E-05	3.38E-03	ficolin-1-rich granule
**GO:1904813**	120	0.364	8.41E-05	3.38E-03	ficolin-1-rich granule lumen
**GO:0005753**	18	-0.844	8.74E-05	3.46E-03	mitochondrial proton-transporting ATP synthase complex
**GO:0045259**	18	-0.844	8.74E-05	3.46E-03	proton-transporting ATP synthase complex
**GO:0044309**	160	0.307	1.12E-04	4.40E-03	neuron spine
**GO:0051233**	33	0.757	1.17E-04	4.58E-03	spindle midzone
**GO:0032797**	11	-1.125	1.22E-04	4.71E-03	SMN complex
**GO:1990023**	12	1.015	1.24E-04	4.79E-03	mitotic spindle midzone
**GO:0000790**	342	0.208	1.39E-04	5.31E-03	nuclear chromatin
**GO:0005838**	22	-0.762	1.75E-04	6.64E-03	proteasome regulatory particle
**GO:0030665**	102	0.370	1.84E-04	6.91E-03	clathrin-coated vesicle membrane
**GO:0043197**	158	0.299	1.84E-04	6.91E-03	dendritic spine
**GO:1990752**	29	0.873	1.94E-04	7.23E-03	microtubule end
**GO:0042734**	162	0.293	2.09E-04	7.73E-03	presynaptic membrane
**GO:0045178**	50	0.537	2.26E-04	8.30E-03	basal part of cell
**GO:0005744**	13	-1.072	2.38E-04	8.67E-03	TIM23 mitochondrial import inner membrane translocase complex
**GO:0017053**	76	0.421	2.86E-04	1.04E-02	transcription repressor complex
**GO:0030864**	84	0.404	2.93E-04	1.05E-02	cortical actin cytoskeleton
**GO:0030914**	12	-0.973	3.07E-04	1.10E-02	STAGA complex
**GO:0018995**	66	0.448	3.45E-04	1.22E-02	host cellular component
**GO:0043657**	66	0.448	3.45E-04	1.22E-02	host cell
**GO:0097525**	56	-0.470	3.54E-04	1.24E-02	spliceosomal snRNP complex
**GO:0005583**	11	1.037	4.07E-04	1.41E-02	fibrillar collagen trimer
**GO:0098643**	11	1.037	4.07E-04	1.41E-02	banded collagen fibril
**GO:0099056**	73	0.408	4.61E-04	1.58E-02	integral component of presynaptic membrane
**GO:1902911**	104	0.348	4.63E-04	1.58E-02	protein kinase complex
**GO:0030479**	16	0.904	4.69E-04	1.58E-02	actin cortical patch
**GO:0061645**	16	0.904	4.69E-04	1.58E-02	endocytic patch
**GO:0043292**	233	0.230	5.01E-04	1.68E-02	contractile fiber
**GO:0005770**	239	0.227	5.27E-04	1.76E-02	late endosome
**GO:0035578**	80	0.393	5.92E-04	1.94E-02	azurophil granule lumen
**GO:0071437**	16	0.943	5.95E-04	1.94E-02	invadopodium
**GO:0005665**	13	-0.884	5.92E-04	1.94E-02	RNA polymerase II, core complex
**GO:0016591**	76	-0.394	5.96E-04	1.94E-02	RNA polymerase II, holoenzyme
**GO:0005750**	10	-0.947	6.20E-04	1.98E-02	mitochondrial respiratory chain complex III
**GO:0015935**	70	-0.405	6.14E-04	1.98E-02	small ribosomal subunit
**GO:0045275**	10	-0.947	6.20E-04	1.98E-02	respiratory chain complex III
**GO:0031091**	90	0.362	6.28E-04	1.99E-02	platelet alpha granule

**GO:0005813**	500	0.155	6.48E-04	2.05E-02	centrosome
**GO:0098799**	16	-0.798	6.57E-04	2.06E-02	outer mitochondrial membrane protein complex
**GO:0097504**	11	-1.055	6.72E-04	2.10E-02	Gemini of coiled bodies
**GO:0005788**	299	0.197	7.37E-04	2.28E-02	endoplasmic reticulum lumen
**GO:0042101**	17	0.949	7.38E-04	2.28E-02	T cell receptor complex
**GO:1902554**	84	0.370	8.27E-04	2.54E-02	serine/threonine protein kinase complex
**GO:0042383**	135	0.289	8.47E-04	2.58E-02	sarcolemma
**GO:0030532**	62	-0.421	9.06E-04	2.75E-02	small nuclear ribonucleoprotein complex
**GO:0098802**	181	0.248	9.48E-04	2.86E-02	plasma membrane signaling receptor complex
**GO:0001931**	12	0.978	9.88E-04	2.95E-02	uropod
**GO:0031254**	12	0.978	9.88E-04	2.95E-02	cell trailing edge
**GO:0009925**	34	0.581	1.00E-03	2.97E-02	basal plasma membrane
**GO:0097449**	14	0.929	1.02E-03	3.00E-02	astrocyte projection
**GO:0031527**	17	0.741	1.07E-03	3.13E-02	filopodium membrane
**GO:0032153**	66	0.402	1.20E-03	3.49E-02	cell division site
**GO:0098984**	349	0.175	1.20E-03	3.49E-02	neuron to neuron synapse
**GO:0036019**	19	0.757	1.24E-03	3.57E-02	endolysosome
**GO:0097060**	429	0.157	1.30E-03	3.74E-02	synaptic membrane
**GO:0000307**	42	0.519	1.33E-03	3.81E-02	cyclin-dependent protein kinase holoenzyme complex
**GO:0098793**	491	0.146	1.35E-03	3.85E-02	presynapse
**GO:0035577**	53	0.439	1.40E-03	3.95E-02	azurophil granule membrane
**GO:0032009**	12	0.848	1.48E-03	4.17E-02	early phagosome
**GO:0030016**	222	0.214	1.51E-03	4.23E-02	myofibril
**GO:0055029**	99	-0.320	1.55E-03	4.33E-02	nuclear DNA-directed RNA polymerase complex
**GO:0044295**	28	0.614	1.60E-03	4.43E-02	axonal growth cone
**GO:0030670**	67	0.394	1.65E-03	4.56E-02	phagocytic vesicle membrane
**GO:0030904**	20	0.719	1.66E-03	4.56E-02	retromer complex
**GO:0045277**	14	-0.814	1.76E-03	4.80E-02	respiratory chain complex IV
**GO:0016327**	19	0.665	1.83E-03	4.97E-02	apicolateral plasma membrane
**The significant biological processes in Genetic group 2 in the most efficient group.**					
**Go term**	**Number of genes**	**LOR***	**pvalue**	**padj**	**Biological process**
**GO:0045047**	107	1.016	1.55E-18	7.43E-14	protein targeting to ER
**GO:0006613**	98	1.103	2.57E-18	7.43E-14	cotranslational protein targeting to membrane
**GO:0006614**	94	1.139	6.26E-18	1.21E-13	SRP-dependent cotranslational protein targeting to membrane
**GO:0006119**	121	0.803	6.69E-16	9.67E-12	oxidative phosphorylation
**GO:0022900**	164	0.646	1.65E-15	1.91E-11	electron transport chain
**GO:0006612**	188	0.603	7.84E-15	5.03E-11	protein targeting to membrane

**GO:0071346**	146	0.670	6.33E-15	5.03E-11	cellular response to interferon-gamma
**GO:0072599**	111	0.920	7.06E-15	5.03E-11	establishment of protein localization to endoplasmic reticulum
**GO:0042773**	81	0.957	5.76E-15	5.03E-11	ATP synthesis coupled electron transport
**GO:0042775**	80	0.959	9.35E-15	5.40E-11	mitochondrial ATP synthesis coupled electron transport
**GO:0043299**	493	0.359	1.15E-14	6.04E-11	leukocyte degranulation
**GO:0000184**	117	0.856	1.79E-14	8.62E-11	nuclear-transcribed mRNA catabolic process, nonsense-mediated decay
**GO:0070972**	135	0.777	3.14E-14	1.39E-10	protein localization to endoplasmic reticulum
**GO:0022904**	100	0.812	5.16E-14	2.13E-10	respiratory electron transport chain
**GO:0035459**	19	-1.276	8.96E-14	3.45E-10	vesicle cargo loading
**GO:0034341**	163	0.599	1.42E-13	5.11E-10	response to interferon-gamma
**GO:0050900**	400	0.380	1.63E-13	5.54E-10	leukocyte migration
**GO:0006397**	474	-0.341	3.03E-13	9.73E-10	mRNA processing
**GO:0010257**	58	0.961	8.84E-13	2.56E-09	NADH dehydrogenase complex assembly
**GO:0032981**	58	0.961	8.84E-13	2.56E-09	mitochondrial respiratory chain complex I assembly
**GO:0034976**	274	-0.439	1.58E-12	4.17E-09	response to endoplasmic reticulum stress
**GO:0036230**	464	0.337	1.59E-12	4.17E-09	granulocyte activation
**GO:0042119**	458	0.336	2.70E-12	6.77E-09	neutrophil activation
**GO:0007030**	122	-0.654	2.94E-12	6.80E-09	Golgi organization
**GO:0006120**	46	1.141	2.88E-12	6.80E-09	mitochondrial electron transport, NADH to ubiquinone
**GO:0002446**	460	0.329	6.43E-12	1.43E-08	neutrophil mediated immunity
**GO:0043062**	355	0.367	1.12E-11	2.40E-08	extracellular structure organization
**GO:0002283**	449	0.328	1.29E-11	2.56E-08	neutrophil activation involved in immune response
**GO:0043312**	447	0.329	1.26E-11	2.56E-08	neutrophil degranulation
**GO:0030198**	354	0.364	1.69E-11	3.26E-08	extracellular matrix organization
**GO:0033108**	87	0.750	2.20E-11	4.11E-08	mitochondrial respiratory chain complex assembly
**GO:0016570**	434	-0.327	2.70E-11	4.88E-08	histone modification
**GO:0016569**	448	-0.319	3.78E-11	6.63E-08	covalent chromatin modification
**GO:0050684**	128	-0.576	6.38E-11	1.08E-07	regulation of mRNA processing
**GO:0006909**	256	0.416	1.05E-10	1.74E-07	phagocytosis
**GO:0019083**	173	0.525	1.20E-10	1.93E-07	viral transcription
**GO:0090110**	14	-1.283	2.24E-10	3.50E-07	COPII-coated vesicle cargo loading

**GO:1903311**	308	-0.367	2.56E-10	3.90E-07	regulation of mRNA metabolic process
**GO:0090150**	317	0.358	5.33E-10	7.90E-07	establishment of protein localization to membrane
**GO:0030433**	75	-0.754	6.32E-10	9.13E-07	ubiquitin-dependent ERAD pathway
**GO:0048193**	352	-0.339	6.58E-10	9.28E-07	Golgi vesicle transport
**GO:0002237**	318	0.351	8.76E-10	1.21E-06	response to molecule of bacterial origin
**GO:0002685**	185	0.463	1.04E-09	1.40E-06	regulation of leukocyte migration
**GO:0002694**	476	0.283	1.59E-09	2.08E-06	regulation of leukocyte activation
**GO:0050867**	306	0.349	2.32E-09	2.98E-06	positive regulation of cell activation
**GO:0019080**	187	0.458	2.60E-09	3.27E-06	viral gene expression
**GO:0035966**	190	-0.438	3.17E-09	3.90E-06	response to topologically incorrect protein
**GO:0070661**	271	0.367	3.31E-09	3.99E-06	leukocyte proliferation
**GO:0006413**	186	0.449	5.54E-09	6.54E-06	translational initiation
**GO:0060333**	65	0.753	5.65E-09	6.54E-06	interferon-gamma-mediated signaling pathway
**GO:0032963**	98	0.594	7.76E-09	8.80E-06	collagen metabolic process
**GO:0070126**	89	0.621	9.65E-09	1.07E-05	mitochondrial translational termination
**GO:0008380**	407	-0.288	1.07E-08	1.17E-05	RNA splicing
**GO:0043414**	295	-0.336	1.15E-08	1.23E-05	macromolecule methylation
**GO:0070125**	88	0.626	1.25E-08	1.31E-05	mitochondrial translational elongation
**GO:0010256**	413	-0.285	1.35E-08	1.39E-05	endomembrane system organization
**GO:0051650**	182	-0.431	1.58E-08	1.57E-05	establishment of vesicle localization
**GO:0032496**	307	0.329	1.55E-08	1.57E-05	response to lipopolysaccharide
**GO:0002696**	297	0.334	1.65E-08	1.62E-05	positive regulation of leukocyte activation
**GO:0010498**	459	-0.269	1.71E-08	1.65E-05	proteasomal protein catabolic process
**GO:0036503**	97	-0.599	1.82E-08	1.73E-05	ERAD pathway
**GO:0006888**	203	-0.408	1.89E-08	1.76E-05	endoplasmic reticulum to Golgi vesicle-mediated transport
**GO:0032943**	250	0.363	2.03E-08	1.86E-05	mononuclear cell proliferation
**GO:0043161**	406	-0.284	2.10E-08	1.90E-05	proteasome-mediated ubiquitin-dependent protein catabolic process
**GO:0006986**	169	-0.435	2.81E-08	2.49E-05	response to unfolded protein
**GO:0046651**	248	0.360	2.84E-08	2.49E-05	lymphocyte proliferation
**GO:0002687**	124	0.499	5.55E-08	4.79E-05	positive regulation of leukocyte migration
**GO:0035967**	153	-0.445	6.72E-08	5.71E-05	cellular response to topologically incorrect protein

**GO:0015985**	20	1.201	8.03E-08	6.63E-05	energy coupled proton transport, down electrochemical gradient
**GO:0015986**	20	1.201	8.03E-08	6.63E-05	ATP synthesis coupled proton transport
**GO:0002683**	376	0.281	8.77E-08	7.14E-05	negative regulation of immune system process
**GO:0060326**	268	0.330	1.15E-07	9.21E-05	cell chemotaxis
**GO:1901685**	19	1.047	1.30E-07	1.01E-04	glutathione derivative metabolic process
**GO:1901687**	19	1.047	1.30E-07	1.01E-04	glutathione derivative biosynthetic process
**GO:0016458**	202	-0.377	1.36E-07	1.05E-04	gene silencing
**GO:0051648**	201	-0.379	1.39E-07	1.06E-04	vesicle localization
**GO:0048194**	79	-0.657	1.59E-07	1.18E-04	Golgi vesicle budding
**GO:0016441**	129	-0.470	1.58E-07	1.18E-04	posttranscriptional gene silencing
**GO:0006900**	100	-0.561	1.63E-07	1.19E-04	vesicle budding from membrane
**GO:0045333**	177	0.404	1.68E-07	1.22E-04	cellular respiration
**GO:0032259**	347	-0.281	2.25E-07	1.61E-04	methylation
**GO:0050866**	181	0.392	2.29E-07	1.62E-04	negative regulation of cell activation
**GO:0042776**	19	1.200	2.46E-07	1.71E-04	mitochondrial ATP synthesis coupled proton transport
**GO:0031047**	150	-0.426	2.55E-07	1.76E-04	gene silencing by RNA
**GO:0070663**	207	0.366	2.62E-07	1.78E-04	regulation of leukocyte proliferation
**GO:0002250**	352	0.279	2.77E-07	1.86E-04	adaptive immune response
**GO:0060628**	16	-1.139	3.08E-07	2.04E-04	regulation of ER to Golgi vesicle-mediated transport
**GO:0032944**	197	0.373	3.21E-07	2.11E-04	regulation of mononuclear cell proliferation
**GO:0035194**	128	-0.457	3.71E-07	2.41E-04	post-transcriptional gene silencing by RNA
**GO:0048024**	90	-0.530	3.86E-07	2.45E-04	regulation of mRNA splicing, via spliceosome
**GO:0050920**	202	0.363	3.82E-07	2.45E-04	regulation of chemotaxis
**GO:0043484**	126	-0.451	4.01E-07	2.52E-04	regulation of RNA splicing
**GO:0006903**	91	-0.570	4.17E-07	2.59E-04	vesicle targeting
**GO:0050670**	196	0.370	4.26E-07	2.62E-04	regulation of lymphocyte proliferation
**GO:0033044**	326	-0.284	4.50E-07	2.74E-04	regulation of chromosome organization
**GO:0002695**	160	0.407	4.70E-07	2.83E-04	negative regulation of leukocyte activation
**GO:0042752**	112	-0.481	4.80E-07	2.86E-04	regulation of circadian rhythm
**GO:0071219**	187	0.376	5.04E-07	2.97E-04	cellular response to molecule of bacterial origin
**GO:0006403**	219	-0.343	5.17E-07	3.02E-04	RNA localization
**GO:0042742**	192	0.367	5.79E-07	3.35E-04	defense response to bacterium

**GO:0071222**	181	0.379	6.09E-07	3.48E-04	cellular response to lipopolysaccharide
**GO:0006415**	102	0.504	6.33E-07	3.59E-04	translational termination
**GO:0030518**	125	-0.454	6.42E-07	3.60E-04	intracellular steroid hormone receptor signaling pathway
**GO:0006607**	16	-1.034	6.83E-07	3.80E-04	NLS-bearing protein import into nucleus
**GO:0006405**	129	-0.441	7.13E-07	3.92E-04	RNA export from nucleus
**GO:0031124**	96	-0.507	7.22E-07	3.94E-04	mRNA 3'-end processing
**GO:0032946**	127	0.446	7.95E-07	4.29E-04	positive regulation of mononuclear cell proliferation
**GO:0050727**	317	0.280	9.22E-07	4.93E-04	regulation of inflammatory response
**GO:0051168**	188	-0.360	1.08E-06	5.70E-04	nuclear export
**GO:0071674**	77	0.563	1.09E-06	5.70E-04	mononuclear cell migration
**GO:0050671**	126	0.442	1.11E-06	5.76E-04	positive regulation of lymphocyte proliferation
**GO:0035456**	25	0.985	1.22E-06	6.32E-04	response to interferon-beta
**GO:0032103**	469	0.228	1.31E-06	6.66E-04	positive regulation of response to external stimulus
**GO:0050921**	132	0.429	1.32E-06	6.66E-04	positive regulation of chemotaxis
**GO:0000028**	16	1.331	1.33E-06	6.66E-04	ribosomal small subunit assembly
**GO:0070665**	132	0.428	1.36E-06	6.78E-04	positive regulation of leukocyte proliferation
**GO:0002576**	125	0.446	1.38E-06	6.83E-04	platelet degranulation
**GO:0002886**	53	0.703	1.43E-06	6.98E-04	regulation of myeloid leukocyte mediated immunity
**GO:0016579**	258	-0.305	1.60E-06	7.78E-04	protein deubiquitination
**GO:0046034**	270	0.298	1.68E-06	8.11E-04	ATP metabolic process
**GO:0045576**	57	0.652	1.73E-06	8.28E-04	mast cell activation
**GO:0034620**	134	-0.420	1.79E-06	8.42E-04	cellular response to unfolded protein
**GO:0030098**	332	0.266	1.79E-06	8.42E-04	lymphocyte differentiation
**GO:0007623**	208	-0.334	2.02E-06	9.41E-04	circadian rhythm
**GO:0009048**	17	-1.075	2.09E-06	9.68E-04	dosage compensation by inactivation of X chromosome
**GO:0000375**	324	-0.265	2.29E-06	1.05E-03	RNA splicing, via transesterification reactions
**GO:1900034**	76	-0.546	2.33E-06	1.06E-03	regulation of cellular response to heat
**GO:0002521**	488	0.217	2.55E-06	1.15E-03	leukocyte differentiation
**GO:0050707**	48	0.688	2.56E-06	1.15E-03	regulation of cytokine secretion
**GO:0002448**	47	0.716	2.62E-06	1.17E-03	mast cell mediated immunity
**GO:2000249**	38	0.836	2.68E-06	1.18E-03	regulation of actin cytoskeleton reorganization
**GO:2001056**	135	0.408	2.86E-06	1.25E-03	positive regulation of cysteine-type endopeptidase activity

**GO:0070646**	274	-0.288	2.98E-06	1.30E-03	protein modification by small protein removal
**GO:0002279**	46	0.704	3.29E-06	1.42E-03	mast cell activation involved in immune response
**GO:0006479**	175	-0.354	3.45E-06	1.47E-03	protein methylation
**GO:0008213**	175	-0.354	3.45E-06	1.47E-03	protein alkylation
**GO:0000377**	321	-0.261	3.76E-06	1.57E-03	RNA splicing, via transesterification reactions with bulged adenosine as nucleophile
**GO:0000398**	321	-0.261	3.76E-06	1.57E-03	mRNA splicing, via spliceosome
**GO:0048199**	73	-0.589	3.84E-06	1.59E-03	vesicle targeting, to, from or within Golgi
**GO:0042110**	431	0.226	3.84E-06	1.59E-03	T cell activation
**GO:1901568**	140	0.394	4.00E-06	1.64E-03	fatty acid derivative metabolic process
**GO:0016571**	137	-0.398	4.08E-06	1.66E-03	histone methylation
**GO:0030968**	117	-0.435	4.16E-06	1.68E-03	endoplasmic reticulum unfolded protein response
**GO:0052547**	383	0.238	4.63E-06	1.86E-03	regulation of peptidase activity
**GO:0043303**	45	0.702	4.68E-06	1.87E-03	mast cell degranulation
**GO:0006605**	417	0.228	4.73E-06	1.87E-03	protein targeting
**GO:0018205**	378	-0.239	4.80E-06	1.89E-03	peptidyl-lysine modification
**GO:0051236**	185	-0.340	4.93E-06	1.93E-03	establishment of RNA localization
**GO:0046185**	13	1.069	5.24E-06	2.03E-03	aldehyde catabolic process
**GO:0001819**	356	0.245	5.32E-06	2.05E-03	positive regulation of cytokine production
**GO:0006901**	69	-0.605	5.45E-06	2.08E-03	vesicle coating
**GO:0035195**	119	-0.423	5.46E-06	2.08E-03	gene silencing by miRNA
**GO:0051251**	254	0.289	5.96E-06	2.25E-03	positive regulation of lymphocyte activation
**GO:1905517**	46	0.701	6.01E-06	2.26E-03	macrophage migration
**GO:0036500**	10	-1.246	6.44E-06	2.39E-03	ATF6-mediated unfolded protein response
**GO:0010952**	172	0.350	6.41E-06	2.39E-03	positive regulation of peptidase activity
**GO:0032527**	44	-0.732	6.65E-06	2.45E-03	protein exit from endoplasmic reticulum
**GO:0006378**	43	-0.696	6.74E-06	2.45E-03	mRNA polyadenylation
**GO:0043631**	43	-0.696	6.74E-06	2.45E-03	RNA polyadenylation
**GO:0030522**	267	-0.279	7.15E-06	2.58E-03	intracellular receptor signaling pathway
**GO:0042116**	88	0.495	7.22E-06	2.59E-03	macrophage activation
**GO:0042098**	173	0.346	7.34E-06	2.62E-03	T cell proliferation
**GO:0033003**	39	0.739	7.39E-06	2.62E-03	regulation of mast cell activation
**GO:0033006**	30	0.863	7.47E-06	2.63E-03	regulation of mast cell activation involved in immune response
**GO:2000116**	219	0.307	7.70E-06	2.70E-03	regulation of cysteine-type endopeptidase activity

**GO:0097529**	184	0.334	7.83E-06	2.73E-03	myeloid leukocyte migration
**GO:0010950**	153	0.365	8.13E-06	2.81E-03	positive regulation of endopeptidase activity
**GO:0000956**	194	0.330	8.33E-06	2.87E-03	nuclear-transcribed mRNA catabolic process
**GO:0042149**	40	-0.697	8.72E-06	2.95E-03	cellular response to glucose starvation
**GO:0002697**	351	0.241	8.69E-06	2.95E-03	regulation of immune effector process
**GO:0002699**	188	0.330	8.65E-06	2.95E-03	positive regulation of immune effector process
**GO:0030595**	196	0.322	9.71E-06	3.26E-03	leukocyte chemotaxis
**GO:0032786**	28	-0.808	9.95E-06	3.32E-03	positive regulation of DNA-templated transcription, elongation
**GO:0006611**	173	-0.340	1.02E-05	3.34E-03	protein export from nucleus
**GO:0045785**	386	0.228	1.02E-05	3.34E-03	positive regulation of cell adhesion
**GO:0031532**	99	0.456	1.02E-05	3.34E-03	actin cytoskeleton reorganization
**GO:0006913**	329	-0.246	1.05E-05	3.44E-03	nucleocytoplasmic transport
**GO:0050663**	61	0.576	1.07E-05	3.48E-03	cytokine secretion
**GO:0006409**	32	-0.795	1.14E-05	3.63E-03	tRNA export from nucleus
**GO:0071431**	32	-0.795	1.14E-05	3.63E-03	tRNA-containing ribonucleoprotein complex export from nucleus
**GO:0071426**	121	-0.402	1.13E-05	3.63E-03	ribonucleoprotein complex export from nucleus
**GO:0043304**	29	0.866	1.15E-05	3.64E-03	regulation of mast cell degranulation
**GO:0043280**	118	0.408	1.20E-05	3.80E-03	positive regulation of cysteine-type endopeptidase activity involved in apoptotic process
**GO:0031123**	133	-0.381	1.22E-05	3.82E-03	RNA 3'-end processing
**GO:0007229**	106	0.436	1.29E-05	4.04E-03	integrin-mediated signaling pathway
**GO:0000380**	72	-0.519	1.33E-05	4.11E-03	alternative mRNA splicing, via spliceosome
**GO:0007599**	311	0.250	1.33E-05	4.11E-03	hemostasis
**GO:0051169**	332	-0.241	1.40E-05	4.29E-03	nuclear transport
**GO:0045766**	161	0.345	1.45E-05	4.44E-03	positive regulation of angiogenesis
**GO:0017148**	123	-0.401	1.52E-05	4.62E-03	negative regulation of translation
**GO:0051249**	395	0.221	1.53E-05	4.64E-03	regulation of lymphocyte activation
**GO:0006110**	72	-0.524	1.54E-05	4.65E-03	regulation of glycolytic process
**GO:0090114**	71	-0.548	1.61E-05	4.79E-03	COPII-coated vesicle budding
**GO:0050657**	182	-0.323	1.62E-05	4.79E-03	nucleic acid transport
**GO:0050658**	182	-0.323	1.62E-05	4.79E-03	RNA transport

**GO:0002688**	106	0.426	1.75E-05	5.15E-03	regulation of leukocyte chemotaxis
**GO:0150076**	63	0.572	1.81E-05	5.31E-03	neuroinflammatory response
**GO:0031331**	345	-0.233	2.01E-05	5.84E-03	positive regulation of cellular catabolic process
**GO:0034248**	381	-0.222	2.00E-05	5.84E-03	regulation of cellular amide metabolic process
**GO:0006406**	105	-0.418	2.07E-05	5.87E-03	mRNA export from nucleus
**GO:0071427**	105	-0.418	2.07E-05	5.87E-03	mRNA-containing ribonucleoprotein complex export from nucleus
**GO:0071166**	122	-0.388	2.07E-05	5.87E-03	ribonucleoprotein complex localization
**GO:0034504**	251	-0.272	2.06E-05	5.87E-03	protein localization to nucleus
**GO:0007596**	306	0.247	2.06E-05	5.87E-03	blood coagulation
**GO:0006801**	66	0.537	2.15E-05	6.05E-03	superoxide metabolic process
**GO:0030970**	28	-0.809	2.17E-05	6.06E-03	retrograde protein transport, ER to cytosol
**GO:1903513**	28	-0.809	2.17E-05	6.06E-03	endoplasmic reticulum to cytosol transport
**GO:1901342**	310	0.243	2.23E-05	6.17E-03	regulation of vasculature development
**GO:0050764**	91	0.455	2.23E-05	6.17E-03	regulation of phagocytosis
**GO:0043401**	176	-0.324	2.27E-05	6.24E-03	steroid hormone mediated signaling pathway
**GO:0072676**	94	0.454	2.29E-05	6.27E-03	lymphocyte migration
**GO:0048511**	297	-0.249	2.33E-05	6.33E-03	rhythmic process
**GO:0032543**	134	0.370	2.33E-05	6.33E-03	mitochondrial translation
**GO:0050728**	128	0.381	2.35E-05	6.33E-03	negative regulation of inflammatory response
**GO:0002431**	84	0.481	2.36E-05	6.33E-03	Fc receptor mediated stimulatory signaling pathway
**GO:1905521**	35	0.786	2.37E-05	6.35E-03	regulation of macrophage migration
**GO:0007159**	313	0.242	2.46E-05	6.53E-03	leukocyte cell-cell adhesion
**GO:0009595**	28	0.803	2.46E-05	6.53E-03	detection of biotic stimulus
**GO:0071216**	211	0.295	2.48E-05	6.55E-03	cellular response to biotic stimulus
**GO:1904356**	59	-0.555	2.50E-05	6.57E-03	regulation of telomere maintenance via telomere lengthening
**GO:0150079**	10	1.198	2.53E-05	6.62E-03	negative regulation of neuroinflammatory response
**GO:0097064**	36	-0.713	2.57E-05	6.65E-03	ncRNA export from nucleus
**GO:0052548**	358	0.225	2.58E-05	6.65E-03	regulation of endopeptidase activity
**GO:0050817**	310	0.242	2.56E-05	6.65E-03	coagulation
**GO:0000381**	61	-0.533	2.66E-05	6.80E-03	regulation of alternative mRNA splicing, via spliceosome

**GO:0042102**	90	0.451	2.66E-05	6.80E-03	positive regulation of T cell proliferation
**GO:1903039**	205	0.298	2.71E-05	6.90E-03	positive regulation of leukocyte cell-cell adhesion
**GO:0045765**	280	0.253	2.73E-05	6.91E-03	regulation of angiogenesis
**GO:0015931**	227	-0.280	2.83E-05	7.14E-03	nucleobase-containing compound transport
**GO:0043300**	43	0.677	2.85E-05	7.15E-03	regulation of leukocyte degranulation
**GO:0060968**	108	-0.408	2.89E-05	7.19E-03	regulation of gene silencing
**GO:0001774**	43	0.691	2.90E-05	7.19E-03	microglial cell activation
**GO:0002269**	43	0.691	2.90E-05	7.19E-03	leukocyte activation involved in inflammatory response
**GO:0006487**	77	-0.482	2.97E-05	7.35E-03	protein N-linked glycosylation
**GO:0002888**	32	0.773	3.07E-05	7.55E-03	positive regulation of myeloid leukocyte mediated immunity
**GO:0051028**	141	-0.353	3.27E-05	8.01E-03	mRNA transport
**GO:0034660**	436	-0.202	3.30E-05	8.04E-03	ncRNA metabolic process
**GO:0006417**	331	-0.231	3.47E-05	8.42E-03	regulation of translation
**GO:0016573**	149	-0.343	3.49E-05	8.44E-03	histone acetylation
**GO:0032210**	52	-0.579	3.64E-05	8.76E-03	regulation of telomere maintenance via telomerase
**GO:0002690**	84	0.460	3.86E-05	9.26E-03	positive regulation of leukocyte chemotaxis
**GO:0034968**	113	-0.392	3.88E-05	9.27E-03	histone lysine methylation
**GO:0018210**	122	-0.379	4.12E-05	9.77E-03	peptidyl-threonine modification
**GO:0034249**	140	-0.354	4.12E-05	9.77E-03	negative regulation of cellular amide metabolic process
**GO:0006984**	46	-0.617	4.16E-05	9.77E-03	ER-nucleus signaling pathway
**GO:0038094**	82	0.472	4.15E-05	9.77E-03	Fc-gamma receptor signaling pathway
**GO:0000186**	55	-0.572	4.22E-05	9.83E-03	activation of MAPKK activity
**GO:0022409**	241	0.267	4.22E-05	9.83E-03	positive regulation of cell-cell adhesion
**GO:0060147**	90	-0.438	4.32E-05	9.98E-03	regulation of posttranscriptional gene silencing
**GO:0060966**	90	-0.438	4.32E-05	9.98E-03	regulation of gene silencing by RNA
**GO:0060337**	67	0.520	4.37E-05	1.00E-02	type I interferon signaling pathway
**GO:0071357**	67	0.520	4.37E-05	1.00E-02	cellular response to type I interferon
**GO:0051031**	34	-0.732	4.40E-05	1.01E-02	tRNA transport
**GO:0000209**	299	-0.239	4.43E-05	1.01E-02	protein polyubiquitination
**GO:0032102**	333	0.227	4.44E-05	1.01E-02	negative regulation of response to external stimulus
**GO:0007007**	41	0.659	4.54E-05	1.03E-02	inner mitochondrial membrane organization

**GO:0071108**	33	-0.826	4.74E-05	1.06E-02	protein K48-linked deubiquitination
**GO:0043470**	81	-0.461	4.74E-05	1.06E-02	regulation of carbohydrate catabolic process
**GO:0032680**	103	0.408	4.96E-05	1.11E-02	regulation of tumor necrosis factor production
**GO:0045943**	22	-0.907	5.12E-05	1.14E-02	positive regulation of transcription by RNA polymerase I
**GO:0002703**	180	0.306	5.18E-05	1.14E-02	regulation of leukocyte mediated immunity
**GO:0006690**	87	0.439	5.17E-05	1.14E-02	icosanoid metabolic process
**GO:0030574**	42	0.619	5.22E-05	1.15E-02	collagen catabolic process
**GO:0010935**	12	1.264	5.36E-05	1.17E-02	regulation of macrophage cytokine production
**GO:0007004**	66	-0.497	5.44E-05	1.18E-02	telomere maintenance via telomerase
**GO:0030335**	479	0.187	5.43E-05	1.18E-02	positive regulation of cell migration
**GO:0071887**	99	0.409	5.45E-05	1.18E-02	leukocyte apoptotic process
**GO:0043543**	233	-0.267	5.58E-05	1.20E-02	protein acylation
**GO:0050830**	67	0.504	5.82E-05	1.25E-02	defense response to Gram-positive bacterium
**GO:1904358**	37	-0.654	5.93E-05	1.27E-02	positive regulation of telomere maintenance via telomere lengthening
**GO:0031663**	54	0.570	5.93E-05	1.27E-02	lipopolysaccharide-mediated signaling pathway
**GO:0032204**	77	-0.464	6.08E-05	1.29E-02	regulation of telomere maintenance
**GO:0002433**	78	0.472	6.28E-05	1.32E-02	immune response-regulating cell surface receptor signaling pathway involved in phagocytosis
**GO:0038096**	78	0.472	6.28E-05	1.32E-02	Fc-gamma receptor signaling pathway involved in phagocytosis
**GO:0018393**	153	-0.327	6.33E-05	1.33E-02	internal peptidyl-lysine acetylation
**GO:0032640**	107	0.394	6.32E-05	1.33E-02	tumor necrosis factor production
**GO:0010833**	77	-0.457	6.36E-05	1.33E-02	telomere maintenance via telomere lengthening
**GO:0001776**	84	0.444	6.40E-05	1.33E-02	leukocyte homeostasis
**GO:0043624**	211	0.278	6.64E-05	1.37E-02	cellular protein complex disassembly
**GO:0002335**	24	0.802	6.65E-05	1.37E-02	mature B cell differentiation
**GO:0006109**	190	-0.293	6.76E-05	1.39E-02	regulation of carbohydrate metabolic process
**GO:0048207**	65	-0.526	7.50E-05	1.53E-02	vesicle targeting, rough ER to cis-Golgi
**GO:0048208**	65	-0.526	7.50E-05	1.53E-02	COPII vesicle coating
**GO:0022617**	74	0.469	7.78E-05	1.58E-02	extracellular matrix disassembly

**GO:0006959**	190	0.289	8.13E-05	1.65E-02	humoral immune response
**GO:0150077**	34	0.735	8.25E-05	1.67E-02	regulation of neuroinflammatory response
**GO:0046456**	43	0.599	8.40E-05	1.69E-02	icosanoid biosynthetic process
**GO:1903555**	106	0.388	8.68E-05	1.74E-02	regulation of tumor necrosis factor superfamily cytokine production
**GO:1903312**	73	-0.464	8.73E-05	1.75E-02	negative regulation of mRNA metabolic process
**GO:2000147**	500	0.178	8.90E-05	1.77E-02	positive regulation of cell motility
**GO:0050829**	58	0.519	8.97E-05	1.78E-02	defense response to Gram-negative bacterium
**GO:0050777**	129	0.349	9.30E-05	1.84E-02	negative regulation of immune response
**GO:0043534**	112	0.372	9.40E-05	1.85E-02	blood vessel endothelial cell migration
**GO:0018022**	126	-0.350	9.91E-05	1.95E-02	peptidyl-lysine methylation
**GO:1904018**	180	0.292	1.01E-04	1.97E-02	positive regulation of vasculature development
**GO:0043542**	197	0.279	1.03E-04	2.01E-02	endothelial cell migration
**GO:0042129**	145	0.327	1.05E-04	2.04E-02	regulation of T cell proliferation
**GO:0071706**	110	0.375	1.08E-04	2.10E-02	tumor necrosis factor superfamily cytokine production
**GO:0019371**	11	1.118	1.09E-04	2.11E-02	cyclooxygenase pathway
**GO:0043281**	196	0.280	1.10E-04	2.12E-02	regulation of cysteine-type endopeptidase activity involved in apoptotic process
**GO:0009755**	221	-0.262	1.14E-04	2.18E-02	hormone-mediated signaling pathway
**GO:0018209**	294	-0.227	1.18E-04	2.25E-02	peptidyl-serine modification
**GO:0036507**	20	-1.023	1.21E-04	2.29E-02	protein demannosylation
**GO:0036508**	20	-1.023	1.21E-04	2.29E-02	protein alpha-1,2-demannosylation
**GO:0043984**	20	-0.775	1.24E-04	2.34E-02	histone H4-K16 acetylation
**GO:2001252**	167	-0.300	1.24E-04	2.34E-02	positive regulation of chromosome organization
**GO:0045471**	130	0.340	1.23E-04	2.34E-02	response to ethanol
**GO:0006278**	68	-0.466	1.25E-04	2.34E-02	RNA-dependent DNA biosynthetic process
**GO:0099024**	58	0.512	1.25E-04	2.35E-02	plasma membrane invagination
**GO:0009206**	55	0.533	1.28E-04	2.38E-02	purine ribonucleoside triphosphate biosynthetic process
**GO:0009145**	56	0.527	1.29E-04	2.39E-02	purine nucleoside triphosphate biosynthetic process
**GO:0043031**	15	0.982	1.31E-04	2.42E-02	negative regulation of macrophage activation
**GO:0033962**	20	-0.845	1.32E-04	2.44E-02	P-body assembly

**GO:0050729**	135	0.333	1.33E-04	2.44E-02	positive regulation of inflammatory response
**GO:0033169**	13	-0.993	1.35E-04	2.47E-02	histone H3-K9 demethylation
**GO:0001525**	481	0.177	1.35E-04	2.47E-02	angiogenesis
**GO:0032212**	34	-0.648	1.37E-04	2.49E-02	positive regulation of telomere maintenance via telomerase
**GO:0018105**	274	-0.233	1.37E-04	2.49E-02	peptidyl-serine phosphorylation
**GO:0034453**	26	-0.752	1.39E-04	2.52E-02	microtubule anchoring
**GO:0018107**	114	-0.364	1.40E-04	2.52E-02	peptidyl-threonine phosphorylation
**GO:0050870**	191	0.278	1.48E-04	2.66E-02	positive regulation of T cell activation
**GO:0005978**	49	-0.549	1.51E-04	2.69E-02	glycogen biosynthetic process
**GO:0009250**	49	-0.549	1.51E-04	2.69E-02	glucan biosynthetic process
**GO:0042407**	29	0.741	1.55E-04	2.76E-02	cristae formation
**GO:0060964**	87	-0.411	1.55E-04	2.76E-02	regulation of gene silencing by miRNA
**GO:0001516**	24	0.794	1.61E-04	2.84E-02	prostaglandin biosynthetic process
**GO:0046457**	24	0.794	1.61E-04	2.84E-02	prostanoid biosynthetic process
**GO:0006414**	138	0.326	1.62E-04	2.86E-02	translational elongation
**GO:0018279**	33	-0.688	1.65E-04	2.89E-02	protein N-linked glycosylation via asparagine
**GO:0007029**	62	-0.504	1.65E-04	2.89E-02	endoplasmic reticulum organization
**GO:0045730**	34	0.736	1.66E-04	2.89E-02	respiratory burst
**GO:0002679**	13	1.168	1.69E-04	2.94E-02	respiratory burst involved in defense response
**GO:0042246**	70	0.448	1.70E-04	2.95E-02	tissue regeneration
**GO:0051250**	132	0.332	1.74E-04	3.02E-02	negative regulation of lymphocyte activation
**GO:0016050**	320	-0.212	1.81E-04	3.13E-02	vesicle organization
**GO:0097237**	110	0.357	1.87E-04	3.21E-02	cellular response to toxic substance
**GO:0043302**	23	0.801	1.93E-04	3.31E-02	positive regulation of leukocyte degranulation
**GO:0043537**	32	0.649	1.95E-04	3.34E-02	negative regulation of blood vessel endothelial cell migration
**GO:0002700**	119	0.348	1.98E-04	3.38E-02	regulation of production of molecular mediator of immune response
**GO:2000251**	19	0.827	2.05E-04	3.49E-02	positive regulation of actin cytoskeleton reorganization
**GO:0007020**	25	-0.726	2.19E-04	3.69E-02	microtubule nucleation
**GO:0018196**	34	-0.660	2.18E-04	3.69E-02	peptidyl-asparagine modification
**GO:1901570**	88	0.397	2.19E-04	3.69E-02	fatty acid derivative biosynthetic process
**GO:0043535**	88	0.398	2.19E-04	3.69E-02	regulation of blood vessel endothelial cell migration

**GO:0031348**	185	0.275	2.25E-04	3.77E-02	negative regulation of defense response
**GO:0006911**	49	0.539	2.26E-04	3.78E-02	phagocytosis, engulfment
**GO:0018394**	161	-0.294	2.27E-04	3.79E-02	peptidyl-lysine acetylation
**GO:0032956**	317	0.210	2.29E-04	3.81E-02	regulation of actin cytoskeleton organization
**GO:0009620**	37	0.657	2.36E-04	3.90E-02	response to fungus
**GO:0048246**	34	0.661	2.37E-04	3.92E-02	macrophage chemotaxis
**GO:0050832**	24	0.776	2.39E-04	3.93E-02	defense response to fungus
**GO:0030521**	57	-0.497	2.44E-04	4.00E-02	androgen receptor signaling pathway
**GO:0006892**	101	-0.376	2.48E-04	4.05E-02	post-Golgi vesicle-mediated transport
**GO:0006475**	158	-0.294	2.50E-04	4.07E-02	internal protein amino acid acetylation
**GO:0030258**	228	-0.244	2.50E-04	4.07E-02	lipid modification
**GO:0034340**	70	0.454	2.54E-04	4.13E-02	response to type I interferon
**GO:0001510**	76	-0.418	2.57E-04	4.17E-02	RNA methylation
**GO:1990748**	102	0.362	2.61E-04	4.21E-02	cellular detoxification
**GO:0035329**	37	-0.589	2.67E-04	4.28E-02	hippo signaling
**GO:0033143**	74	-0.434	2.66E-04	4.28E-02	regulation of intracellular steroid hormone receptor signaling pathway
**GO:0042060**	462	0.172	2.68E-04	4.29E-02	wound healing
**GO:0031440**	28	-0.706	2.74E-04	4.36E-02	regulation of mRNA 3'-end processing
**GO:0001906**	128	0.325	2.73E-04	4.36E-02	cell killing
**GO:0033008**	15	0.890	2.79E-04	4.42E-02	positive regulation of mast cell activation involved in immune response
**GO:0043306**	15	0.890	2.79E-04	4.42E-02	positive regulation of mast cell degranulation
**GO:0045619**	160	0.290	2.84E-04	4.49E-02	regulation of lymphocyte differentiation
**GO:0043123**	176	0.276	2.86E-04	4.51E-02	positive regulation of I-kappaB kinase/NF-kappaB signaling
**GO:0071675**	43	0.565	2.88E-04	4.52E-02	regulation of mononuclear cell migration
**GO:0016574**	45	-0.551	2.90E-04	4.54E-02	histone ubiquitination
**GO:0046831**	13	-1.010	2.95E-04	4.61E-02	regulation of RNA export from nucleus
**GO:0032784**	45	-0.547	2.99E-04	4.66E-02	regulation of DNA-templated transcription, elongation
**GO:0016577**	25	-0.715	3.02E-04	4.69E-02	histone demethylation
**GO:0000723**	141	-0.306	3.07E-04	4.75E-02	telomere maintenance
**GO:0009451**	149	-0.297	3.08E-04	4.76E-02	RNA modification
**GO:0043255**	87	-0.391	3.23E-04	4.97E-02	regulation of carbohydrate biosynthetic process

Molecular functions in Genetic group 2 in the most efficient group.					
**Go term**	**Number of genes**	**LOR***	**pvalue**	**padj**	**Molecular functions**
**GO:0003735**	155	1.165	4.37E-25	3.76E-20	structural constituent of ribosome
**GO:0003954**	38	1.355	9.97E-09	2.15E-05	NADH dehydrogenase activity
**GO:0008137**	38	1.355	9.97E-09	2.15E-05	NADH dehydrogenase (ubiquinone) activity
**GO:0050136**	38	1.355	9.97E-09	2.15E-05	NADH dehydrogenase (quinone) activity
**GO:0016655**	50	1.171	2.22E-02	3.83E-13	oxidoreductase activity, acting on NAD(P)H, quinone or similar compound as acceptor
**GO:0101005**	83	-0.783	2.71E-13	3.89E-10	ubiquitinyl hydrolase activity
**GO:0004843**	80	-0.775	1.29E-12	1.59E-09	thiol-dependent ubiquitin-specific protease activity
**GO:0008242**	90	-0.711	3.03E+03	3.26E-09	omega peptidase activity
**GO:0005201**	157	0.480	1.71E-11	1.64E-07	extracellular matrix structural constituent
**GO:0004842**	367	-0.317	2.48E-10	2.14E-07	ubiquitin-protein transferase activity
**GO:0016651**	95	0.618	3.45E-10	2.48E-07	oxidoreductase activity, acting on NAD(P)H
**GO:0019787**	390	-0.306	3.25E-10	2.48E-07	ubiquitin-like protein transferase activity
**GO:0003729**	235	-0.386	6.56E-10	4.35E-07	mRNA binding
**GO:0030545**	444	0.273	1.31E-09	8.05E-07	receptor regulator activity
**GO:0030546**	418	0.280	1.48E-09	8.50E-07	signaling receptor activator activity
**GO:0048018**	412	0.280	1.87E-09	1.01E-06	receptor ligand activity
**GO:0008139**	20	-1.074	3.65E-09	1.85E-06	nuclear localization sequence binding
**GO:0043021**	130	-0.483	7.02E-09	3.36E-06	ribonucleoprotein complex binding
**GO:0009055**	101	0.544	8.44E-09	3.83E-06	electron transfer activity
**GO:0046933**	15	1.187	1.08E-08	4.64E-06	proton-transporting ATP synthase activity, rotational mechanism
**GO:0038187**	12	1.256	2.72E-08	1.12E-04	pattern recognition receptor activity
**GO:0042393**	185	-0.374	5.20E-08	2.04E-04	histone binding
**GO:0004386**	139	-0.425	9.99E-08	3.75E-04	helicase activity
**GO:0004402**	60	-0.624	1.07E-07	3.84E-04	histone acetyltransferase activity
**GO:0005539**	199	0.342	1.72E-07	5.75E-04	glycosaminoglycan binding
**GO:0019865**	20	1.025	1.80E-07	5.75E-04	immunoglobulin binding
**GO:1990380**	14	-1.369	1.75E-07	5.75E-04	Lys48-specific deubiquitinase activity

**GO:0016887**	402	-0.241	1.96E-07	6.04E-04	ATPase activity
**GO:0031624**	31	-0.894	2.44E-07	7.25E-04	ubiquitin conjugating enzyme binding
**GO:0004674**	430	-0.228	3.15E-07	9.04E-04	protein serine/threonine kinase activity
**GO:0061659**	221	-0.315	4.16E-07	1.16E-03	ubiquitin-like protein ligase activity
**GO:0003713**	313	-0.258	6.30E-07	1.70E-03	transcription coactivator activity
**GO:0005178**	126	0.406	6.75E-07	1.76E-03	integrin binding
**GO:0140030**	138	-0.391	7.40E-07	1.88E-03	modification-dependent protein binding
**GO:0043022**	56	-0.605	9.22E-07	2.21E-03	ribosome binding
**GO:0061630**	216	-0.307	9.22E-07	2.21E-03	ubiquitin protein ligase activity
**GO:0061733**	62	-0.561	9.86E-07	2.30E-03	peptide-lysine-N-acetyltransferase activity
**GO:0070492**	11	1.194	1.23E-06	2.79E-03	oligosaccharide binding
**GO:0044390**	37	-0.747	1.96E-06	4.33E-03	ubiquitin-like protein conjugating enzyme binding
**GO:0034212**	71	-0.512	2.02E-06	4.36E-03	peptide N-acetyltransferase activity
**GO:0032452**	23	-0.845	2.69E-06	5.52E-03	histone demethylase activity
**GO:0140457**	23	-0.845	2.69E-06	5.52E-03	protein demethylase activity
**GO:0004364**	24	0.796	4.65E-06	9.33E-03	glutathione transferase activity
**GO:0043295**	10	1.109	5.55E-06	1.06E-02	glutathione binding
**GO:1900750**	10	1.109	5.55E-06	1.06E-02	oligopeptide binding
**GO:0019843**	61	0.536	5.85E-06	1.10E-02	rRNA binding
**GO:0032454**	11	-1.060	6.85E-06	1.26E-02	histone demethylase activity (H3-K9 specific)
**GO:0070063**	59	-0.535	7.41E-06	1.33E-02	RNA polymerase binding
**GO:0140098**	300	-0.225	1.16E-04	2.04E-02	catalytic activity, acting on RNA
**GO:0008757**	148	-0.317	1.29E-04	2.20E-02	S-adenosylmethionine-dependent methyltransferase activity
**GO:0016741**	212	-0.265	1.30E-04	2.20E-02	transferase activity, transferring one-carbon groups
**GO:0005125**	185	0.282	1.36E-04	2.25E-02	cytokine activity
**GO:0008201**	146	0.316	1.47E-04	2.39E-02	heparin binding
**GO:0001540**	73	0.453	1.71E-04	2.71E-02	amyloid-beta binding
**GO:0008168**	202	-0.266	1.73E-04	2.71E-02	methyltransferase activity
**GO:0001098**	56	-0.510	1.98E-04	2.95E-02	basal transcription machinery binding
**GO:0001099**	56	-0.510	1.98E-04	2.95E-02	basal RNA polymerase II transcription machinery binding
**GO:0043175**	44	-0.581	1.95E-04	2.95E-02	RNA polymerase core enzyme binding
**GO:0016790**	35	0.615	2.12E-04	3.09E-02	thiolester hydrolase activity
**GO:0000993**	37	-0.630	2.15E-04	3.09E-02	RNA polymerase II complex binding

**GO:0016811**	67	0.447	2.53E-04	3.55E-02	hydrolase activity, acting on carbon-nitrogen (but not peptide) bonds, in linear amides
**GO:0008080**	83	-0.405	2.55E-04	3.55E-02	N-acetyltransferase activity
**GO:0019213**	41	0.578	2.91E-04	3.99E-02	deacetylase activity
**GO:0042800**	16	-0.940	3.12E-04	4.14E-02	histone methyltransferase activity (H3-K4 specific)
**GO:0050681**	43	-0.559	3.08E-04	4.14E-02	androgen receptor binding
**Cellular components in Genetic group 2 in the most efficient group.**					
**Go term**	**Number of genes**	**LOR***	**pvalue**	**padj**	**Cellular components**
**GO:0044391**	182	1.007	7.02E-20	3.81E-17	ribosomal subunit
**GO:0098798**	239	0.692	5.93E-24	1.61E-20	mitochondrial protein complex
**GO:0005840**	229	0.723	2.60E-23	4.71E-20	ribosome
**GO:0098800**	116	0.996	6.21E-23	8.43E-20	inner mitochondrial membrane protein complex
**GO:0015934**	115	0.937	1.16E-22	1.26E-19	large ribosomal subunit
**GO:0022625**	58	1.194	1.22E-19	1.10E-16	cytosolic large ribosomal subunit
**GO:0005747**	43	1.288	7.20E-18	5.59E-15	mitochondrial respiratory chain complex I
**GO:0030964**	43	1.288	1.46E-17	9.93E-15	NADH dehydrogenase complex
**GO:0045271**	43	1.288	6.69E-17	3.31E-14	respiratory chain complex I
**GO:0022626**	105	1.114	6.69E-17	3.31E-14	cytosolic ribosome
**GO:0098803**	69	1.159	6.69E-17	3.31E-14	respiratory chain complex
**GO:0005746**	74	1.041	1.29E-16	5.83E-14	mitochondrial respirasome
**GO:0070469**	81	0.997	2.92E-16	1.22E-13	respirasome
**GO:0062023**	375	0.425	5.11E-16	1.98E-13	collagen-containing extracellular matrix
**GO:0031012**	488	0.368	1.48E-15	5.36E-13	extracellular matrix
**GO:0005743**	435	0.380	9.92E-15	3.37E-12	mitochondrial inner membrane
**GO:0005753**	18	1.524	2.03E-14	6.11E-12	mitochondrial proton-transporting ATP synthase complex
**GO:0045259**	18	1.524	2.03E-14	6.11E-12	proton-transporting ATP synthase complex
**GO:0015935**	70	1.092	4.97E-14	1.42E-11	small ribosomal subunit
**GO:1990204**	101	0.726	2.31E-13	6.27E-10	oxidoreductase complex
**GO:0019866**	492	0.299	8.54E-12	2.21E-09	organelle inner membrane
**GO:0000313**	86	0.703	1.47E-11	3.47E-09	organellar ribosome
**GO:0005761**	86	0.703	1.47E-11	3.47E-09	mitochondrial ribosome
**GO:0060205**	307	0.331	1.42E-09	3.20E-07	cytoplasmic vesicle lumen
**GO:0005925**	400	0.283	3.10E-09	6.74E-07	focal adhesion
**GO:0034774**	303	0.322	3.93E-09	8.22E-07	secretory granule lumen
**GO:0030055**	407	0.277	4.85E-09	9.50E-07	cell-substrate junction
**GO:0031983**	309	0.317	4.90E-09	9.50E-07	vesicle lumen
**GO:0005775**	158	0.433	1.01E-08	1.90E-06	vacuolar lumen

**GO:0034708**	108	-0.522	1.29E-08	2.33E-05	methyltransferase complex
**GO:0022627**	42	1.331	1.39E-08	2.43E-06	cytosolic small ribosomal subunit
**GO:0070820**	151	0.425	3.38E-08	5.73E-06	tertiary granule
**GO:0000315**	56	0.671	4.96E-08	7.81E-06	organellar large ribosomal subunit
**GO:0005762**	56	0.671	4.96E-08	7.81E-06	mitochondrial large ribosomal subunit
**GO:0016607**	380	-0.262	5.03E-08	7.81E-06	nuclear speck
**GO:0005798**	157	-0.404	8.54E-08	1.29E-04	Golgi-associated vesicle
**GO:0043202**	92	0.520	8.90E-08	1.31E-04	lysosomal lumen
**GO:0008023**	52	-0.687	1.65E-07	2.36E-04	transcription elongation factor complex
**GO:0000151**	272	-0.294	1.86E-07	2.59E-04	ubiquitin ligase complex
**GO:0035097**	81	-0.551	1.97E-07	2.68E-04	histone methyltransferase complex
**GO:0098552**	466	0.223	2.34E-07	3.10E-04	side of membrane
**GO:0035770**	206	-0.332	2.64E-07	3.41E-04	ribonucleoprotein granule
**GO:0005581**	93	0.484	3.68E-07	4.65E-04	collagen trimer
**GO:0031965**	289	-0.274	4.09E-07	5.05E-04	nuclear membrane
**GO:0042581**	151	0.374	5.66E-07	6.83E-04	specific granule
**GO:1904724**	52	0.634	6.43E-07	7.59E-04	tertiary granule lumen
**GO:0010494**	58	-0.621	7.67E-07	8.86E-04	cytoplasmic stress granule
**GO:0016469**	46	0.722	8.61E-07	9.54E-04	proton-transporting two-sector ATPase complex
**GO:0036464**	195	-0.323	8.45E-07	9.54E-04	cytoplasmic ribonucleoprotein granule
**GO:0034399**	133	-0.386	1.25E-06	1.36E-03	nuclear periphery
**GO:0070971**	19	-1.157	1.38E-06	1.47E-03	endoplasmic reticulum exit site
**GO:0031248**	91	-0.460	1.70E-06	1.75E-03	protein acetyltransferase complex
**GO:1902493**	91	-0.460	1.70E-06	1.75E-03	acetyltransferase complex
**GO:0016234**	80	-0.481	2.25E-06	2.26E-03	inclusion body
**GO:0000123**	81	-0.477	2.52E-06	2.49E-03	histone acetyltransferase complex
**GO:0042788**	30	0.990	4.09E-06	3.97E-03	polysomal ribosome
**GO:0005635**	436	-0.195	5.92E-06	5.64E-03	nuclear envelope
**GO:0005766**	138	0.343	7.37E-06	6.56E-03	primary lysosome
**GO:0042582**	138	0.343	7.37E-06	6.56E-03	azurophil granule
**GO:0005643**	75	-0.463	7.36E-06	6.56E-03	nuclear pore
**GO:0044322**	23	-0.957	7.28E-06	6.56E-03	endoplasmic reticulum quality control compartment
**GO:1902911**	104	-0.398	8.06E-06	7.06E-03	protein kinase complex
**GO:0030134**	73	-0.483	1.03E-04	8.89E-03	COPII-coated ER to Golgi transport vesicle
**GO:0016363**	105	-0.385	1.05E-04	8.94E-03	nuclear matrix
**GO:0005667**	405	-0.195	1.08E-04	9.03E-03	transcription regulator complex
**GO:0030667**	277	0.235	1.15E-04	9.46E-03	secretory granule membrane

**GO:0061695**	245	-0.248	1.26E-04	1.02E-02	transferase complex, transferring phosphorus-containing groups
**GO:1904949**	76	-0.451	1.33E-04	1.06E-02	ATPase complex
**GO:0005750**	10	1.026	1.84E-04	1.42E-02	mitochondrial respiratory chain complex III
**GO:0045275**	10	1.026	1.84E-04	1.42E-02	respiratory chain complex III
**GO:0030660**	94	-0.398	1.86E-04	1.42E-02	Golgi-associated vesicle membrane
**GO:0000314**	28	0.730	2.20E-04	1.64E-02	organellar small ribosomal subunit
**GO:0005763**	28	0.730	2.20E-04	1.64E-02	mitochondrial small ribosomal subunit
**GO:0070069**	25	0.797	2.25E-04	1.65E-02	cytochrome complex
**GO:0031984**	337	-0.203	2.28E-04	1.65E-02	organelle subcompartment
**GO:0045335**	123	0.336	2.62E-04	1.87E-02	phagocytic vesicle
**GO:0098791**	317	-0.205	3.13E-04	2.21E-02	Golgi apparatus subcompartment
**GO:0019908**	12	-1.119	3.20E-04	2.23E-02	nuclear cyclin-dependent protein kinase holoenzyme complex
**GO:0030135**	264	-0.220	4.18E-04	2.88E-02	coated vesicle
**GO:0009295**	43	-0.536	4.39E-04	2.94E-02	nucleoid
**GO:0042645**	43	-0.536	4.39E-04	2.94E-02	mitochondrial nucleoid
**GO:0043235**	382	0.181	4.65E-04	3.04E-02	receptor complex
**GO:0070603**	72	-0.423	4.61E-04	3.04E-02	SWI/SNF superfamily-type complex
**GO:0016605**	94	-0.362	5.18E-04	3.35E-02	PML body
**GO:0012507**	43	-0.583	5.32E-04	3.40E-02	ER to Golgi transport vesicle membrane
**GO:0009897**	283	0.207	5.55E-04	3.50E-02	external side of plasma membrane
**GO:0005788**	299	0.201	5.73E-04	3.58E-02	endoplasmic reticulum lumen
**GO:0030120**	56	-0.483	6.33E-04	3.91E-02	vesicle coat
**GO:0000790**	342	-0.186	6.65E-04	4.06E-02	nuclear chromatin
**GO:0031588**	13	-0.943	7.69E-04	4.64E-02	nucleotide-activated protein kinase complex
