# Supplementary material for: Transcriptome Profiling of the Liver in Nellore Cattle Phenotypically Divergent for RFI in Two Genetic Groups
Source: Animals (Basel). 2023 Jan 20;13(3):359. doi: 10.3390/ani13030359 (PMC9913155; doi:10.3390/ani13030359)
Supplement: Supplementary file 1 [file animals-13-00359-s001.zip › Supplementary Table S4.pdf]

Table S4. The significant biological processes related to Energy Metabolism. Protein turnover, and Redox homeostasis and immune system, in Genetic group 1 in the most efficient group.

Go Term	Number of genes	LOR*	p-value	padj	Biological process
**GO:0006490**	19	-0.866	2.3 $x10^{-04}$	$1.3x10^{-02}$	oligosaccharide-lipid intermediate biosynthetic process
**GO:0042158**	93	-0.497	2.4 $x10^{-06}$	$2.6x10^{-04}$	lipoprotein biosynthetic process
**GO:0042157**	121	-0.390	2.1 $x10^{-05}$	$1.3x10^{-04}$	lipoprotein metabolic process
**GO:0015980**	271	-0.377	1.0 $x10^{-09}$	2.5 $x10^{-07}$	energy derivation by oxidation of organic compounds
**GO:0006488**	18	-0.928	1.4 $x10^{-04}$	8.7 $x10^{-03}$	dolichol-linked oligosaccharide biosynthetic process
**GO:0010517**	68	0.384	1.6 $x10^{-03}$	6.7 $x10^{-02}$	regulation of phospholipase activity
**GO:0043550**	63	0.429	7.5 $x10^{-04}$	3.6 $x10^{-02}$	regulation of lipid kinase activity
**GO:0090218**	37	0.560	7.5 $x10^{-04}$	3.6 $x10^{-02}$	positive regulation of lipid kinase activity
**GO:0032981**	58	-1.082	2.0 $x10^{-22}$	3.6 $x10^{-19}$	mitochondrial respiratory chain complex I assembly
**GO:0033108**	87	-1.039	3.5 $x10^{-28}$	1.8 $x10^{-24}$	mitochondrial respiratory chain complex assembly
**GO:0042776**	19	-0.938	7.0 $x10^{-06}$	6.6 $x10^{-04}$	mitochondrial ATP synthesis coupled proton transport
**GO:0008535**	21	-0.895	9.4 $x10^{-06}$	8.5 $x10^{-04}$	respiratory chain complex IV assembly
**GO:0042773**	81	-0.833	1.2 $x10^{-12}$	5.1 $x10^{-10}$	ATP synthesis coupled electron transport
**GO:0022904**	100	-0.772	8.9 $x10^{-14}$	4.4 $x10^{-11}$	respiratory electron transport chain
**GO:0007260**	75	0.380	1.1 $x10^{-03}$	4.8 $x10^{-02}$	tyrosine phosphorylation of STAT protein
**GO:0071900**	492	0.369	1.6 $x10^{-15}$	9.7 $x10^{-13}$	regulation of protein serine/threonine kinase activity
**GO:0046777**	236	0.474	4.5 $x10^{-13}$	2.1 $x10^{-10}$	protein autophosphorylation
**GO:0032147**	324	0.387	6.8 $x10^{-12}$	2.6 $x10^{-09}$	activation of protein kinase activity
**GO:0031334**	234	0.440	4.0 $x10^{-11}$	1.4 $x10^{-08}$	positive regulation of protein-containing complex assembly
**GO:190382**	321	0.317	2.3 $x10^{-08}$	4.2 $x10^{-06}$	positive regulation of cellular protein localization

**9**					
**GO:0043393**	209	0.265	1.4 x10^{-04}	8.8 x10^{-03}	regulation of protein binding
**GO:0071634**	35	0.721	1.2 x10^{-05}	1.1 x10^{-03}	regulation of transforming growth factor beta production
**GO:0018107**	114	0.385	5.4 x10^{-05}	3.8 x10^{-03}	peptidyl-threonine phosphorylation
**GO:0007223**	37	0.561	1.0 x10^{-04}	2.8 x10^{-02}	Wnt signaling pathway, calcium modulating pathway
**GO:1901224**	75	0.493	6,4 x10^{-05}	2.0 x10^{-03}	positive regulation of NIK/NF-kappaB signaling
**GO:0000186**	55	-0.572	4.2 x10^{-05}	9.8 x10^{-03}	activation of MAPKK activity
**GO:0070371**	284	0.378	4.2 x10^{-10}	1.1 x10^{-07}	ERK1 and ERK2 cascade
**GO:0002764**	353	0.569	8.0 x10^{-25}	2.6 x10^{-21}	immune response-regulating signaling pathway
**GO:0002253**	402	0.471	7.6 x10^{-25}	7.8 x10^{-17}	activation of immune response
**GO:0045785**	386	0.568	1.9 x10^{-27}	8.6 x10^{-24}	positive regulation of cell adhesion
**GO:0001819**	356	0.534	2.1 x10^{-22}	3.6 x10^{-19}	positive regulation of cytokine production
**GO:0045787**	359	0.288	7.4 x10^{-08}	1.2 x10^{-05}	positive regulation of cell cycle
**GO:0097696**	139	0.325	1.5 x10^{-04}	9.3 x10^{-03}	receptor signaling pathway via STAT

*The LOR (Logarithm of Ratio) refer to LRFI group, this value categorizes each biological process in a classification in relation to the rest (total), allowing to identify biological processes, molecular mechanisms and significant cellular components and their regulation within the groups.
