# Supplementary material for: Transcriptome Profiling of the Liver in Nellore Cattle Phenotypically Divergent for RFI in Two Genetic Groups
Source: Animals (Basel). 2023 Jan 20;13(3):359. doi: 10.3390/ani13030359 (PMC9913155; doi:10.3390/ani13030359)
Supplement: Supplementary file 1 [file animals-13-00359-s001.zip › Supplementary Table S5.pdf]

Table S5. The significant biological processes related to Energy Metabolism. Protein turnover, and Redox homeostasis and immune system in genetic group 2 in the most efficient group.

Go Term	Number of genes	LOR*	p-value	padj	Biological process
GO:1901570	88	0.397	2.2 x10^{-04}	3.7 x10^{-02}	fatty acid derivative biosynthetic process
GO:0031663	54	0.570	5.9 x10^{-05}	1.3 x10^{-02}	lipopolysaccharide-mediated signaling pathway
GO:0005978	49	-0.549	1.5 x10^{-04}	2.7 x10^{-02}	glycogen biosynthetic process
GO:0009250	49	-0.549	1.5 x10^{-04}	2.7 x10^{-02}	glucan biosynthetic process
GO:0006110	72	-0.524	1.5 x10^{-05}	4.7 x10^{-03}	regulation of glycolytic process
GO:0002718	63	0.474	3.3 x10^{-04}	5.1 x10^{-03}	regulation of cytokine production involved in immune response
GO:0042149	40	-0.697	8.7 x10^{-06}	2.9 x10^{-02}	cellular response to glucose starvation
GO:0006120	46	1.141	2.9 x10^{-12}	6.8 x10^{-09}	mitochondrial electron transport, NADH to ubiquinone
GO:0042773	81	-1.199	2.5 x10^{-07}	1.7 x10^{-03}	ATP synthesis coupled electron transport
GO:1901685	19	1.047	1.3 x10^{-07}	1.0 x10^{-04}	glutathione derivative metabolic process
GO:1901687	19	1.047	1.3 x10^{-07}	1.0 x10^{-04}	glutathione derivative biosynthetic process
GO:1990748	102	0.362	2.6 x10^{-04}	4.2 x10^{-02}	cellular detoxification
GO:0097237	110	0.357	1.9 x10^{-04}	3.2 x10^{-02}	cellular response to toxic substance
GO:0006614	94	1.139	6.3 x10^{-18}	1.2 x10^{-13}	SRP-dependent cotranslational protein targeting to membrane
GO:2001056	135	0.408	2.9 x10^{-06}	1.3 x10^{-03}	positive regulation of cysteine-type endopeptidase activity
GO:0010952	172	0.350	6.4 x10^{-06}	2.4 x10^{-03}	positive regulation of peptidase activity
GO:0006577	17	-0.736	1.2 x10^{-03}	1.4 x10^{-01}	amino-acid betaine metabolic process
GO:0052548	358	0.225	2.6 x10^{-05}	6.6 x10^{-03}	regulation of endopeptidase activity
GO:2001056	135	0.408	2.9 x10^{-06}	1.3 x10^{-03}	positive regulation of cysteine-type endopeptidase activity
GO:0043280	118	0.408	1.2 x10^{-05}	3.8 x10^{-03}	positive regulation of cysteine-type endopeptidase activity involved in apoptotic process
GO:0007249	253	0.222	4.7 x10^{-04}	6.5 x10^{-02}	I-kappaB kinase/NF-kappaB signaling
GO:0043123	176	0.276	2.9 x10^{-04}	4.5 x10^{-02}	positive regulation of I-kappaB kinase/NF-kappaB signaling
GO:0000186	55	-0.572	4.2 x10^{-05}	9.8 x10^{-03}	activation of MAPKK activity
GO:0032963	98	0.594	7.8 x10^{-09}	8.8 x10^{-06}	collagen metabolic process
GO:0030574	42	0.619	5.2 x10^{-05}	1.1 x10^{-03}	collagen catabolic process
GO:2000251	19	0.827	2.1 x10^{-04}	3.5 x10^{-02}	positive regulation of actin cytoskeleton reorganization

**GO:0001516**	24	0.794	1.6 $x10^{-04}$	2.8 $x10^{-02}$	prostaglandin biosynthetic process
**GO:0018107**	114	-0.364	1.4 $x10^{-04}$	2.5 $x10^{-02}$	peptidyl-threonine phosphorylation
**GO:0052547**	383	0.238	4.6 $x10^{-06}$	1.9 $x10^{-03}$	regulation of peptidase activity
**GO:0002283**	449	0.328	1.3 $x10^{-06}$	2.6 $x10^{-08}$	neutrophil activation involved in immune response
**GO:0002446**	460	0.329	6.4 $x10^{-12}$	1.4 $x10^{-08}$	neutrophil mediated immunity
**GO:0002694**	476	0.283	1.6 $x10^{-09}$	2.1 $x10^{-06}$	regulation of leukocyte activation
**GO:0050867**	306	0.349	2.3 $x10^{-09}$	3.0 $x10^{-08}$	positive regulation of cell activation
**GO:0002250**	352	0.279	2.8 $x10^{-07}$	1-9 $x10^{-04}$	adaptive immune response
**GO:0042110**	431	0.226	3.8 $x10^{-06}$	1.6 $x10^{-03}$	T cell activation
**GO:0033006**	30	0.863	7.5 $x10^{-06}$	2.6 $x10^{-03}$	regulation of mast cell activation involved in immune response
**GO:0002697**	351	0.241	8.7 $x10^{-07}$	2.9 $x10^{-03}$	regulation of immune effector process

*The LOR (Logarithm Of Ratio) refer to LRFI group, this value positions each biological process in a classification in relation to the rest (total), being able to identify biological processes, molecular mechanisms and significant cellular components and their regulation within the groups.
